# Untargeted Metabolomics and Targeted Phytohormone Profiling of Sweet Aloes (*Euphorbia neriifolia*) from Guyana: An Assessment of Asthma Therapy Potential in Leaf Extracts and Latex

**DOI:** 10.3390/metabo15030177

**Published:** 2025-03-05

**Authors:** Malaika Persaud, Ainsely Lewis, Anna Kisiala, Ewart Smith, Zeynab Azimychetabi, Tamanna Sultana, Suresh S. Narine, R. J. Neil Emery

**Affiliations:** 1 Sustainability Studies Graduate Program, Faculty of Arts and Science, Trent University, Peterborough, ON K9J 0G2, Canada; malaikapersaud@alumni.trentu.ca; 2 Department of Biology, Trent University, Peterborough, ON K9J 0G2, Canada; annakisiala@trentu.ca (A.K.); nemery@trentu.ca (R.J.N.E.); 3 Department of Biology, University of Toronto Mississauga, Mississauga, ON L5L 1C6, Canada; 4 Environmental and Life Sciences Graduate Program, Trent University, Peterborough, ON K9J 0G2, Canada; ewartsmith@trentu.ca (E.S.); zeynazimychetabi@trentu.ca (Z.A.); 5 Department of Chemistry, Trent University, Peterborough, ON K9J 0G2, Canada; tamannasultana@trentu.ca; 6 Trent Centre for Biomaterials Research, Trent University, Peterborough, ON K9J 0G2, Canada; sureshnarine@trentu.ca; 7 Departments of Physics & Astronomy and Chemistry, Trent University, Peterborough, ON K9J 0G2, Canada

**Keywords:** asthma, cytokinins, mass-spectrometry, metabolomics, phytohormones, traditional therapy

## Abstract

**Background/Objectives:** *Euphorbia neriifolia* is a succulent plant from the therapeutically rich family of Euphorbia comprising 2000 species globally. *E. neriifolia* is used in Indigenous Guyanese asthma therapy. **Methods:** To investigate *E. neriifolia’s* therapeutic potential, traditionally heated leaf, simple leaf, and latex extracts were evaluated for phytohormones and therapeutic compounds. Full scan, data-dependent acquisition, and parallel reaction monitoring modes via liquid chromatography Orbitrap mass spectrometry were used for screening. **Results:** Pathway analysis of putative features from all extracts revealed a bias towards the phenylpropanoid, terpenoid, and flavonoid biosynthetic pathways. A total of 850 compounds were annotated using various bioinformatics tools, ranging from confidence levels 1 to 3. Lipids and lipid-like molecules (34.35%), benzenoids (10.24%), organic acids and derivatives (12%), organoheterocyclic compounds (12%), and phenylpropanoids and polyketides (10.35%) dominated the contribution of compounds among the 13 superclasses. Semi-targeted screening revealed 14 out of 16 literature-relevant therapeutic metabolites detected, with greater upregulation in traditional heated extracts. Targeted screening of 39 phytohormones resulted in 25 being detected and quantified. Simple leaf extract displayed 4.4 and 45 times greater phytohormone levels than traditional heated leaf and latex extracts, respectively. Simple leaf extracts had the greatest nucleotide and riboside cytokinin and acidic phytohormone levels. In contrast, traditional heated extracts exhibited the highest free base and glucoside cytokinin levels and uniquely contained methylthiolated and aromatic cytokinins while lacking acidic phytohormones. Latex samples had trace gibberellic acid levels, the lowest free base, riboside, and nucleotide levels, with absences of aromatic, glucoside, or methylthiolated cytokinin forms. **Conclusions:** In addition to metabolites with possible therapeutic value for asthma treatment, we present the first look at cytokinin phytohormones in the species and *Euphorbia* genus alongside metabolite screening to present a comprehensive assessment of heated leaf extract used in Indigenous Guyanese asthma therapy.

## 1. Introduction

*Euphorbia neriifolia* is a species from the Euphorbia genus that comprises 2000 species found across various parts of the world belonging to the family Euphorbiaceae [1,2]. This family is known to comprise a therapeutically rich diversity of plants [3]. Many Euphorbia species have been researched extensively due to their unique morphological characteristics and ecological importance for horticulture and biofuel production and medicinal properties. Some species have garnered significant attention, including *E. peplus* [4], *E. hirta* [5], *E. tirucalli* [6], *E. antiquorum* [7], *E. resinifera* [8], *E. cotinifolia* [9], *E. helioscopia* [10], *E. lathyrism* [11], *E. characias* [12], *E. ingens* [13], and *E. antisyphilitica* [14]. The treatment of numerous diseases globally indicates its use as an anti-convulsant, anti-diabetic, anti-hypertensive, anti-inflammatory, antimalarial, antimicrobial, anti-plasmodial, anti-oxidant, anti-tumor, bronchitis, syphilis, cholesterol-lowering, contraceptive, antitussives, emetic, pneumonia and anti-asthmatic agents or as poisons and anti-venom [1,15]. While *E. hirta* is referred to as the asthma plant, *E. neriifolia* is used for this purpose in Guyana where the common names are sweet aloes and sahure [16,17].

*E. neriifolia* is a xerophytic succulent noted to be native to Southeast Asia [18]. Several studies have investigated its pharmacological properties and identified potential uses [15,19,20]. Extracts demonstrated antioxidant activity [21,22,23,24], which can offer protection against oxidative stress-induced damage, among many other functions, as identified among species within the *Euphorbia* genus [19,23]. Despite its potential therapeutic benefits, toxic compounds, such as diterpenoids [25], which can cause irritation and inflammation if ingested or applied topically, were detected in the latex [26]. Nevertheless, the traditional use of *Euphorbia neriifolia* has shown promising results in studies that suggest it could be a valuable source of medicine in the future [27]. The use of rat models to test applicability to asthma therapy [15] gives further incentive for the exploration of *E. neriifolia* for use in this condition, which has a potential far-reaching global impact.

Asthma is a chronic, non-communicable respiratory disease that affects millions of people worldwide, with a high prevalence in children [28]. Complex interactions between various mediators and cell types cause increased inflammation, bronchoconstriction, and overall airway hyperresponsiveness [29,30]. Oxidative stress and reactive oxygen species, in addition to cytokine triggers, are implicated [31,32,33]. These effects precipitate the characteristic symptoms of cough, wheezing, chest tightness, and breathing difficulty in asthma [33]. IgE and Interleukins IL-4, IL-5, IL-10, and IL-13 are implicated as direct pro-inflammatory cytokines, while there is a likelihood of indirect mechanistic trigger by IL-6, which was previously not thought to be involved in the pathogenesis [31]. Hormone levels were also shown to have an impact on asthma [34], but the etiology of asthma remains unknown. Current medications include B-agonists and corticosteroids [35], which potentiate serious adverse effects [36], therefore necessitating the exploration of alternative therapies.

*E. neriifolia* has been purportedly used traditionally without adverse effects in babies and children in Guyana [17], while latex is reportedly used elsewhere [15]. While research has been conducted on *E. neriifolia* leaves globally, most were focused on exploring powdered leaves after shade-drying or oven-drying and latex fractions. These were also not performed in the context of metabolites and involvement in asthma. Furthermore, *E. neriifolia* was determined as one of the least-researched members of the genus. Additionally, no focus was placed on the potential role of phytohormones, such as abscisic acid, auxins, gibberellins, jasmonates, and cytokinins, which studies show play a role in disease management [37,38].

Phytohormones, first defined in 1948 to differentiate them from animal hormones, are signaling molecules found in plants that regulate their growth and development processes [39]. They are grouped as cytokinins like kinetin (K) and zeatin (Z) and acidic hormones like auxins, gibberellins (GAs), abscisic acid (ABA), salicylic acids (SA), and brassinosteroids [39,40]. Cytokinins (CKs), not to be confused with cytokines, are adenine-derived compounds substituted at the N**^6^** position with an isoprenoid or aromatic side-chain [41] that regulate plant growth and adaptation to environmental conditions and are classified as isoprenoids or aromatics [42,43].

Isoprenoid CKs are more abundant in nature, while aromatic CKs are limited to very few plant species [43]. The aromatics include the first cytokinin discovered in 1955, kinetin (a.k.a 6-furfuryl adenine) and its riboside form, kinetin riboside (KR) and N**^6^**-benzyl adenine (BA), and its corresponding riboside N**^6^**-benzyadenosine (BAR) [44]. Through various processes, the interconversion of cytokinin molecules yields fractions of free bases, ribosides, nucleotides, glucosides [44], and methylthiolated derivatives [45].

Cytokinins and their derivatives were demonstrated to be neuroprotective and immunomodulatory and affect the division of mammalian cells by promoting or inhibiting cell division and inducing cell differentiation, among other effects [37,46,47]. Kinetin, in particular, has generated significant interest in human therapy within the past few decades in oxidative stress, DNA repair, neurodegenerative disorders like Huntington’s disease [41,48,49,50,51], and slowing aging of human cells. Benzyladenine (BA) influences oxidative stress parameters [49], and the riboside is noted for high toxicity to cell lines [41]. N**^6^**-isopentenyladenosine (iPR) also demonstrates cytotoxicity.

These isoprenoid fractions include further cytokinin types: zeatin (Z)—cis and trans forms (cZ and *t*Z), dihydrozeatin (DZ), and isopentenyladenine (iP) [40]. Zeatin and zeatin ribosides have received increased interest for gerontomodulatory effects, including reducing UV potentiated aquaporin 3 downregulation, thereby reducing photoaging in human skin keratinocytes [52,53]. The O-glucoside form of zeatin was reported to have a potential role in the treatment of neurodegenerative diseases [48]. This was different in a cancer report where O-Glucosides were reported as functionally less active on cell lines [43]. Cytokinins have effects on mammalian cells where they can be taken up and changed in addition to having applications in cancer, aging, and skin conditions [41,54,55,56]. In plants, free base and riboside molecules are credited as active fractions, while nucleotides are considered inactive. Tissue lines showed sensitivity to the riboside forms of iP, cZ, Kinetin, and Benzyladenine and the nucleotide form of cytokinin [43]. The freebase, methylthiolated, O-and-N glucoside forms of cytokinins possess little or no activity in human cell lines compared to NT and RB fractions that exhibit pharmacologic effects in cancer and Huntington’s [43]. Kinetin riboside (KR) is converted to kinetin riboside nucleotide (KRNT) as triphosphate (Kinetin triphosphate; KTP) by adenine phosphoribosyltransferase (APRT) enzyme, which then exerts its effects on DNA repair and reactive oxygen species clean up [46]. Considering this, cytokinin and phytohormone studies in the genus have only targeted exogenous applications of hormones for effects on plant growth or in vitro regeneration to date, leaving the area of endogenous hormone use unexplored.

The application of acidic hormones (i.e., (ABA), (GAs), indole-3-acetic acid (IAA), jasmonic acid (JA), and salicylic acid (SA)) in human health was explored. Following the debate over the endogenous presence of ABA in mammals and proof [57], current research points to the roles of ABA in humans in stimulating glucose uptake for diabetic control as well as a marker for asthma and chronic obstructive pulmonary disease [58,59,60,61,62]. Antimicrobial properties were noticed for three enantiomers of GAs, where GA**_3_** showed good activity with lower GA**_7_** and GA**_9_** [63]. Additionally, anti-inflammatory properties were detected [64]. Recent studies point to the cell regeneration properties of IAA [65] and the anti-inflammatory properties of IAA in mouse models for ankylosing spondylitis [66]. Jasmonic acid is also indicated in anti-aging skin applications, cancer, and anti-inflammation processes [67]. The role of the non-steroidal anti-inflammatory agent aspirin is potentiated by the potent metabolite SA [37]. The role of aspirin in health has been well-documented since its discovery, and it remains widely used for blood thinning and cardiovascular diseases [68]. All the acidic phytohormones have, therefore, been shown to have some impact on human disease.

Given the global use of *E. neriifolia* in multiple health conditions (Table 1), the effect of phytohormones and their roles have been under investigated. There is deficient information on the sample types in this respect, especially on the traditionally heated extract. Therefore, this study was conducted to explore potentially therapeutic phytochemicals and phytohormones in the traditionally heated extract, as well as simple extract from the leaves and latex. This is the first report, to date, to showcase levels of the cytokinin phytohormone class in *Euphorbia neriifolia* alongside other annotated metabolites.

## 2. Materials and Methods

### 2.1. Euphorbia neriifolia—Plant Material

Sweet aloe plant stems were obtained from Guyana at geographic coordinates 6°43′46″ N 58°14′17″ W and transported to Canada following the regulatory and phytosanitary procedures. Four stems were planted one per pot in soaked soil in Sunshine Professional Growing Mix (Mix #1 Sungrow Horticulture, Canada). Growth occurred under a 16-h light photoperiod, 60–80% RH, 24–27 °C, 8-h dark photoperiod, 60–80% RH, at 19–22 °C in the Aurora Greenhouse (Conviron, Canada) [71] at Trent University from September 2021 to June 2022.

### 2.2. Sample Preparation of Plant Material

Leaves were randomly sampled from three plants to make biological replicates: (*n* = 5) for simple fresh leaf extract (SE) and (*n* = 5) for traditionally heated leaf extract (TE). For Extracted Latex (LE) samples, (*n* = 5) exudates were collected from leaves detached from stems and at points of stems after separating leaves and cutting stems. Approximately 0.100 g fresh weight of leaf tissues (SE) were cut and placed in 2 mL safe lock centrifuge tubes, while 0.100 g (LE) samples were collected in 5 mL tubes. Leaves for TE samples were heated to 250 °C for 15 **s** and then squeezed to yield the liquid traditional extract (TE). This procedure mimics the preparation normally performed when being prepared for children by Guyanese Indigenous persons as per the Indigenous traditional method [17]. The TE samples were collected into separate 5 mL tubes and centrifuged for 10 min at 5000 rpm, after which 2 mL were transferred to individual 15 mL tubes. SE, LE, and TE samples were flash-frozen in liquid nitrogen, and SE was stored at −80 °C until further processing. The LE and TE samples were freeze-dried (LabConco Free Zone lyophilizer; Kansas City, MO, USA) and stored at −20 °C.

### 2.3. Solid Phase Extraction for Metabolite and Phytohormone Purification

Phytohormone and other metabolite extractions were carried out in a sequential extraction process to obtain different phytohormone fractions using modified methods previously published [42,72] to facilitate the multi-extraction of 39 cytokinins and acidic hormones: (Abscisic acid (ABA), gibberellins (i.e., GA_1_. GA_4_, GA_7_, GA_9_, GA_20_), indole-3-acetic acid (IAA), jasmonic acid (JA), and salicylic acid (SA)) from a single plant sample. To enable phytohormone quantification through the isotope dilution technique, internal standards (IS) of deuterated phytohormones were added to each sample constituting 1 mL 50% ice-cold acetonitrile (ACN): (60.1 ng ABA ([^2^H_4_] ABA) (PBI, Saskatoon, Canada), 10 ng each of acidic phytohormones IAA and SA (OLChelmm, Olomouc, Czech Republic), 20 ng each of gibberellins (i.e., GA_1_. GA_4_, GA_7_, GA_9_, GA_20_) and 10 ng each of deuterated cytokinin standards consisting of aromatic, methylthiolated, and glucoside forms. The CKs scanned for are as follows (Table 2): benzyladenine (BA), benzyladenine riboside (BAR), kinetin (KIN), cis-zeatin (cZ), cis-zeatin riboside (cZR), cis-zeatin-9-glucoside (cZ9G), cis-zeatin nucleotide (cZNT), cis-zeatin O-glucoside (cZOG), cis-zeatin riboside-O-glucoside (cZROG), dihydrozeatin (DZ), dihydrozeatin nucleotide (DZNT), dihydrozeatin-O-glucoside (DZOG), dihydrozeatin riboside (DZR), dihydrozeatin riboside-O-glucoside (DZROG), dihydrozeatin-9-N-glucoside (DZ9G), isopentenyladenine (iP), isopentenyladenine nucleotide (iPNT), isopentenyladenine-9-glucoside (iP9G), isopentenyladenosine (iPR), 2-methylthio-isopentenyladenine (2MeSiP), 2-methylthio-isopentenyladenosine (2MeSiPR), 2-methylthio-zeatin (2MeSZ), 2-methylthio-zeatin riboside (2MeSZR), *trans*-zeatin (*t*Z), *trans*-zeatin riboside (*t*ZR), trans-zeatin-9-glucoside (tZ9G), *trans*-zeatin nucleotide (*t*ZNT), *trans*-zeatin O-glucoside (*t*ZOG), and *trans*-zeatin riboside-O-glucoside (*t*ZROG) [73]. While JA was not added as an internal standard, ([^2^H_4_] ABA) was used as a standard for JA quantification. METLIN [74] (<https://metlin.scripps.edu>; accessed between 1 January 2023 to 31 January 2023) was used to confirm JA in plant samples based on the protonated monoisotopic mass.

SE samples containing two zirconium beads (Comeau Technique Ltd., Montréal, QC, Canada) were homogenized using the Retsch 300 ball mill grinder (Haan, Germany) at 25 Hz for 5 min in a 4 °C cold room. All samples (i.e., SE, TE, and LE) were vortexed and then refrigerated at −20 °C for overnight passive extraction.

The samples were transferred on ice from the −20 °C freezer, and the TE and LE samples were centrifuged at 5000 rpm for 5 min, while the SE samples were centrifuged at 10,000 rpm for 10 min (ThermoScientific Sorvall ST 16 centrifuge; ThermoFisher Scientific, San Jose, CA, USA). The supernatants were collected into 2 mL tubes and combined with subsequent supernatants collected upon washing samples with two sets of 500 μL of cold ACN and treated as before. Three method-blank samples were prepared and extracted similarly to plant samples. The supernatants and blank samples were vortexed and centrifuged at 10,000 rpm for 5 min.

HLB cartridges (VIOLET™ 200 mg/6 mL, 40 μm; Canadian Life Sciences; Peterborough, ON, Canada) were sequentially preconditioned with methanol and ddH_2_O water. Columns were equilibrated with 50% ACN before loading supernatants, followed by 2 mL of 30% aqueous ACN. Each extract was divided into two samples; one set was for CKs and the other fraction for derivatization of acidic phytohormones (method in following Section 2.4) and other metabolites. All samples were evaporated to dryness overnight at ambient temperature in a speed vacuum centrifuge concentrator (Thermo Savant UVS 400a; ThermoFisher Scientific, Berlin, Germany).

#### 2.3.1. Derivatization of Acidic Phytohormones

The dried phytohormone fraction containing metabolites from the initial HLB extraction was used to derivatize acidic phytohormones (modified from [75]). Dried samples were reconstituted with the following reagents added in order: 75 μL of 1-propanol (Fisher Scientific; Ottawa, ON, Canada), 20 μL of ddH_2_0 water, 5 μL of 500 mM bromocholine (Fisher Scientific; Ottawa, ON, Canada) in 70% ACN and 1 μL of triethylamine (Fisher Scientific; Ottawa, ON, Canada). These mixed samples were vortexed, and tubes with punched holes were suspended in a water bath at 80 °C for 130 min, then moved immediately to ice and dried under the speed vacuum concentrator for 3 h [75].

#### 2.3.2. Solid Phase Extraction—Sequential Elution of CK Fractions: Free Base, Riboside, Methylthiolated, and Nucleotide Forms

Samples of the supernatants for CK sequential elution were dried overnight using a speed vacuum centrifuge. Residues were reconstituted in 1 mL of 1 M formic acid to promote complete protonation of CKs, vortexed, and centrifuged at 5000 rpm–10,000 rpm for 5 min. The redissolved residues were run through mixed-mode cation exchange SPE cartridges (IRIS™ MCX 200 mg/6.0 mL, 40 μm; Canadian Life Sciences, Peterborough, ON, Canada) sequentially preconditioned using MeOH and 1 M formic acid (HCOOH) as previously described [76]. Nucleotides were eluted first with 0.35 M ammonium hydroxide (NH_4_OH), while free-base, methylthiolated, glucoside, and riboside cytokinin forms were eluted together using 0.35 M ammonium hydroxide in 60% methanol (NH_4_OH: MeOH (40:60 *v*/*v*)), all separating according to polarity and cationic properties [42]. All collected fractions were dried by the speed vacuum centrifuge.

As nucleotide CK forms cannot be detected with this mass spectrometry methodology, they were dephosphorylated [42]. Samples prepared for CK nucleotide extraction were redissolved in 1.0 mL of 0.1 M ethanolamine and vortexed before being phosphatased. Twelve (12) μL of phosphatase enzyme (New England BioLabs Ltd., Pickering, Canada) were added to the samples, vortexed, incubated at 37 °C overnight, and dried for 6 h in a speed vacuum centrifuge. The samples were redissolved in 1.5 mL of ddH_2_O water, vortexed, and centrifuged for 10 mins at 10,000 rpm. C18 SPE cartridges (C18, 6 cc, 500 mg; Canadian Life Sciences, Peterborough, ON, Canada) were preconditioned with methanol and ddH_2_O before samples were introduced and allowed to flow through the column by gravity. CK NT elution was conducted with 1.25 mL of 80% MeOH and collected samples were speed vacuumed overnight.

Dried samples belonging to all fraction types (CK fractions and acidic phytohormones) were redissolved in 300 μL of starting conditions constituting AcOH, ACN, and ddH_2_O in a ratio of 0.08:5:94.92 (*v*:*v*:*v*), respectively. Samples were vortexed and centrifuged at 5000 rpm for 10 min before being transferred to 2 mL clear vials with 350 μL glass inserts and stored at −20 °C until mass spectrometric analysis.

### 2.4. Ultrahigh Pressure Liquid Chromatography-Mass Spectrometry Analysis of Phytohormones and Metabolites

The phytohormone samples were analyzed using a Q Exactive orbitrap mass spectrometer in (ThermoScientific; Waltham, MA, USA) equipped with a heated electrospray ionization source (HESI-II) coupled to a Thermo Dionex Ultimate 3000 UHPLC (ThermoScientific; San Jose, CA, USA) [42]. The chromatographic separation of CKs was accomplished with an HGP-3400RS dual pump and WPS-3000 autosampler equipped with a Kinetex C18 column (2.1 i.d × 50 mm, 2.6 μm particle size, Phenomenex, Torrace, CA, USA) operated at an approximate room temperature of 22 °C. The instrument control was achieved with Chromeleon 6.8 Chromatography Data System software (ThermoScientific; Ottawa, ON, Canada).

All phytohormone fractions were eluted with component A comprising ddH_2_0 with 0.08% CH_3_COOH and component B comprising CH_3_CN with 0.08% CH_3_COOH at a flow rate of 0.5 mL/min. The CK fractions were eluted with a multi-step gradient. The starting condition, consisting of 5% B, was held at 0.5 min, increasing linearly to 45% B over 4.5 min, followed by an increase to 95% B over 6.5 min; 95% B was held constant for 1 min before returning to starting conditions for 2 min for column re-equilibration. The total run time for CKs was 8.2 **min** with an injection volume of 25 μL [42,73].

A full scan in positive ionization mode was conducted for the acidic phytohormones and untargeted metabolites with a run time of 15 **min** on the eluted analytes in the Q Exactive Orbitrap high-resolution mass spectrometer with modifications [42]. A mass range of *m*/*z* 100 to 700 was used at a resolution of 140,000 at *m*/*z* 200 full width at half minimum (FWHM), with automatic gain control (AGC) target of 3 × 10^6^, and maximum injection time (IT) of 524 ms. A representative sample from the replicates (i.e., SE, TE, and LE) was used for data-dependent tandem mass spectrometry (ddms^2^). For samples derived from the HLB extraction for acidic phytohormones and metabolites, the full scan was performed at a resolution of 70,000 with an AGC target of 5 × 10^4^. For fragmentation, it was performed at a resolution of 17,500 with an AGC target of 5 × 10^5^. The fragmentation was triggered at a loop count of 10 (top 10 most intense peaks per scan), with a precursor isolation window of 1 *m*/*z*. The maximum IT was 64 ms.

For the cytokinin samples, acquisition was performed in positive ion mode, and data were acquired via parallel reaction monitoring (PRM) [42]. PRM data were acquired at a resolution of 35,000 FWHM at *m*/*z* 200. PRM parameters consisted of an automatic gain control (AGC) of 3 × 10^6^ and a maximum IT of 128 ms. The precursor isolation window width was *m*/*z* 1.2. The normalized collision energy (NCE) was individually optimized for each compound by stepwise increments, where at least 10% of the unfragmented precursor ion was retained.

### 2.5. Data Analysis

#### 2.5.1. Data Analysis for Mass Spectrometry-Based Metabolomics

**Figure 1:**
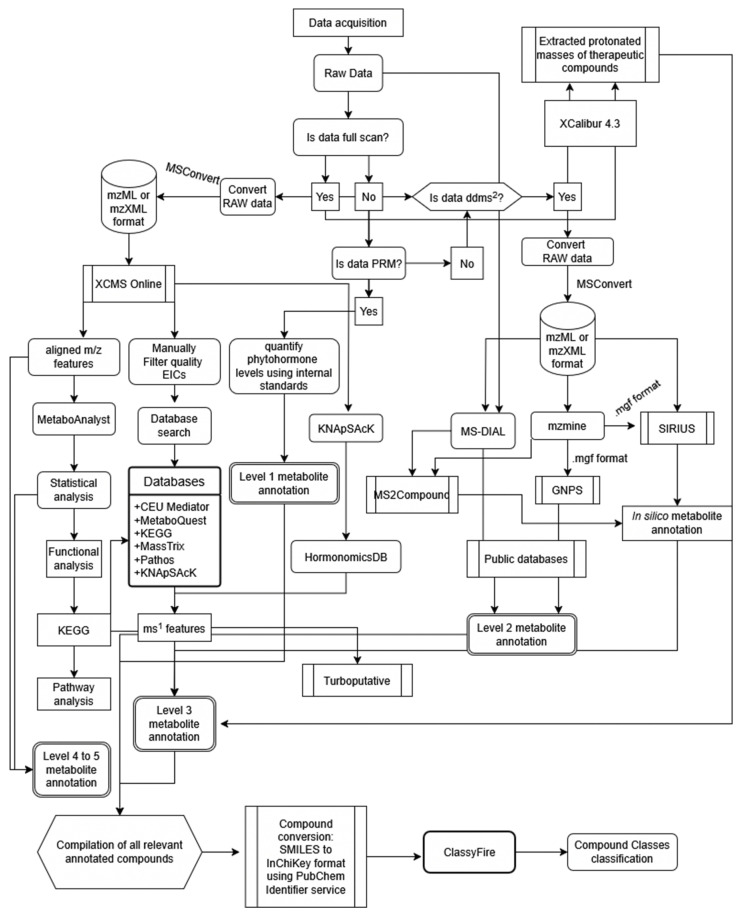
Flowchart of data analysis workflow. The flowchart was created using https://app.diagrams.net (accessed between 13 January 2025 to 28 February 2025). Annotation levels were assigned as previously published [81,82].

The LC-MS raw data acquired during sample analysis via UHPLC–MS were exported as mzXML files using the MSConvert module in Proteowizard 3.022 (Figure 1) [77,78] and preprocessed using the multigroup option in XCMS Online [79,80] (accessed between 16 January 2023 to 30 January 2023) for peak detection, retention time correction, and alignment of the metabolites detected in the UHPLC–MS analysis. Peak detection was performed using centWave peak detection (∆*m*/*z* = 10 ppm; minimum peak width, 10 s; maximum peak width, 60 s) and mzwid = 0.015, minfrac = 0.5, bw = 10 for alignment of retention time. The obiwarp method was used for retention time correction. The signal-to-noise ratio was set to 10:1. The Kruskal–Wallis non-parametric test was used for statistical analysis.

#### 2.5.2. Statistical Analysis and Differential Metabolite Selection for Comparative Analysis

The processed data, in the form of an Excel worksheet file derived from XCMS Online, were separated into .csv files corresponding to sample type (i.e., SE (unheated), TE (heated), and LE (latex extract) samples. These sample types were put in folders according to pairwise comparisons (i.e., SE vs. TE and SE vs. LE) and then zipped together. The zipped folder was uploaded to MetaboAnalyst 5.0 [83] (https://www.metaboanalyst.ca; accessed between 19 January 2023 to 5 February 2023) and submitted to the Statistical Analysis (one factor) module. Mass and retention time tolerances of 0.005 *m*/*z* and 30 **s** were chosen, respectively. For data filtering, the inter-quantile range function was used. For sample normalization, normalization by sum was chosen. Log transformation was chosen for data transformation, and Pareto scaling was chosen as the option for data scaling. This was performed to reduce the skewness of the data and reduce the mask effects [84].

Principal Component Analysis (PCA) and Partial Least Squares Discriminant Analysis (PLS-DA) were performed on the normalized datasets [84]. These statistical methods project the variables to a new space to determine which variables in the complex dataset are responsible for observations being classified into groups or categories. The Variable Importance in Projection (VIP) arising from PLS-DA analysis was used to decipher features responsible for the separation of sample types. Features refer to distinct signals detected from mass spectrometric analysis generated by different molecules (putative metabolites) present in the biological sample that are characterized by their mass-to-charge ratio (*m*/*z*) and retention time. At this stage, the identity of the metabolite that generated the signal is unknown; thus, the feature is used instead of metabolite. Univariate statistics (i.e., *t*-test) was applied to calculate the statistical significance of *m*/*z* values in treatment comparisons (i.e., the SE (unheated control) vs. TE (heated) and the unheated control (SE) vs. the milky latex (LE).

Volcano plot analyses were performed to determine significantly changed features (i.e., up-or downregulated features) occurring in the TE and LE compared to the control sample SE. As volcano plots provide a summary of *t*-test and fold change (FC) analysis, a log_2_FC > 1 or log_2_FC < −1 was chosen with a false detection rate (FDR) < 0.05. Figures for volcano plots were regenerated using VolcaNoseR [85].

#### 2.5.3. Untargeted Metabolite Identification

##### 2.5.3.1. Database Query Using Quality EICs Generated from XCMS Online

For the respective treatments (SE, TE, and LE), the single module was selected in XCMS online to verify quality peaks from the extracted ion chromatograms (EICs) generated from XCMS Online. These EICs images were visually identified, and the corresponding *m*/*z* (i.e., *m*/*z* medium stylized as ”mzmed” as generated by XCMS Online) that is within the EIC *m*/*z* extracted range was selected from the .xlsx file downloaded from XCMS online processing.

To look at the chemodiversity of features, Venn diagrams were used as a visualization tool. All features were detected using the single module in XCMS online, and all quality EICs were subjected to Venn diagram analysis. Only shared *m*/*z* values for each treatment were used in the Venn diagram analysis. Duplicate *m*/*z* values from shared *m*/*z* values from each treatment type were automatically removed using InteractiVenn [86] or EVenn [87] or Microsoft Excel.

The *m*/*z* values, which would be the observed masses arising from quality XCMS-generated EICs, were queried against the theoretical masses of different databases such as Pathos [88] (<https://motif.gla.ac.uk/Pathos/>; accessed on 15 February 2025), MassTrix [89] (formerly <https://masstrix.org>; accessed on 16 January 2023), MetaboQuest (<http://tools.omicscraft.com/MetaboQuest/> or <https://tools.omicscraft.com/aiSysMet/>, developed from MetaboSearch [90]; accessed on 07 February 2023), and CEU Mass Mediator 3.0 [91] (Figure 1; Section 2.5.3.3). Putative metabolites that were unique and shared between the milky latex, the unheated extract (SE), and the heated extracts (TE) were determined, and as a cut-off, metabolites with KEGG IDs were selected. As indicated, putative metabolites refer to potential assignments for previously generated features based on matching their characteristics (best match basis) to known metabolites in databases or reference libraries. This does not signify definitive confirmation. Only [M+H]^+^ adducts were considered for annotation.

KNApSAcK (<https://www.knapsackfamily.com/KNApSAcK/>; accessed between 21 February 2023 and 22 February 2023) [92] was also primarily searched to check for compounds within the Euphorbia species and also to cross-reference with other databases, especially in tandem mass spectrometry (Section 2.5.4).

##### 2.5.3.2. KEGG Functional and Pathway Analyses Modules in MetaboAnalyst 5.0

In addition, using the *m*/*z*, retention time (t_R_), and *p*-values of all detected aligned features generated by the XCMS Online multimode module were put in a .csv file to be analyzed using functional analysis to query whether there may be metabolites of interest in *E. neriifolia*. Annotation was performed with a 5-ppm mass tolerance using the mummichog algorithm [93] with a 0.05 *p*-value cut-off [94] with *Arabidopsis thaliana* reference metabolome in the Kyoto Encyclopaedia of Genes and Genomes (KEGG) [95]. Annotated compounds were then screened via the pathway and enrichment analyses modules in MetaboAnalyst 5.0 (Figure 1; accessed between 19 January 2023 to 5 February 2023).

Metabolites over-represented on the pathway were ranked using hypergeometric testing, with correction performed using the Benjamini–Hochberg false discovery rate (FDR) [94]. The KEGG IDs generated using functional analysis were used for determining putative compounds. Putative metabolites were identified according to the metabolite annotation level guidelines (levels 1–5) previously published [81,82,96].

The KEGG IDs of the matched compounds were submitted to the pathway analysis module in MetaboAnalyst 5.0, which uses the hypergeometric test [97] and the latest KEGG version of the *A. thaliana* pathway library [95]. It should be noted that the exact *m*/*z* values of the compounds were inputted into the MetaboAnalyst 5.0, and that peak quality was not determined. The Pathway analysis gives an idea of the putative compounds based on exact mass within a 5-ppm error margin.

##### 2.5.3.3. MS^1^ Mass Query Using Databases

MassTrix: It should be noted that the server for MassTrix [89] is no longer available online (formerly <https://masstrix.org>; accessed on 16 January 2023) but is available via the R package masstrixR <https://rdrr.io/github/michaelwitting/masstrixR/> (accessed on 24 February 2025). The analysis conducted for this manuscript occurred prior to the server being taken offline. KEGG and LipidMaps databases within a 5-ppm error were queried via MassTrix with [M+H]+ adduct selected.

CEU Mass Mediator: The batch search option in CEU Mass Mediator (CMM v. 3.0) [91] (<https://ceumass.eps.uspceu.es/>; accessed on 16 January 2023 and 24 January 2025) was used to query the top 15 *m*/*z* values from the VIP plot, as well as other *m*/*z* values from the global screening in the XCMS workflow. Databases used were all (HMDB, LipidMaps, METLIN, KEGG, In-house, Aspergillus, FAHFA Lipids) except the MINE in silico, within a tolerance of 5-ppm excluding peptides. Adducts used for querying were [M+H]**^+^** and [M+H-H**_2_**O]**^+^**.

MetaboQuest: The mass-based database search option was used in MetaboQuest (<https://tools.omicscraft.com/aiSysMet/>, developed from MetaboSearch [90]; accessed on 07 February 2023) with the [M+H]+ adduct selected within a 5-ppm error margin, with the removal of peptides.

TurboPutative: Querying *m*/*z* values for putative compounds for MS**^1^** can generate many isomers under the same mass and molecular formula. TurboPutative [98] (<https://proteomics.cnic.es/TurboPutative/home>; accessed between 13 February 2025 to 18 February 2025) was used to concatenate the names of different compounds under the same *m*/*z* and adduct and ppm error using the Rename and Merger workflows from data generated from CEU Mass Mediator and MassTrix. For compounds with many putative hits, this was prioritized by ppm error, with three of the putative compound names used for brevity.

#### 2.5.4. Tandem Mass Spectrometry (MS^2^) Data Processing

##### Data Pre-Processing: Feature Alignment Using mzMine v. 2.5.3

To find matches for compounds fragmented using the data-dependent tandem mass spectrometry (DDA; ddMS^2^) methodology, different bioinformatics tools were used to cover as many matches as possible. The mzMine 2.5.3 analysis pipeline [99,100] was used to look for MS^1^ features corresponding to MS^2^ fragments deriving from different sample types. Data acquired from samples analyzed using ddMS^2^ were converted to the *.mzXML or *.mzML formats using the MSConvert option in Proteowizard, as mentioned previously (Figure 1). The peaks were aligned using the parameters previously published involving mass detection, chromatogram building, smoothing and deconvolution, deisotoping, alignment, and gap filling [101]. All data (for MS^1^ and MS^2^) were used and stored in *.mgf format. It should be noted that mzMine v. 2.5.3. can be used to query MS**^1^** data in KEGG within a 5-ppm error margin.

##### Metabolite Annotation Using Global Natural Products Social Molecular Networking (GNPS) via the Feature-Based Molecular Networking (FBMN) Workflow

A molecular network was created with the Feature-Based Molecular Networking (FBMN) workflow [102] on GNPS (https://gnps.ucsd.edu) (accessed on 19 December 2024; Figure 1) [103]. The mass spectrometry data were first processed with mzmine (v. 2.5.3), yielding combined spectra in *.mgf format, and the results were exported to GNPS for FBMN analysis. The data were filtered by removing all MS/MS fragment ions within +/− 17 Da of the precursor *m*/*z*. MS/MS spectra were window-filtered by choosing only the top 6 fragment ions in the +/− 50 Da window throughout the spectrum. The precursor ion mass tolerance was set to 0.02 Da, and the MS/MS fragment ion tolerance to 0.05 Da [104]. A molecular network was then created where edges were filtered to have a cosine score above 0.6 and more than 3 matched peaks [104]. Further, edges between two nodes were kept in the network if and only if each of the nodes appeared in each other’s respective top 10 most similar nodes. Finally, the maximum size of a molecular family was set to 100, and the lowest-scoring edges were removed from molecular families until the molecular family size was below this threshold. The spectra in the network were then searched against GNPS spectral libraries [103,105]. The library spectra were filtered in the same manner as the input data. All matches kept between the network spectra and library spectra were required to have a score above 0.6 and at least 3 matched peaks. The DEREPLICATOR was used to annotate MS/MS spectra [106]. The molecular networks were visualized using Cytoscape software (Supplementary Information) [107].

The molecular networking job can be publicly accessed at: https://gnps.ucsd.edu/ProteoSAFe/status.jsp?task=685a12e4b3674ae0baebafcd1fd3f3de (accessed on 19 December 2024).

##### Metabolite Annotation Using MS-DIAL (v. 5.1)

UHPLC-MS/MS raw data were processed with MS-DIAL 5.1 [108]. Automatic feature detection was performed between retention times of 0 and 15 min and for masses within 0–700 Da (i.e., *m*/*z*) for mass signal extraction in positive ionization mode. MS^1^ and MS^2^ tolerance were set to 0.01 and 0.025 Da, respectively, in profile data mode. Minimum feature height, mass slice width, and the sigma window value were all set to the default of 1000 (AU; arbitrary units), 0.1 Da, and 0.5, respectively [109]. Alignment parameters for samples were performed at 0.015 Da for mass tolerance and 0.5 min for the retention time tolerance for MS^1^. Database matches were performed with the parameters of MS^1^ and MS^2^ at 0.01 and 0.05 Da, respectively (instructions as previously published [110]). Public databases downloaded (Figure 1) and chosen for level 2 compound confirmation were as follows: MassBank and MassBank-EU [105], GNPS [103], CASMI2016 [111], ReSpect [112], BMDMS-NP [113], MetaboBASE [114], RIKEN PlaSMA authentic standards and RIKEN PlaSMA bio-MS/MS (MSI level 1, 2, 3, or 4) [115], ESI(+)-MS/MS from authentic standards, ESI(+)-MS/MS from standards + bio + in silico, and the Fiehn/Vaniya natural product library. These databases were downloaded from the MS-DIAL website (<https://systemsomicslab.github.io/compms/msdial/main.html> accessed 1 March 2023). Adducts selected were [M+H]^+^, [M+Na]^+^, [M+ACN+H]^+^, [M+H-H_2_O]^+^, [M+H-2H_2_O]^+^, [2M+H]^+^, and [M+2H]^+^.

##### In Silico Metabolite Annotation Using SIRIUS (v. 5.6.3)

SIRIUS (v. 5.6.3) was used for further molecular annotation of fragmentation patterns from ddms^2^ data [116,117,118]. The aligned MS^2^ spectra in *.mgf format that was exported from mzMine (v. 2.5.3) were imported into SIRIUS (v. 5.6.3). Additionally, converted *.mzML or *.mzMXL files for each fragmented sample (i.e., SE, TE, or LE) were imported into SIRIUS (Figure 1). The settings were as follows. Orbitrap was chosen as the instrument with the option to scope MS/MS isotopes. The MS^2^ mass deviation was set to 5 ppm, with 10 candidates chosen and 1 candidate per ion. Positive ionization adducts [M+H]^+^ were chosen, with no constraints on tree or compound timeouts. Elements existing within biological molecules (i.e., C, H, O, N, S, P) were allowed in the formula assignment. All databases were selected for querying. The default settings of Zodiac were used for SIRIUS molecular formula ranking, with the defaults for CSI—FingerID interface and CANOPUS for compound class prediction. For candidates, those with a Zodiac score greater than 90% and a similarity (SIRIUS) score greater than 75% were chosen. Not all compounds were identified by name, and within the SIRIUS module, under the structures tab, the compound with rank 1 was queried using the corresponding InChiKey in PubChem [119] if the name was not shown or if the name was unfamiliar.

##### In Silico Metabolite Annotation Using MS2Compound (v. 1.02)

The MS/MS module in the software MS2Compound (v. 1.02) [120] was used to query databases such as PlantCyc (i.e., the Plant Metabolic Network) [121], KEGG with a phytochemical compound bias [122], and PhenolExplorer [123]. For parameters, positive ion mode adduct [M+H]+ was selected with an error tolerance of 0.05 Da for parent and fragment ions, respectively, from the parameters previously published [124]. The minimum number of fragments to match was set at 4 with a CID energy set at high to correspond with the fragmentation from ddms^2^ at normalized collision energy (NCE) at 30%. Compounds with a rank of 1 and with the highest mScores were chosen as candidates (Supplementary Information). MS2Compound was also used to query MS**^1^** data for putative compounds.

#### 2.5.5. Semi-Targeted Metabolite Identification Using XCalibur 4.3

A list of putative therapeutic metabolites was assembled based on purported anti-inflammatory or antioxidant properties in plants of ethnobotanical significance (Table 3). Their identification in samples SE, TE, and LE was accomplished via semi-targeted screening for the specified metabolites in XCalibur 4.3 (proprietary software via ThermoScientific) through the identification of fragmentation patterns using full scan and ddMS^2^ data. This was performed by extracting the corresponding *m*/*z* values for each therapeutic metabolite (i.e., Table 2), and the chromatographic peak areas were manually integrated for the five biological replicates from each sample type. Fold changes for therapeutic compounds were calculated as the ratio of average peak areas of [Treatment]/[Control] (Supplementary Information), where the treatment was considered either TE or LE, and the control was the SE. Regarding metabolite annotation, these are at confidence level 3according to the Metabolomics Standards Initiative [81].

#### 2.5.6. Semi-Targeted Metabolite Identification Using HormonomicsDB (v. 1.5)

XCMS output for all files was subjected to a custom database made from querying KNApSAck [92] for compounds specific to *Euphorbia* sp. using protonated masses. Using HormonomicsDB (v. 1.5) [125] (<https://hormonomicsdb.com>; accessed on 12 January 2024), the custom database made from KNApSAcK [92] was used to query if the semi-targeted list of compounds was found in the XCMS output within a 5-ppm error. The semi-targeted compounds found are presented in the Supplementary Information.

**Table 2:** Selected therapeutic metabolites reported in the literature from other members of the *Euphorbia* sp. that were investigated using the semi-targeted approach.

Metabolite	Chemical Formula	Exact Mass	Protonated Mass [M+H]^+^	References
4-OH benzoic acid	C_7_H_6_O_3_	138.0311	139.039	[126]
Coumarin	C_9_H_6_O_2_	146.0362	147.0441	[126]
Protocatechuic acid	C_7_H_6_O_4_	154.026	155.0339	[126,127]
Caffeic acid	C_9_H_8_O_4_	180.0417	181.0495	[127]
Ferulic acid	C_10_H_10_O_4_	194.0573	195.0652	[127]
Chrysin	C_15_H_10_O_4_	254.0573	255.0652	[126]
Fisetin	C_15_H_10_O_6_	286.0472	287.055	[126]
Quercetin	C_15_H_10_O_7_	302.0421	303.0499	[15]
Ethyl linoleate	C_20_H_36_O_2_	307.2631	308.271	[128]
Myricetin	C_15_H_10_O_8_	318.037	319.0448	[126,129]
Prostratin	C_22_H_30_O_6_	390.2037	391.2115	[15,130]
Bridelionoside D	C_19_H_36_O_8_	392.2405	393.2483	[129]
Hyperoside	C_21_H_20_O_12_	464.0949	465.1028	[126]
Neriifolin	C_30_H_46_O_8_	534.3187	535.3265	[15]
Sapintoxin	C_30_H_37_NO_8_	539.2514	540.2592	[131]
Rutin	C_27_H_30_O_16_	610.1528	611.1607	[15,126,127]

#### 2.5.7. Quantification of Endogenous Phytohormones via the Isotopic Dilution Method

Phytohormone levels in different sample types derived from *E. neriifolia* (i.e., SE, TE, and LE) were reported as CK groups (aromatics, free bases, ribosides, nucleotides, glucosides, and methylthiols) and as CK types (iP-,*t*Z-, cZ-, and DZ-types), as previously published [73]. The processing and quantification of all detected phytohormones was performed using the Processing Setup and Quan Browser modules in XCalibur 4.3 [42]. To do so, all endogenous phytohormone peaks were identified using their protonated monoisotopic mass compared to the retention times of the deuterated phytohormone internal standards (IS) in full scan mode. Plant phytohormone concentrations were calculated using relative quantification through the direct comparison of analyte peak areas relative to IS using the following formula: phytohormone concentration [pmol/g FW] = (((peak area of analyte/peak area of IS) × mass of IS)/MW × 1000)/mass of sample; where FW—mass of the tissue, mass of IS = (10 ng, 20 ng or 60.1 ng) as per used in method, MW—molecular mass of CK [g] [42]. All plant sample types were analyzed in quintuplicate (*n* = 5). Based on annotation levels due to the guidelines of the Metabolomics Standards Initiative [81,82], with fragmentation patterns of all isotopically labeled standards [42], all phytohormones reported are annotated at MSI level 1. 

#### 2.5.8. Cheminformatics Using ClassyFire for Compound Class Groupings

For grouping annotated metabolites into compound class groupings, tentative metabolite names from GNPS [102], SIRIUS [116,117], and PubChem [119] were queried via the Pubchem Identifier service (<https://pubchem.ncbi.nlm.nih.gov/idexchange/idexchange.cgi>; accessed on 24 January 2025) [132], which was used to convert SMILES to InChiKeys (Figure 1; Supplementary Information). ClassyFire (<https://cfb.fiehnlab.ucdavis.edu> or http://classyfire.wishartlab.com/; accessed on 24 January 2025) [133] was used to categorize InChiKeys into compound classes. SMILES without corresponding InChiKeys and InChiKeys not found in ClassyFire were not used for the metabolite annotations. Duplicate InChiKeys were removed. InChiKeys are used for structural information instead of the SMILES format as they are standardized and have a fixed length, allowing for easy data processing [134]. Details are included in the Supplementary Information.

## 3. Results

### 3.1. General Overview

The presentation of results of this comparative investigation of the three sample types TE, LE, and SE is approached in the following manner.

(a) The broad indication of the features detected through the untargeted metabolomic approach is presented beginning with a Venn diagram of all detected features followed by an examination of the characteristics of the features in a volcano plot, which show the comparative features in SE and TE samples and SE and LE, respectively. The Venn diagram method was selected to obtain a clear representation of the features in each type of extract as well as to illustrate the relationship and similarities between the extracts. It is a simple, effective means to visualize the number of features and potential metabolites in a specific sample type or shared between two or all three types of extracts. This method does not provide information with respect to the accumulation of a feature, whether it is higher or lower in one extract type over another; thus, the volcano plot was used. The volcano plots give an overview of the number of features with higher or lower accumulation in compared samples.
(b) Subsequently, results from the semi-targeted approach employed to reveal specific putative metabolites from a pre-determined list are presented. To reiterate, the focus was to detect molecules with antioxidant and anti-inflammatory properties, assembled from literature sources, with potential benefit to asthma therapy. Identification was based on a comparison to established information in databases with respect to mass-to-charge values and fragmentation patterns. Mass spectrometric data of samples were uploaded and compared with mass spectral databases. Since the use of standards was not employed in this process, the compounds are putative of possible matches instead of definitive identifications and, thus, are reported as levels 2 to 4 [81,82].
(c) Finally, the targeted approach is presented, which identifies and quantifies the hormones in SE, TE, and LE sample types using isotopically labeled standards. The identification of compounds in this approach is at a confidence level of 1, indicating great accuracy and specificity [81,82].

### 3.2. Untargeted Metabolomics: Screening for Putative Metabolites

**Figure 2:**
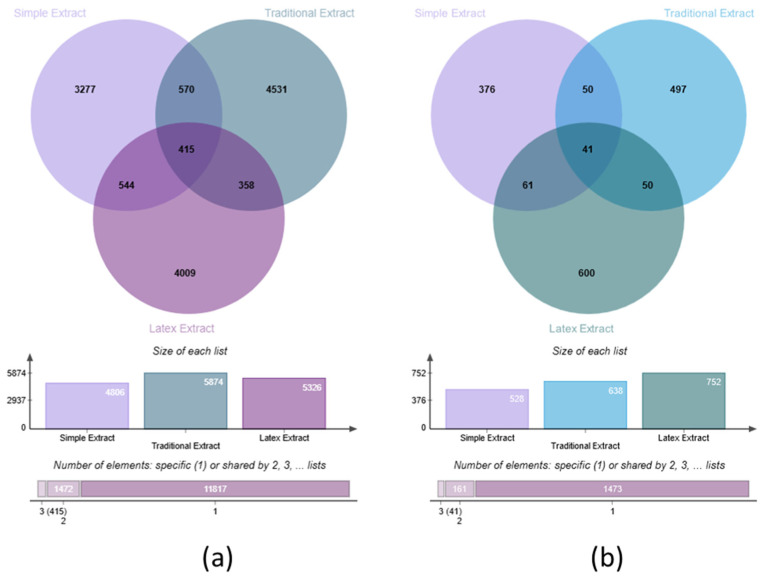
Venn diagrams of shared and unique peaks of Simple, Traditional, and Latex Extracts from LC-MS in positive ionization mode of (**a**) all features detected and (**b**) of quality peaks via manual inspection from extracted ion chromatograms (EIC) derived from XCMS Online [79,80].

As a first approach to determine the chemical potential of *E. neriifolia* sample types, a Venn diagram analysis was performed using *m*/*z* values found within the extracted ion chromatograms (EIC) range to visualize chemo-diversity. An overview of the number of features and distribution among sample types was obtained. These data are represented in Figure 2 as a triple Venn diagram showing 13,704 features detected in SE, TE, and LE samples either as unique per sample type or shared among sample types.

Less than 5000 features were detected in the SE compared to almost 6000 in TE and over 5000 in LE (Figure 2). Approximately 3% of features, or 415, are shared among all three sample types at the intersection (∩), i.e., SE ∩ TE ∩ LE, while 4.16%, 3.97%, and 2.61% are shared between SE ∩ TE only, SE ∩ LE only, and TE ∩ LE only, respectively. The lower number of shared features between TE and LE emphasizes the difference in the extract types. This trend was consistent in the same order for total shared features between SE and TE, SE and LE, and TE and LE at 7.18%, 7%, and 5.64%. These percentages represent the shared features in the entire intersections for each pair of samples. For single and unique features per sample type, TE showed more unique features uncommon to the other types with 4531, followed by LE then SE. TE presents 22% and 10% more features than SE and LE, respectively. LE shows an almost 11% higher number of features than SE.

The image displayed in Figure 2 shows a comparative analysis of three different extraction methods through two sets of Venn diagrams labeled (a) and (b). In diagram (a), which represents the larger dataset, the Simple Extract contains 4806 elements, with 3277 being unique to this method. The Traditional Extract encompasses 5874 elements, of which 4531 are exclusive to this approach. The Latex Extract holds 5326 elements, with 4009 being unique to this method. The overlapping regions show interesting intersections: 570 elements are shared between Simple and Traditional Extracts, 544 between Simple and Latex, and 358 between Traditional and Latex, while 415 elements are common across all three methods.

Venn diagram (b) presents a smaller dataset with similar relationships but different proportions. Here, the Simple Extract contains 528 elements (376 unique), the Traditional Extract has 638 elements (497 unique), and the Latex Extract includes 752 elements (600 unique). The overlapping regions, in this case, show 50 elements shared between Simple and Traditional Extracts, 61 between Simple and Latex, and 50 between Traditional and Latex, with 41 elements common to all three methods. Below each Venn diagram, bar graphs illustrate the total size of each extraction method, while the bottom bars indicate the distribution of elements that are either specific to one extract or shared among multiple methods.

**Figure 3:**
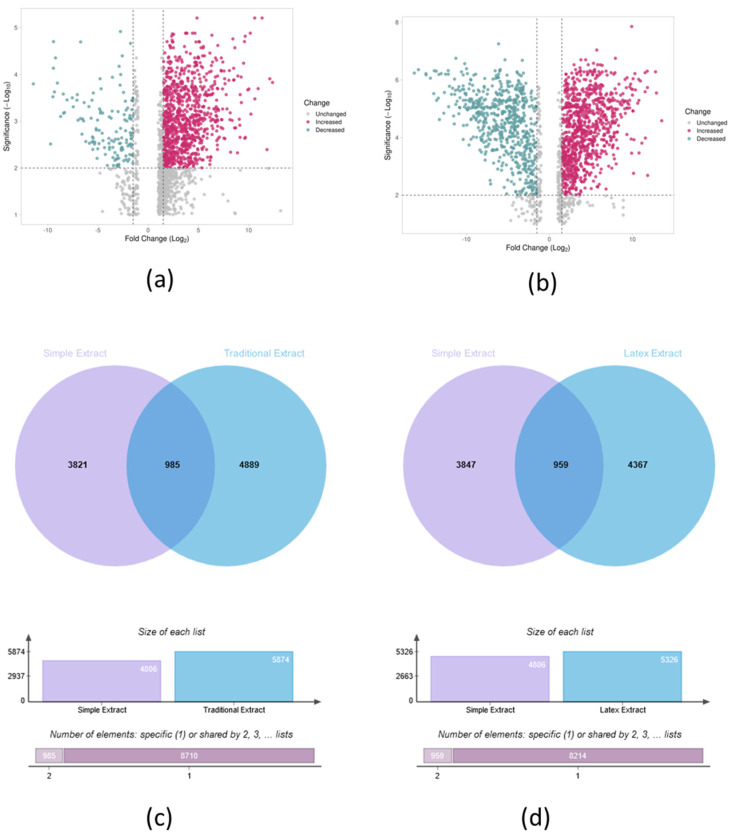
Volcano plots showing up- or downregulated *m*/*z* features (i.e., increased (pink) or decreased (cyan)) in (**a**) Simple Extract (SE: unheated; control) vs. Traditional Extract (TE: heated) and (**b**) Simple Extract (SE: unheated; control) vs. latex (LE). The threshold of fold change values is at log_2_FC > 1 or log_2_FC < −1 for features of interest. The y-axis represents −log^10^(*p*-value) between treated and control samples. Venn diagrams of features detected from LC-MS in positive ionization mode corresponding with volcano plots showing shared and unique features in (**c**) Simple Extract vs. Traditional Extract (corresponding to (**a**)) and (**d**) Simple Extract vs. latex samples (corresponding to (**b**)).

As a second approach, TE and LE were examined, with SE as control, to determine upregulated or downregulated features. Volcano plot analyses indicated 1622 features affected, 16.5% (268) in TE presented with lower abundance, otherwise termed downregulated for this purpose, and 83.5% had higher abundance or upregulation than SE. This contrasts with SE and LE in Figure 3b, where 40.3% to 59.7% of 1808 features had lower and higher abundance in LE, respectively. Abundance could be attributed to the treatment (whether heated as in TE or unheated as in SE and LE), resulting in greater or lesser extraction of metabolites. Upregulation and downregulation should be understood in the context that metabolites are observed in samples being compared and refer to the abundance of a metabolite per sample. Therefore, when one feature/metabolite is shown at elevated levels in one sample type, conversely, the same feature is lower in the control sample to which the comparison is made (TE or LE and the control SE).

The two Venn diagrams provide a comparative analysis of the extraction methods (Figure 3). The left diagram (c) juxtaposes Simple Extract with Traditional Extract, revealing 3821 unique elements in Simple Extract and 4889 in Traditional Extract, with 985 elements shared between the two. The right diagram (d) presents a similar comparison between Simple Extract and Latex Extract, indicating that Simple Extract contains 3847 unique elements while Latex Extract has 4367, sharing 959 elements between them.

**Figure 4:**
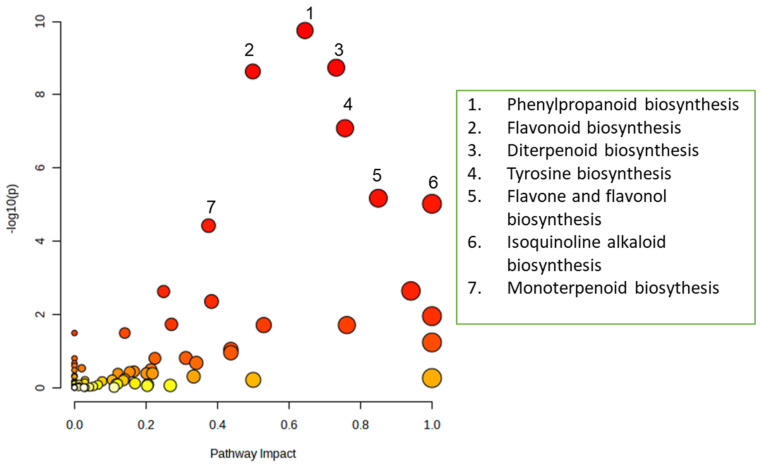
Pathway analysis of putative metabolites found using the mummichog algorithm [93] embedded within the functional analysis mode of MetaboAnalyst 5.0 [83] from all detected features from all sample types using mass to charge (*m*/*z*) values, retention time (t_R_), and *p*-values generated from alignment using XCMS Online [79,80]. Only the top 7 most significant pathways are highlighted.

The pathway enrichment analysis plot delineates several critical metabolic biosynthesis pathways and their relative significance within the biological system. The most prominent pathway identified is phenylpropanoid biosynthesis, which exhibits the highest statistical significance, with a −log^10^(*p*) value of approximately 10 and a substantial pathway impact of 0.6. Trailing closely are flavonoid biosynthesis and diterpenoid biosynthesis, both of which demonstrate high significance levels, with −log^10^(*p*) values ranging from 8 to 9. Tyrosine biosynthesis also reveals notable significance, with a −log10(*p*) value of approximately 7, whereas flavone and flavonol biosynthesis and isoquinoline alkaloid biosynthesis reflect moderate significance levels, with −log^10^(*p*) values around 5. Monoterpenoid biosynthesis, while exhibiting lower significance, remains noteworthy among the identified pathways. The visualization employs a color gradient transitioning from yellow to red, with red denoting higher significance and the size of the circles corresponding to the importance of each pathway. A majority of the pathways cluster in the lower left corner with yellow coloring, suggesting that these pathways possess relatively lower significance and impact compared to the seven highlighted pathways that are distinguished in the analysis (Figure 4).

**Figure 5:**
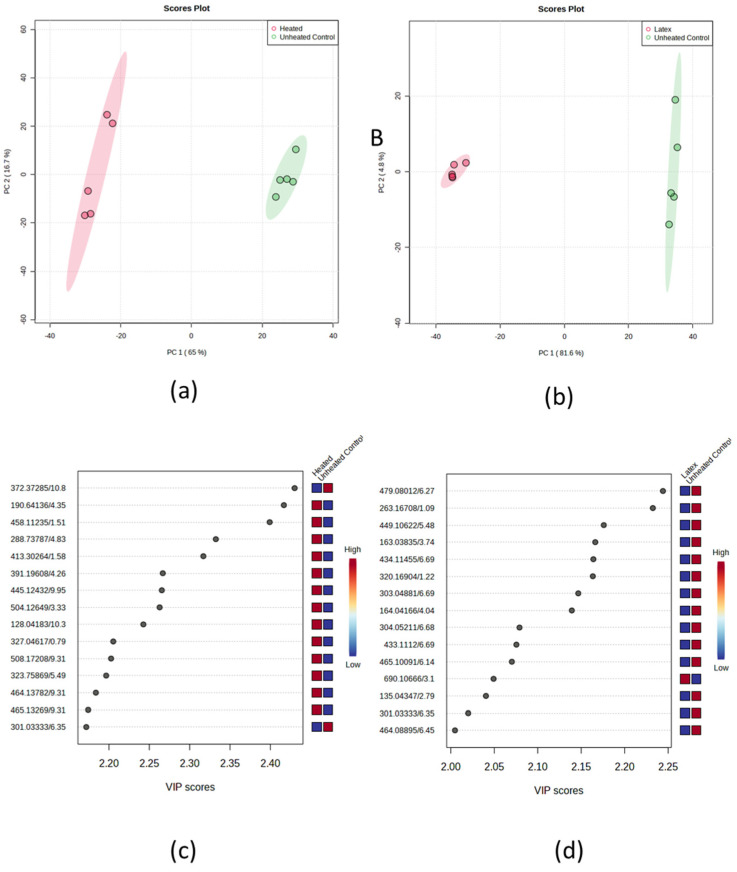
PCA and Variable Importance of Projection (VIP) generated by MetaboAnalyst 5.0 [83] comparing traditional (heated) extract samples to the simple (unheated) extracts (**a**,**c**), and latex to the simple (unheated) extract (**b**,**d**). PCA gives an overview of the separation, while VIP plots generated from PLS-DA data show which features cause separation with sample types.

From this point forward, the untargeted approach was narrowed to primarily focus on screening specific features in sample types in relation to asthma therapy. The results in (Figure 5) present a comprehensive statistical analysis comparing various experimental conditions through Principal Component Analysis (PCA) and Variable Importance in Projection (VIP) scores. The upper portion of the image contains two PCA score plots labeled (a) and (b). In plot (a), a clear separation is observable between heated and unheated control samples along Principal Component 1, which accounts for 65% of the total variance. The samples form distinct clusters, with the heated samples located on the left side and the unheated controls on the right, surrounded by confidence ellipses. Plot (b) illustrates a similar comparison between latex and unheated control samples, with Principal Component 1 explaining an even larger portion of the variance at 81.6%. The separation between these groups is equally distinct, with latex samples positioned on the left and control samples on the right of the plot. The confidence ellipses around each group indicate minimal overlap between the experimental conditions. The lower half of the image displays VIP score analyses in plots (c) and (d), which identify the most significant variables contributing to the separation observed in the PCA plots. Plot (c) presents VIP scores for the heated vs. control comparison, with numerical identifiers for each variable on the left axis and their corresponding VIP scores plotted horizontally. Adjacent to these scores is a heatmap illustrating the relative intensities of each variable between conditions, employing a color gradient from blue (low) to red (high). Plot (d) offers a similar analysis for the latex vs. control comparison, featuring its own set of significant variables and corresponding heatmap visualization. All variables presented exhibit VIP scores exceeding 2.0, indicating their substantial contribution to distinguishing between the experimental conditions.

The differences between the sample types determined as major features responsible for divergence by the VIP were checked in MetaboQuest for putative matches using the *m*/*z* values in the VIP. One *m*/*z* value that occurred at 301.03333 on the VIP was shown to be involved in the separation in both plots. Putative matches for this value were returned as pseudopurpurin or emodic acid, both anthraquinones with formula C_15_H_8_O_7_ and similar exact masses at 300.027 (Supplementary Information).

The features in the VIP were scrutinized in the annotated single-class metabolite list to check their extracted ion chromatographs (EICs). Forty percent of the features in the VIP for both groups, TE vs. SE and LE vs. SE, were contained in the top two thousand features. Therefore, more features responsible for main differences were found to occur outside of the main peak features, i.e., features for which electron ion chromatograms were available. Most of these features were further unmatched in the databases and, therefore, returned no putative metabolites occurring at the *m*/*z* values or were unrelated to inflammatory or antioxidant properties. The TE vs. SE VIP also revealed possible putative compounds with properties for cardiac and bone tissue consistent with other reported uses of *E. neriifolia*.

**Figure 6:**
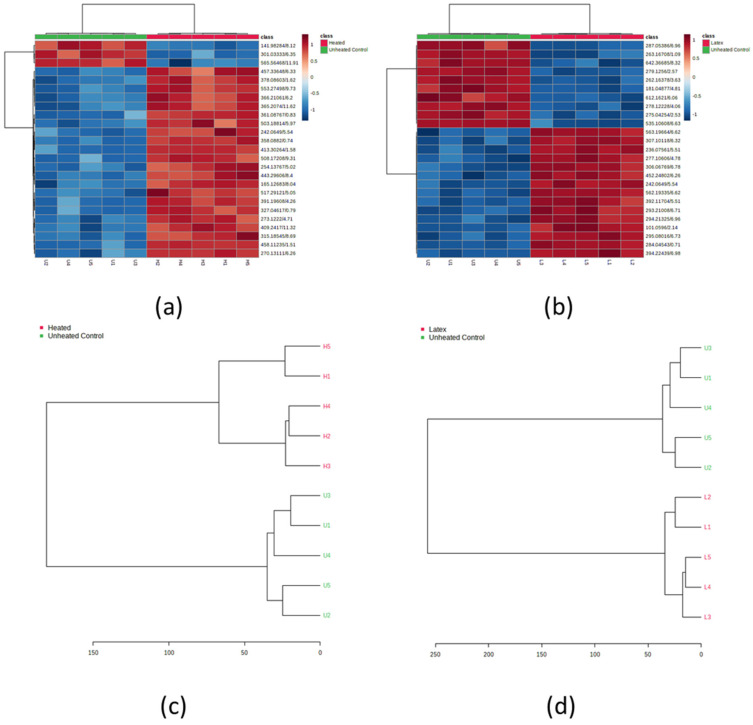
Heatmap and dendrogram analyses showing clear separation of treatments from the control. (**a**,**c**) show heatmap analysis for the top 25 features and the dendrogram generated using Euclidean distance with the Ward algorithm for heated treatments when compared to the unheated control. (**b**,**d**) pertain to the latex when compared to the unheated control.

The results in Figure 6 present a comprehensive bioinformatics analysis through four distinct panels labeled (a) through (d). The top panels (a) and (b) showcase heatmap visualizations with hierarchical clustering, where expression patterns are represented by a color gradient from blue to red (Figure 6). Each heatmap contains multiple rows corresponding to different identifiers, with samples grouped into two conditions: “Heated” (shown in red) and “Unheated Control” (shown in green). The color intensity scale ranges from −1 to 1, suggesting normalized expression values or correlation coefficients.

The bottom panels (c) and (d) display phylogenetic trees or dendrograms that illustrate the hierarchical relationships between samples. Panel (c) shows a dendrogram with ten samples labeled from H1-H5 (Traditional Extract; heated) and U1-U5 (Simple Extract; unheated control), with a scale bar extending from 0 to 150. Panel (d) presents a similar hierarchical structure but with fewer samples and a scale bar ranging from 0 to 250. In both dendrograms, the samples clearly cluster into two distinct groups, with traditional heated leaf samples (labeled with H) forming one cluster and simple leaf extracts (unheated control; labeled with U) forming another, indicating strong experimental condition-specific patterns in the underlying data.

The overall visualization effectively demonstrates the clear separation between traditional and simple leaf extracts, as well as latex and simple leaf extracts, suggesting significant differences in the molecular profiles or expression patterns between these two experimental groups. The hierarchical clustering in both the heatmaps and dendrograms reinforces this distinction, showing consistent patterns across different analytical approaches.

### 3.3. Semi-Targeted Screening of Therapeutic Metabolites from Literature

This more focused method was employed following the broad untargeted approach. Here, metabolites with inflammatory and antioxidant properties were assembled from the literature on medicinal plants for investigation rather than researching the plethora of putative matches returned from the global screen to determine if these were the main characteristics. The term semi-targeted is used since it involves targeted identification of specific metabolites from the untargeted global screen or full scan data instead of investigating what shows up in its entirety. Additionally, manual inspection of individual extracted ion chromatograms (EICs) was performed in each sample type to ensure acceptable quality *m*/*z* peaks, reducing the putative candidate pool from the original 13,704 to 1675, or roughly 12% (Figure 2). This reduced feature set was used for the detection and identification of secondary metabolites with known characteristic properties in asthma therapy in a semi-untargeted approach, as previously indicated. Confirmation of the results was accomplished at an annotation/confidence level of 4 [81]. A greater number of metabolites of interest were detected in TE and SE than in LE.

**Figure 7:**
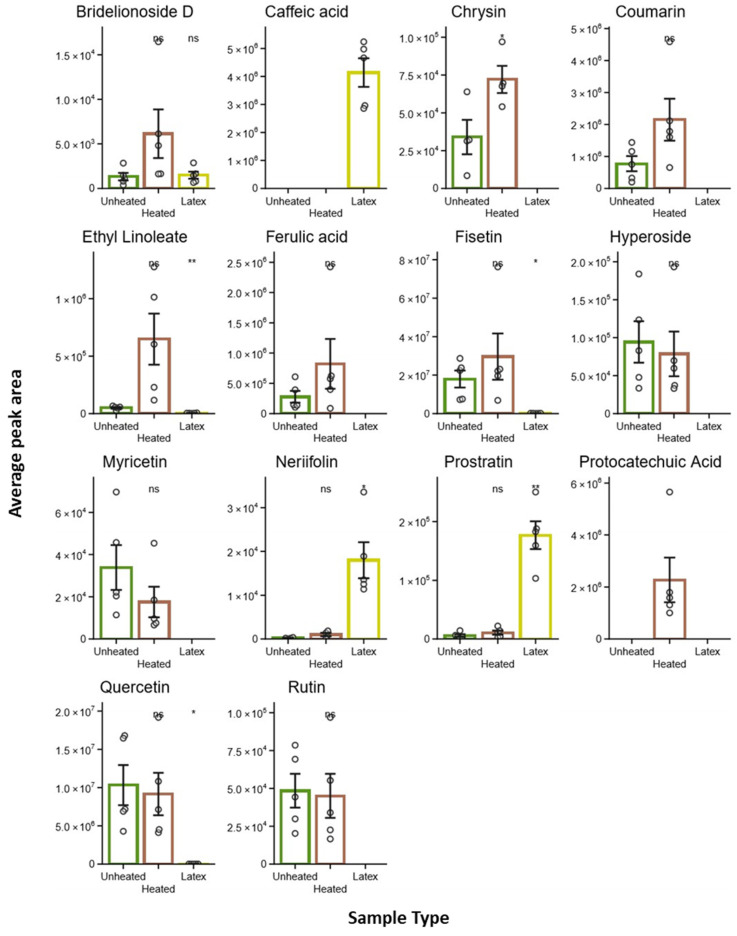
Bar plots of therapeutic metabolites showing average peak areas according to sample type (n = 5). The star symbol (*) signifies statistical difference between the referenced control (i.e., Simple Extract (unheated)) and the treatment investigated (i.e., Traditional Extract (heated) or Latex Extract samples). The term “ns” means not significant. * for *p*-value < 0.05, and ** for *p*-values < 0.01. Bar plots were generated using Metabolite Autoplotter [135].

The results demonstrated in Table 3 and Figure 7 present a comprehensive analysis of 14 different chemical compounds measured under three distinct conditions: unheated, heated, and latex treatments. The data are displayed as bar graphs showing average peak areas with error bars indicating measurement variability. Several notable patterns emerge from this analysis. Some compounds, such as caffeic acid, show exclusive or dramatically increased presence in latex samples, while ethyl linoleate demonstrates a significant increase in heated conditions, marked by double asterisks indicating high statistical significance (*p* < 0.01). Chrysin and coumarin both exhibit elevated levels in heated and latex conditions compared to their unheated state.

**Table 3:** Metabolites with antioxidant and anti-inflammatory properties from a list assembled from the literature (Table 3) to devise a more focused identification of compounds from the broad global screen in XCalibur 4.3. Annotations here are at level 3, as they are based on extracting peak areas from the protonated masses, as determined by the Metabolites Standards Initiative [81]. Peak areas for compounds were present in at least 3 biological replicates.

Metabolite	Chemical Formula	Exact Mass	[M+H]^+^	Detected in Sample
Coumarin	C_9_H_6_O_2_	146.0362	147.0441	SE, TE
Protocatechuic acid	C_7_H_6_O_4_	154.026	155.0339	TE
Caffeic acid	C_9_H_8_O_4_	180.0417	181.0495	LE
Ferulic acid	C_10_H_10_O_4_	194.0573	195.0652	SE, TE
Chrysin	C_15_H_10_O_4_	254.0573	255.0652	SE, TE
Fisetin	C_15_H_10_O_6_	286.0472	287.055	ALL
Quercetin	C_15_H_10_O_7_	302.0421	303.0499	ALL
Ethyl linoleate	C_20_H_36_O_2_	307.2631	308.271	ALL
Myricetin	C_15_H_10_O_8_	318.037	319.0448	SE, TE
Prostratin	C_22_H_30_O_6_	390.2037	391.2115	ALL
Bridelionoside D	C_19_H_36_O_8_	392.2405	393.2483	ALL
Hyperoside	C_21_H_20_O_12_	464.0949	465.1028	SE, TE
Neriifolin	C_30_H_46_O_8_	534.3187	535.3265	ALL
Rutin	C_27_H_30_O_16_	610.1528	611.1607	SE, TE

Interestingly, some compounds, like protocatechuic acid, appear only in heated samples, while others, such as prostratin and nerifolin, show higher concentrations in latex samples. Fisetin displays a statistically significant increase in heated samples (marked with a single asterisk, *p* < 0.05), and quercetin shows variations across all three conditions. In contrast, several compounds, including bridelionoside D, myricetin, and rutin, demonstrate no statistically significant changes between conditions, as indicated by the ‘ns’ notation.

The y-axis scales vary considerably between compounds, ranging from 10^4^ to 10^7^ peak area units, suggesting wide variations in compound concentrations or detection sensitivities. The experimental design appears robust, with multiple replicates for each condition, as shown by the individual data points overlaid on the bar graphs, providing confidence in the observed trends and statistical analyses presented.

### 3.4. Using ClassyFire for Metabolite Annotation

**Figure 8:**
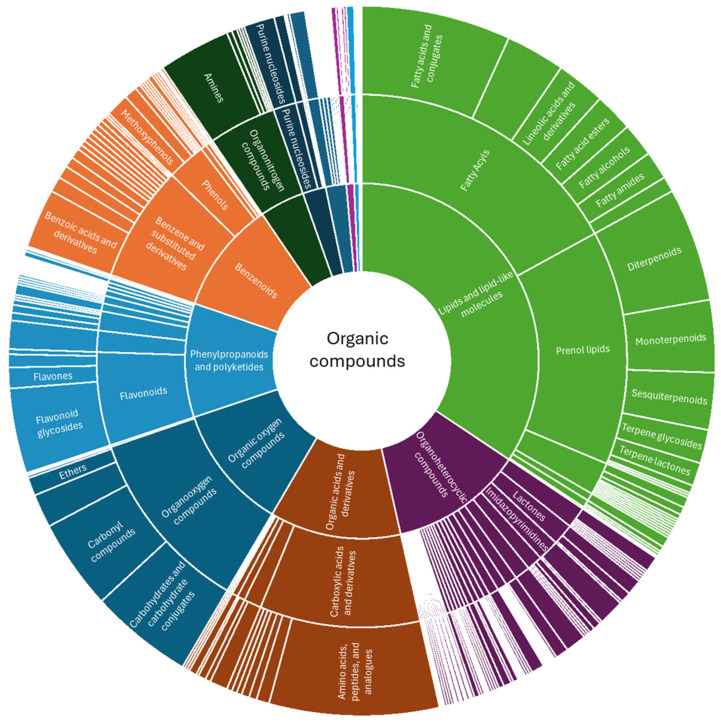
Sunburst plot of the different class levels of compounds annotated by ClassyFire from positive ionization mode from annotated metabolites from levels 1 to 3 in *Euphorbia neriifolia*. These compounds comprise untargeted, targeted, and semi-targeted approaches. ClassyFire summarizes the compounds according to the kingdom, superclass, class, and subclass levels. Superclasses of benzenoids, lipids and lipid-like molecules, organic acids and derivatives, organoheterocyclic compounds, phenylpropanoid and polyketides, and organic oxygen compounds dominate in contributions of annotated compounds.

A total of 850 compounds were annotated using a combination of bioinformatics tools such as MS-DIAL, GNPS, MS2Compound, SIRIUS, HormonomicsDB via a custom database via KNApSAcK, and semi-targeted compounds from literature, in addition to phytohormone data. These compounds ranged in levels from 1 to 4 in metabolite confirmation based on the Metabolomics Standards Initiative (MSI). In looking at the 13 superclasses of compounds annotated, lipids and lipid-like molecules (34.35%), benzenoids (10.24%), organic acids and derivatives (12%), organoheterocyclic compounds (12%), and phenylpropanoids and polyketides (10.35%) constituted the majority (Figure 8).

**Figure 9:**
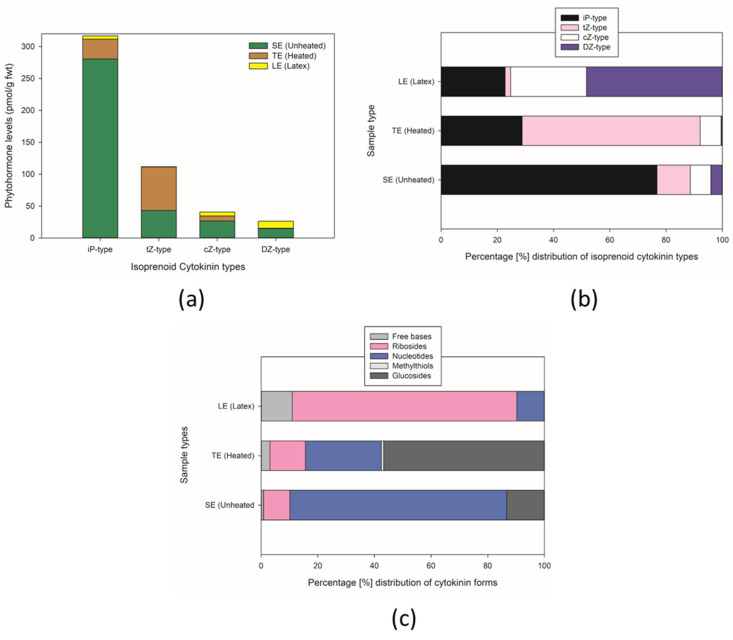
Stacked bar plots according to sample type of (**a**) isoprenoid cytokinin levels, (**b**) percentage distribution of phytohormone levels, and (**c**) percentage distribution according to cytokinin forms. Error bars in (**a**) were included in the graph but were not visible due to scaling or graphical constraints.

Further examination of lipids and lipid-like molecules, the classes of fatty acyls and prenol lipids dominated in abundance (49.7 and 41.4%, respectively). Terpenoids in different forms fall under prenol lipids using the ClassyFire ontology (Figure 9). In investigating phenylpropanoids and polyketides, the classes of flavonoids, coumarin and deivatives, and cinnamic acids and derivatives constituted the majority of compounds (54.5, 11.4, and 11.4%, respectively). Under benzenoids, benzene and substituted derivatives and phenols constituted the majority of compounds (69% and 23%, respectively). Lastly, under organic acids and derivatives, carboxylic acids and derivatives dominated in contribution (79%), followed by hydroxy acids and derivatives to a lesser degree (8.6%).

Metabolite annotation presented reveals a diversity of metabolites, showing the immense potential of *E. neriifolia*.

### 3.5. Targeted Metabolomics: Screening of Cytokinin (CK) and Acidic Type Phytohormones

There were observable variations in the proportion and amount of the six cytokinin fractions in the different sample types (Table 4). Almost twice the number of cytokinins were detected in SE (13) compared to LE (7). The highest number occurred in TE, with 22. In SE, no aromatics (AR), 2 FBs, all RBs, 3 NTs, 4 GLUCs, and 3 acidic hormones were detected. Notably, aromatic CKs were detected only in heated samples together with 3 FBs, all RBs, NTs, GLUCs, and 1 MeS but no acidic hormones. Latex Extracts showed the fewest with 1 FB, all RBs, 2 NTs, and 1 acidic hormone (Table 4).

Of the six possible cytokinin classes, four were detected in the SE compared to TE, where all six were detected and only three in LE. SE contained the highest concentration of total CKs, followed by TE, while LE was much lower than both. The FB form of CKs was notably low relative to other CK forms in all sample types. MeS form in trace amounts and AR-CKs were detected in TE, unlike other sample types lacking them, while LE was additionally deficient in GLUCs (Table 4; Figure 9c).

The differences extended to the percentage composition of the specific fractions among the sample types. SE comprised 76% NTs, 14% GLUCs, 9.2% RBs, and 0.8% FBs. In TE, NT forms (29%), GLUCs (44.5%), RBs (5.8%), FB (1.4%), with AR being 42.68% and a trace amount of MeS (Figure 9). NTs are prone to degradation into RBs [136], which could possibly explain the significant change in NTs between SE and TE from 76% to 29%. The RB level in TE was still lower than SE, but the ratio of RBs to NTs in SE and TE increased from 0.12 to 0.2, respectively. Therefore, differences in CK levels could partially be a function of degradation and/or aggressive extraction of components in TE that were otherwise available in SE. Notably higher levels of R-O-GLUCs were present in TE. This was probably due to interconversion via glycosylation of free base to O-GLUCs to a lesser extent, but more likely due to the extraction method. LE samples, on the other hand, despite having lower total CKs compared to SE and LE, showed a distinct difference with a higher percentage composition of the active CK forms in plants at 77.5% RBs and 10.7% FBs (Figure 9c). 

NT CK forms were detected at higher levels in SE and TE samples compared to LE, while LE samples showed more FB than NT. SE exhibited greater levels of iPNT and *t*ZNT than TE, but TE showed more cZNT and DZNT. Higher levels of cZNT at 45.13 pmol/g fwt in TE compared to 8 and 1.92 pmol/g fwt in SE and LE, respectively (Table 4), could indicate other potential applications. The trend iPR and iP for SE and TE are both approximately in proportions 1:8 within sample types. While DZ was undetected, the corresponding ribosides were present. Overall, a higher number of CK phytohormones at varying levels were present in TE than SE and LE (Figure 9). LE, often referred to as sap or milky sap [137], contained higher levels of DZR and cZR compared to LE and TE. The amount of these two ribosides was also higher than both iPR and tZR within the same LE sample.

Generally, DZ-type cytokinins were detected as RB and NT forms in samples. Patterns of RB detection levels varied among the samples, as SE showed higher levels of iPR > DZR > *t*ZR > cZR compared to TE with iPR > cZR > *t*ZR > DZR and LE with DZR > cZR > iPR > *t*ZR. This is interesting, as our hypothesis predicted higher levels of active phytohormones in TE with greater potential in therapy. However, the dose-dependent activity of the molecules is also a consideration in pharmacological response which would necessitate further investigation as evidence with respect to hormones and asthma is unavailable.

The results in Figure 9 present a comprehensive analysis of cytokinin distribution across three different sample types: SE (unheated), TE (heated), and LE (latex). Phytohormone levels in graph (a), there is a striking dominance of iP-type cytokinins, particularly in SE (unheated) samples, reaching concentrations of approximately 300 pmol/g FW. The other cytokinin types (*t*Z-type, cZ-type, and DZ-type) show considerably lower concentrations, generally below 100 pmol/g FW.

The percentage distribution shown in graph (b) reveals distinct patterns across the sample types. SE (unheated) samples are characterized by a high proportion of iP-type cytokinins, accounting for roughly 80% of the total. In contrast, TE (heated) samples display a more balanced distribution between iP-type and *t*Z-type cytokinins. LE (latex) samples exhibit a unique profile with a substantial presence of DZ-type cytokinins, distinguishing them from the other sample conditions.

Figure 9 further elaborates on the distribution of cytokinin forms, showing remarkable differences between sample types. SE (unheated) samples contain predominantly nucleotides and methylthiols, while TE (heated) samples are rich in nucleotides and glucosides. LE (latex) samples display a distinct profile dominated by ribosides, with smaller proportions of free bases and nucleotides. These variations in both cytokinin types and forms suggest significant biochemical differences between the sample conditions, potentially reflecting different physiological states or responses to treatment.

## 4. Discussion

### 4.1. General Overview

*Euphorbia neriifolia* is a species in the Euphorbia genus from the family Euphorbiaceae used in Indigenous cultures. Extracts from heated leaves of *E. neriifolia* are traditionally given to babies and children in Guyana to treat wheezing in asthma, while the latex is indicated for rashes [16], but the chemistry of the local species is unknown and unexplored. While latex is used for asthma in Indigenous cultures in India [15], this has not been a reported practice in Guyana. Furthermore, information on the heated leaf extract is sparse globally. Therefore, the three sample types, traditional heated extract (referred to as either heated or TE), latex extract (LE), and simple leaf extract (SE) or unheated, were investigated comparatively within the context of asthma therapy, with SE used as a control. TEs were prepared in a manner to mimic the traditional method of preparation, which involved using the liquid collected from squeezing heated leaves. Extracts of SE, TE, and LE were subjected to phytochemical and phytohormone extraction procedures as previously described. These yielded suitable fractions for metabolites, acidic hormones, and cytokinins to be separated and detected through UHPLC-MS/MS to determine their presence in each sample type and for further identification of putative metabolites.

A broad survey using untargeted metabolomics of the features of two comparison groups, heated (TE) vs. control (SE) and latex (LE) vs. control (SE), was performed, allowing for identification at confidence level 4 [81,82]. For increased specificity, a semi-targeted list of metabolites compiled from the literature was used to identify features based on comparison to databases using *m*/*z* values and fragmentation patterns, raising the confidence to levels 2 and 3 for certain metabolites and leading to putative metabolite identification. Finally, complete identification of hormones via a targeted approach with confidence level 1 was achieved with the use of isotopically labeled standards [81,82].

Untargeted metabolomics provided a broad overview of the contents of different sample types of *E. neriifolia*. This non-discriminatory approach captured all observable features within the sample types, revealing the overall remarkable potential of the species. Features seen at this level included all putative phytochemical and phytohormone metabolites using untargeted and semi-targeted approaches, ranging from annotation levels 4 to 5 based on the Metabolomics Standards Initiative (MSI). The targeted approach can be prohibitive due to the high costs of isotopically labeled standards, and as such, only phytohormones were determined at metabolite annotation/confidence level 1.

Despite multiple studies reporting on the efficacy of phytohormones in treating human disease states [49,55,66], phytohormone profiling in species of the *Euphorbia* genus is yet to be further explored in relation to therapeutics. The functions of cytokinins remain elusive, but the class attracts attention, particularly due to the role of kinetin and zeatin and their therapeutic potential [138]. Generally, studies on secondary metabolites to treat medical conditions appear to overshadow the exploration of the therapeutic potential of phytohormones, evident in the dearth of research on the *Euphorbia* species. Since secondary metabolites have been studied in various species of Euphorbia, though less in *E. neriifolia*, here, the phytohormone profiles for three sample types of *E. neriifolia* are presented for the first time. Phytohormones are gaining research traction, primarily phytohormones like abscisic acid, salicylic acid, auxins, gibberellins, and jasmonates [63,139].

### 4.2. Untargeted Metabolomics Reveals an Abundance of Features from All E. neriifolia Sample Types

The vast number of features shown in all sample types together attest to the significant potential of *E. neriifolia* and its implication for multiple conditions, including malaria, syphilis, gonorrhea, hypertension, diabetes, hyperlipidemia, blood disorders, and psychological conditions among multiple traditional uses globally [20,140]. The pharmaceutical value of *E. neriifolia* is further supported by recent use in patients hospitalized with COVID-19 [69], where patients treated with *E. neriifolia* leaves as an adjunct to standard of care improved faster than patients given a standard of care regimen only. Although that study cannot be deemed statistically significant due to the small number of participants, the findings were nevertheless encouraging. The method of use employed in the study would be similar to using SE. Therefore, while findings of more features in one sample type can be taken as greater potential of one over the other as there are possibly more metabolites with potential activity, taken in isolation may not necessarily be definitive in terms of pharmacologically relevant activity. Each sample type could have different applicable roles in therapy. The difference in the chemical composition of latex compared to TE and SE was expected, but the high number of features detected was surprising, considering latex is exuded with specific functionality in plant defense from the laciferous system [26,137]. The possibility exists that this attribute is responsible for latex being deemed the most valuable component of the *Euphorbia* species [141]. However, with the reported toxicity, commercial use as paints and rubber is probably more likely responsible for this categorization [141,142] since no literature seen to date alluded to this extensive number of features in this sample type previously. The possibility exists for employing LE in treatments different than other sample types, resulting from the chemical uniqueness of LE. Although TE samples presented a higher number of features, it was initially expected to possess significantly more features than LE.

In observing LE features, most of what is known about latex results from research on few species related to rubber production like the “rubber tree” *Hevea brasiliensis* from which natural rubber is made and *E. characias* from which many diterpene compounds were characterized [142]. Despite hypotheses on possible functions based on the presence of concentrated defense substances, the specific functions of laciferous systems and latex remain unknown [137]. Proteomics formed a major research component of *H. brasiliensis* due to allergic sensitization that occurs with rubber/latex use in humans [143,144] and continues to enjoy the spotlight with *E. characias*. Pathogenesis-related (PR) proteins were the most identified proteins in latex [137,145]. Although enzymes, carbohydrates, and minerals continue to be studied in latex [146], the continued focus on these constituents results in a lack of a comprehensive broader range of compounds in latices.

Secondary metabolites like flavonoids, alkaloids, and terpenoids are credited with being more biologically active and dominant constituents of *Euphorbia* [141,147], thus the next step was to investigate major pathways expressed to determine if these secondary metabolites were consistent with the literature. Features from XCMS Online, including *m*/*z*, retention time, and *p*-values, were used to determine metabolites in the Kyoto Encyclopedia of Genes and Genomes (KEGG) [95] database with the *Arabidopsis thaliana* reference metabolome. Annotated compounds were screened and mapped via the pathway enrichment module in MetaboAnalyst 5.0. This was performed to reveal the major metabolic pathways in which the detected features are involved and to confirm the presence of the principal characteristic constituents of *Euphorbia.* The pathway analysis is shown in Figure 4. The finding is consistent with the literature as features involved in the pathways of highest significance belong to classes of terpenes and flavonoids. Additionally, flavonoid formation is perpetuated by the phenylpropanoid pathway, which is shown as the most significant [148].

It warrants attention that up to this point, the untargeted approaches previously shown using the Venn diagram, volcano plot, and pathway analyses produced an overall view of *E. neriifolia* constituents. The process was non-selective since the global screen used all the data returned from XCMS Online with regards to the *m*/*z* values and, therefore, includes all metabolites irrespective of categorization as phytochemicals or phytohormones. Despite some shared features shown in the Venn diagram, *E. neriifolia* sample types were revealed to be distinctly different from each other. This can be adequately seen in the Principal Component Analysis (PCA) (Figure 5). The PCA transforms original variables into a new set of uncorrelated variables called principal components (PCs). Each PC is a linear combination of the original variables, and the first few typically account for most of the variation in the data. This was used to visualize the overall structure and cluster patterns in the data, identify outliers, and detect trends in the sample types [149]. The plots show comparisons of TE vs. SE and LE vs. SE, respectively.

The PCA, together with Partial Least Squares Discriminant Analysis (PLS-DA)—a multivariate statistical method used in metabolomics studies to analyze and classify data based on differences in pre-defined groups—were used to identify variables or features responsible for the separation between the sample types. Fifteen top features were determined most responsible for the divergence of samples of TE vs. SE and LE vs. TE by the Variance in Importance Projection (VIP) emanating from the PLS-DA. Eight were identified at a confidence level of 5.

Due to extended annotation (Supplementary Information), only select metabolites with therapeutic potential will be discussed. Anthraquinones are noted for their wide range of activities, including antioxidant and anti-inflammatory properties [150]. Emodic acid or emodin displayed suppressant effects on immunoglobulin E (IgE)-mediated anaphylaxis [151]. Suppression of this cytokine is beneficial to asthma and other allergic diseases. Emodin is also known for its laxative effects, and the literature indicates the use of *E. neriifolia* as a laxative [15].

The *m*/*z* values from the total screen were then subjected to database searches in MetaboQuest, CEU Mass Mediator, Pathos, and MS2Compound. Characteristic phytoconstituents of *Euphorbia* were identified in *E. neriifolia,* which included afzelin and kaempferol, namely kaempferol-3-O-rutinoside and kaempferol-3-O-α-rhamnoside. These compounds were expected in this plant, as confirmed in previous studies [15,19], and were identified at a confidence level 2 with the matching of *m*/*z* values and fragmentation pattern through databases. These compounds were reported to exhibit anti-inflammatory properties. Terpenes like linalool and 1,8 cineole (Supplementary Information) were indicated to possess anti-inflammatory properties with a steroid-saving effect for asthma [152]. 1,8 cineole reduced various cytokine levels of IL-1B, IL-4, IL-6 IL-13, and IL-17, as well as TNF-α and PGE2 [153]. Analogs of linalool and 1,8-cineole (i.e., dehydrolinalool and dehydro-1,8-cineole) were shown in the global screen at *m*/*z* 153.1267, and (3R)-linalool and (3S)-linalool at *m*/*z* 155.1424 but were not confirmed to level 2 (Supplementary Information).

Although enzymes and proteins could possibly be identified in the untargeted approach relating to asthma therapy, this area could be a future consideration, as evidence indicates a large amount of these constituents exist in latex [145]. Secondary metabolites noted in LE include general classes: terpenes, alkaloids, proteins, enzymes, phenolics, and cardenolides. Triterpenoids, namely cycloartenol, lupeol, and lanosterol, are reported to have higher concentrations in latex than other tissues. These latex constituents are representative of multiple families [145,154] but may differ according to species. Lupeol and lanosterol were detected at an annotation level of 3 via MS2Compound MS^1^ search (Supplementary Information).

Four other metabolites, namely rutin, luteolin, chlorogenin, and isatin, were confirmed at a confidence level of 2 (Supplementary Information). Eudesmin was detected only in the traditional extracts at the [M+Na]^+^ adduct. Eudesmin, a lignan shown to possess neuroprotective properties with application in Alzheimer’s disease, also indicates antibacterial and anti-inflammatory activity [155].

Isatin, a versatile indole derivative from plants, has been shown to possess antiallergic properties in addition to cytotoxic, antimalarial, antiviral, and antimicrobial pharmacological activity [156,157]. It is also a metabolite of the neurotransmitter serotonin and has been shown to positively affect dopamine levels in conjunction with antiparkinsonian agents [158]. Given the traditional use of *E. neriifolia* for brain stimulation, isatin may be a contributor. Luteolin and kaempferol-3-O-α- rhamnoside were both found in SE and TE. Isatin was not detected in TE but was the only compound in this group seen in LE. All compounds were detected in SE except a purine nucleoside, N**^6^**-threonylcarbamoyladenosine, which was only seen in TE. Although it is not unexpected, as this t^6^A nucleoside is conserved in tRNAs across kingdoms, its presence only in the heated sample warrants further exploration. It was noted as a potential biomarker for COVID-19 disease [159]. This finding was interesting due to the similarity in respiratory distress between COVID-19 and asthma and the results of detected cytokinins that follow.

Selected compounds from the global screen that showed up in multiple databases were checked in the literature, and those having anti-inflammatory properties detected at a confidence level of 4 are shown in Supplementary Information. Roseoside or vomilfoliol 9-O-B-D glucopyranoside has been noted in multiple databases and has been detected in *E. heteradena* [160]. No studies were found to show its presence in *E. neriifolia.* Its actions, however, are indicated as anti-inflammatory in a study on *Chaenomeles speciosa* [161]. The phorbol ester 12-deoxyphorbol-13-angelate (Supplementary Information) was detected only in the latex (LE) sample, which is congruent with other studies for the species for the presence of phorbol esters [162]. However, no studies were found that indicated its presence in *E. neriifolia*. The chemical formula C_35_H_34_O_6_ is the same as for ingenol mebutate, a compound known for its use in actinic keratoses, and both are tetracylic compounds with similar structures [4]. These compounds were identified as being the same [163]. No study has reported its presence in this species to date. Overall, more compounds in the untargeted global screen were detected in SE than in the other sample types.

### 4.3. Semi-Targeted Screening—Searching for the Presence of Therapeutic Metabolites

Fourteen compounds from the assembled list for semi-targeted identification were detected (Figure 7). Protocatechuic acid [164], an ingredient in grapes, onions, and green tea that attenuates airway inflammation [165], was detected in only TE, and caffeic acid with DNA damage protection and antioxidant properties [166] was identified only in LE. Both compounds are phenolic acids with a carboxylic group moiety [164]. Supplementation with protocatechuic acid in piglets decreased serum levels of TNF-α, IL2, and IL-6 levels [167]. The reduction in IL-6 in humans could be a potential mechanism of action in improving asthma symptoms. This shows that different compounds with similar functions are expressed in the sample types. Although neither of these was present in the SE, ferulic acid, belonging to the same group [167], was detected in that sample type and has been shown to prevent airway remodeling by inhibiting TH2 cytokines [168].

Latex, deemed to have pro-inflammatory and tumor-promoting activity and toxicity associated with phorboid compounds with ingenane, tigliane, and daphnane diterpene derivatives [169], shows fewer compounds with anti-inflammatory properties from the assembled list. The presence of neriifolin was not surprising in LE as it is consistent with the literature [170]. In contrast, prostratin, a tigliane-type phorbol ester isolated from *E. fischeriana* and assessed for its anti-HIV properties [171,172], was unexpected in TE and SE samples of sweet aloes even at low doses due to phorbol toxicity.

### 4.4. Targeted Screening—Quantification of Cytokinin and Acidic Phytohormones in E. neriifolia

The perceived value of phytohormones in therapy has been increasing, and their roles as nutraceuticals are under investigation [37,61,173]. The Traditional (TE), Latex (LE), and Simple Extracts (SE) were investigated for cytokinins. Samples spiked with isotopically labeled standards (IS) allowed for quantification based on the comparison of relative peak areas of endogenous compounds to authentic standards [42], attaining a confidence level of 1 [81]. This class of hormones has been implicated in the improvement of human health conditions with the pharmacological activity of phytohormones occurring at femtomolar levels with observable variation in activity at increased levels in some instances [42,43,138]. However, endogenous phytohormones have not been explored in *E. neriifolia* or even the genus previously.

Latex is reported to contain terpenes, alkaloids, proteins, and sugars, which serve different roles in plant defense and physiology [145]. However, the concept of involvement of cytokinins in local and long-distance signaling with respect to its presence in xylem sap [136] makes the finding of CKs in LE intriguing. In the absence of literature for comparison, long-distance signaling of CKs in latex may be a less common phenomenon, as latex is a specialized, thick fluid exuded by the specific plant cells, laticifers, at the site of injury [137]. Although laticifers run throughout the plant system [137], it is possible that the cytokinins contribute to some long-distance signaling functions by the movement of latex within the plant. As indicated, latex is exuded when a plant is damaged [15] and offers protection from invading pathogens, insects, or herbivores to prevent further damage [26]. The specific role and significance of cytokinins in latex and their involvement in signaling is not well established, and the biological roles lack universal consensus [144]. The role of cytokinins in plants is more understood; therefore, release at the site of the wound could potentially contribute to local signaling events to promote cell division, tissue repair, and, thus, wound healing [40,45,174]. Although this could be a possible mechanism, more research would be required to fully understand their potential functions. With respect to the treatment of human disease states like skin infections for which latex is implicated, the thick substance may first act as a barrier, and cytokinins may offer similar protection in wound healing. The role in asthma does, however, require further elucidation.

Free base cytokinin iP and *t*Z types are the most active and important isoprenoid components in plants [40,136,175]. CK-NTs demonstrate a wide range of activity: anti-hypertensive, antiviral, antipsychotic, and anti-inflammatory [176]. Evidence indicates that iP-type cytokinins like iPR are the major forms of CKs in phloem sap, and *t*ZR is the major one found in xylem sap [136], which differs from the pattern seen in LE.

Given their limited distribution in nature but significant health applications of aromatic CKs [43], the presence of aromatic CKs in a species could signal major potential. Despite an incomplete understanding of the biogenesis of aromatic CKs and cellular targets in humans [177], they are important, as studies show improvement in health conditions with their application. The possibility, therefore, exists that these fractions play a role in the beneficial effects of the species. Kinetin, at a level of 42.39 pmol/g fwt (Table 4), was detected in the Traditional Extract and comprised the bulk of the aromatic cytokinins in the samples. While it was not reported to be directly involved in the treatment of asthma, kinetin has been studied for both antioxidant and anti-inflammatory properties [47,178,179], which could have positive effects on asthma resulting from the same triggers. Evidence suggests the activation of A2a-R receptors could cause increased intracellular cAMP levels without augmenting inositol triphosphate levels, unlike with activation of A1, A2B, and A3 receptors [180]. This leads to anti-inflammatory effects, bronchodilation, and tissue repair. Therefore, the inhibition of pro-inflammatory cytokines and agonistic effect on A2a-R receptors [47,177] could be potential mechanisms of action by which kinetin can exert its pharmacological effect in asthma.

Additionally, given the known effects of kinetin in DNA and tissue repair, receptor damage and epithelial tissue dysfunction [178,181], which occur in asthma, could be potentially improved by kinetin. To reiterate, the etiology of asthma is largely unknown, and genetic factors are implicated. Therefore, the possibility for action of kinetin at the genome level based on its involvement in genetic splicing for familial dysautonomia [182] could potentially be the mode of action. The action of kinetin in humans has gained much attention but has not been directed to respiratory disease; further studies are needed to establish a direct connection between kinetin and the treatment or management of asthma.

BAR, like kinetin, has demonstrated effects in oxidative stress [49] and was confirmed as one of only three cytokinin ribosides with cytotoxic properties [183]. The other two are iPR and KR. These contradict the study by Gonzalez and associates that indicates the positive effects of cZR [184]. Similarly, new information and research could determine the application to chronic respiratory issues.

GLUC and MeS cytokinin forms are mostly seen as inactive [40,45]. The greater number of storage O-glucosides (O-GLUCs) forms present in TE compared to LE and complete non-detection in LE was another remarkable occurrence. Given that hormones play a role in the regulation of plant growth [75] and latex forms part of the lactiferous system responsible mainly for plant defense [137], it is not surprising that CK storage forms are absent from latex samples. It appears, however, that the heating process allowed for more extraction of these metabolites. A noteworthy finding is that larger quantities of riboside-type O-GLUCs were exhibited for further elucidation. *t*Z types are the most abundantly occurring isoprenoids [43], and higher levels of trans-zeatin O-GLUCs were found in TE. The presence of riboside forms of zeatin O-GLUCs and the difference in tZOG and tZROG, for example, at almost twice the concentration of SE in TE, again points to potential extraction by heating.

In scanning for the presence of methylthiolated CKs, only one MeS form (i.e., MeSZR) was detected, with its presence found only in TE samples (Table 4). The expression of methylthiolated CK riboside fraction, MeSZR in TE alone in low amounts and consistent with the literature warrants more exploration as their origin, biological activity, and functions are poorly understood [45].

Detection of acidic phytohormones was noticeable in SE from *Euphorbia neriifolia.* GA_7_ was detected in LE. Although IAA and JA derivatives were identified as putative compounds in heated samples in the global screening, they were not further investigated due to the preference for confidence level 1 for the hormones and the unavailability of the standards. This provided a rationale, however, for potentially altering the methodology for future detection. Interestingly, the appearance of GA in latex could be an indication of its use in asthma, as it is reported to act by inducing the anti-inflammatory A20 protein in lung epithelia tissues [37].

## 5. Conclusions

This study was undertaken to comparatively evaluate the phytoconstituents of traditional heated leaf extract, latex and simple extracts of *E. neriifolia* and their potential roles with respect to use of the species in asthma therapy. The simple extract was used as a control for comparison as it is thought to have less therapeutic impact. The presence of phytochemicals and phytohormones was investigated using UPLC MS/MS untargeted and targeted metabolomic approaches to determine and develop a chemical profile. A greater number and more upregulated features were detected in the traditional extracts, indicating the availability of possibly increased metabolites from which potential therapeutic benefit could be derived, and biologically active metabolites for treating inflammatory diseases were identified. Identification of characteristic compounds such as quercetin, myricetin, and kaempferol indicate sweet aloe constituents are consistent with *E. neriifolia* species reported globally, which would imply alternative applications of sweet aloe beyond the few current traditional uses for asthma, rashes, cough, and colds ailments in Guyana. Although the method was optimized for cytokinin extraction, with optimized parameters in positive ionization mode, future studies can examine other metabolites and phytohormone classes in negative ionization mode.

Heat applications could cause increased extraction of phenolics and other metabolites [185]. This supports incorporating Indigenous methods into scientific evaluations of natural products to accurately identify components in traditional therapies [186]. Integrating traditional knowledge with Western methodologies could yield significant benefits in healthcare as extract types exhibited considerable differences and variations in metabolite profiles. The presence of phytohormones of the aromatic cytokinin type in the traditional remedy presents a stimulus for further testing of the sample types. This finding was interesting as aromatics like kinetin are found to have limited occurrence in nature, as indicated previously. Generally, pharmacological activities of natural products may be derived from the collective contributions of phytoconstituents from different categories. Therapeutic benefits for asthma could potentially be a function of both phytochemicals and phytohormones but further research is required to link phytohormones such as kinetin with asthma roles.

There is considerable scope for future work on *E. neriifolia*. Overall, *E. neriifolia* contains thousands of features with potential therapeutic activity in each extract type, with most features observed in the traditional extract. It is possible that reported benefits in asthma are not exclusively the result of a single metabolite or only secondary metabolites but may also be attributed to the effects of phytohormones or synergy between groups. Further research on the species could facilitate the broader application of an alternative remedy for chronic respiratory and other disorders.

## Figures and Tables

**Table 1:** *Euphorbia* sp. sample types used in this study and their traditional uses.

Sample Type	Definitions	Source	Uses in Guyana	Uses in Other Countries	Source
Simple Leaf Extract (SE)	Content extracted from leaf tissue at normal temperature	Leaf	No known use	Earaches, wounds, CNS depressant, aphrodisiac, adjunct in COVID-19 treatment	[15,69]
Traditional Extract (TE)	Clear, light yellowish liquid obtained from squeezing heated leaves in a process that mimics the traditional mode of preparation of the remedy	Leaf	Wheezing, asthma, coughs, colds	Earache, epilepsy	[15,17,70]
Latex Extract (LE)	Milky white exudate from damaged plant parts, broken leaves, and stem	Latex	Rashes	Asthma, bronchitis, syphilis, leprosy, gonorrhea, rashes, tumors, healing cracks on the soles of feet	[15,19,70]

**Table 4:** Phytohormone concentrations in three sample types extracts of *E. neriifolia*. These were reported as the mean ± the standard error [pmol/g fwt].

Phytohormone Class	Compound	Simple Extract (SE)	Traditional Extract (TE)	Latex Extract (LE)
**Cytokinin**				
Aromatics	BA	-	-	-
	BAR	-	0.29 ± 0.11	-
	KIN	-	42.39 ± 18.95	-
Freebases	iP	2.32 ± 0.12	1.16 ± 0.1	2.46 ± 0.13
	*t*Z	0.7 ± 0.32	1.5 ± 0.38	-
	cZ	-	0.64 ± 0.09	-
	DZ	-	-	-
Ribosides	iPR	16.7 ± 1.95	10.5 ± 3.08	2.65 ± 0.48
	*t*ZR	1.85 ± 0.35	1.25 ± 0.39	0.17 ± 0.05
	cZR	1.24 ± 0.19	1.35 ± 0.54	4.13 ± 0.48
	DZR	14.23 ± 2.97	0.41 ± 0.19	10.84 ± 1.16
Nucleotides	iPNT	261.62 ± 52.27	19.2 ± 0.98	-
	*t*ZNT	10.2 ± 3.39	4.44 ± 0.92	0.25 ± 0.08
	cZNT	8 ± 2.97	45.13 ± 1.57	1.92 ± 0.44
	DZNT	-	0.08 ± 0.02	-
Glucosides	iP9G	-	0.14 ± 0.04	-
	*t*Z9G	-	0.38 ± 0.21	-
	*t*ZOG	8.2 ± 2.76	18.62 ± 3.1	-
	cZOG	-	2.78 ± 0.55	-
	DZOG	-	0.39 ± 0.07	-
	*t*ZROG	22.29 ± 14.37	41.9 ± 16.37	-
	cZROG	17.42 ± 4.16	28.9 ± 10.12	-
	DZROG	3.66 ± 1.59	10.65 ± 3.81	-
Methylthiols	MeSZ	-	-	-
	MeSZR	-	0.86 ± 0.34	-
**Acidic Hormones**	ABA	61.74 ± 11.37	-	-
	GA_7_	-	-	0.53 ± 0.09
	IAA	381.11 ± 88.38	-	-
	JA	220.51 ± 35.71	-	-
**TOTAL**	**pmol/g fwt**	**1031.79**	**232.96**	**22.95**

## Data Availability

All relevant data generated or analyzed during this study are included in this published article and Supplementary Materials. Other raw data are available upon request.

## Supplementary Materials

The following supporting information can be downloaded at https://www.mdpi.com/article/10.3390/metabo15030177/s1, Figure S1. Violin plots of therapeutic metabolites showing peak areas according to sample type (*n* = 5). The star symbol (*) signifies statistical difference between the referenced control (i.e., unheated sample) and the treatment investigated (i.e., heated or latex samples). The term “ns” means not significant. Figure S2. Fold change (FC) analysis of therapeutic metabolites detected in this study. Fold change was calculated using quantified peak areas with the treatment/control (*n* = 5). Treatment/control corresponds to [Heated]/[Unheated] or [Latex]/[Unheated] comparisons of average intensities of each metabolite. Compounds were considered upregulated in a given treatment (i.e., treatment/control) when there is a log_2_FC > 1, or downregulated with a log_2_FC < −1. Compounds that were not detected were not shown in the table. Figure S3. Bar plots of quantified phytohormones levels according to sample type (*n* = 5). The star symbol (*) signifies statistical difference between the referenced control (i.e., unheated samples) and the treatment investigated (i.e., heated or latex samples). Asterisks * for *p*-value < 0.05, ** for *p*-values < 0.01, **** for *p*-values < 0.001. The term “ns” means not significant. Bar plots were generated using Metabolite Autoplotter. Figure S4. PCA biplot (a) Variable importance of projection (VIP) (b) and dendogram (c) comparing phytohormone data in simple, traditional heated, and latex extracts. Phytohormone data with 50% of values missing (i.e., not detected) were eliminated from the dataset via MetaboAnalyst 6.0. Figure S5. Correlation analyses of (a) phytohormones with sample types and (b) phytohormones with each other. Phytohormone data with 50% of values missing (i.e., not detected) were eliminated from the dataset via MetaboAnalyst 6.0. Figure S6. Self-organizing maps (SOM) of (a) phytohormones present in every sample type with respect to endogenous levels and (b) therapeutic metabolites belonging to every sample type with respect to intensities. LivingForSOM (v. 1.4.4) was used to generate the SOMs. Phytohormones or therapeutic metabolites with missing values were automatically removed using the software. Figure S7. Overview of the molecular network of *E. neriifolia* via the FBMN work module in GNPS. Data was visualized using Cytoscape 3.10.3. Table S1. Endogenous and isotope-labeled phytohormones were scanned for using the Q Exactive Orbitrap mass spectrometer and their classifications, abbreviations, and their associated deuterated internal standard via isotopic dilution technique. Table S2: Results from Pathway Analysis. The table below shows the detailed results from the pathway analysis. Since many pathways are tested at the same time, the statistical *p* values from enrichment analysis are further adjusted for multiple testings. In particular, the Total is the total number of compounds in the pathway; the Hits is the actually matched number from the user-uploaded data; the Raw *p* is the original *p* value calculated from the enrichment analysis; the Holm *p* is the *p*-value adjusted by the Holm–Bonferroni method; the FDR *p* is the *p*-value adjusted using False Discovery Rate; the Impact is the pathway impact value calculated from pathway topology analysis. Table S3. Therapeutic compounds at level 3 annotation level via querying MetaboQuest and CEU Mediator 3.0. Table S4. Level 3 metabolite annotations as generated by SIRIUS 5.6.3 for sample-specific metabolites. A zodiac score (over 95%) and a similarity score (over 75%) were chosen as cutoffs for this table. Table S5. Level 3 metabolite annotations as generated by SIRIUS 5.6.3 for other metabolites. Table S6. Level 2 metabolite annotations as generated by MS-DIAL 5.1 using various downloadable public databases in positive ionization mode. Table S7. Level 2 annotation of selected compounds derived from feature-based molecular networking via GNPS with fragmentation data grouped according to natural product classification. Other annotated metabolites not listed in this table can be viewed at: https://gnps.ucsd.edu/ProteoSAFe/status.jsp?task=685a12e4b3674ae0baebafcd1fd3f3de; accessed on 24 February 2025. Table S8. Fold change (FC) analysis of therapeutic metabolites detected in this study. Fold change was calculated using quantified peak areas with the treatment/control (n = 5). Treatment/control corresponds to [Heated]/[Unheated] or [Latex]/[Unheated] comparisons of average intensities of each metabolite. Compounds were considered upregulated in each treatment (i.e., treatment/control) when there is a log_2_FC > 1, or downregulated with a log_2_FC < −1. Compounds that were not detected were not shown in the table. Table S9. Other semi-targeted metabolites via the KNapSAcK database specific to the Euphorbia species. A custom database made from queried metabolites in KNapSAcK was built to screen the XCMS outline output for putative metabolites within a 5-ppm error margin using HormonomicsDB. This annotation would be at level 3. Isomeric compounds are separated by “//”. It should be noted that some compounds may have more than 3 isomers that may not be listed on the table for brevity. Peak quality for these queried compounds was not checked. It should be noted that, to date, compounds specific to Euphorbia species in KNapSAcK or in literature may not be present in other databases. Table S10. Other semi-targeted metabolites via the KNapSAcK database specific to the Euphorbia species at annotation level 3 depicting up- or downregulation of heated/traditional and latex extracts with respect to unheated/simple extracts. Compounds were considered upregulated in each treatment (i.e., treatment/control) when there is a log_2_FC > 1 or downregulated with a log_2_FC < −1. Table S11. Level 3 annotation of putative metabolites via screening the top 15 masses of the VIP of heated (TE) vs. unheated (SE) and latex (LE) vs. unheated (SE) comparisons. CEU Mass Mediator (CMM 3.0) was used to query masses in positive ionization mode. Metabolites that were detected at level 2 confirmation in metabolite annotations via GNPS and MS-DIAL are indicated in the table. Table S12. Level 3 annotation using MS2Compound showing candidates that are ranked 1. Annotation was conducted via the mzMine workflow. Additional Supplementary Information can be seen at: https://zenodo.org/records/14876895; accessed on 24 February 2025 or https://doi.org/10.5281/zenodo.14876894.
