# Supplementary material for: Untargeted Metabolomics and Targeted Phytohormone Profiling of Sweet Aloes (Euphorbia neriifolia) from Guyana: An Assessment of Asthma Therapy Potential in Leaf Extracts and Latex
Source: Metabolites. 2025 Mar 5;15(3):177. doi: 10.3390/metabo15030177 (PMC11943701; doi:10.3390/metabo15030177)
Supplement: Supplementary file 1 [file metabolites-15-00177-s001.zip › metabolites-3441661-supplementary.pdf]

Supplemental Information

Untargeted Metabolomics and Targeted Phytohormone Profiling of Sweet Aloes (*Euphorbia neriifolia*) from Guyana: An Assessment of Asthma Therapy Potential in Leaf Extracts and Latex

Malaika Persaud [1], Ainsely Lewis [2,3,*], Anna Kisiala [2], Ewart A. Smith [4], Zeynab Azimychetabi [4], Tamanna Sultana [5], Suresh S. Narine [6,7] and R. J. Neil Emery [2]

[1] Sustainability Studies Graduate Program, Faculty of Arts and Science, Trent University, Peterborough, ON K9J 0G2, Canada
[2] Department of Biology, Trent University, Peterborough, ON K9J 0G2, Canada
[3] Department of Biology, University of Toronto Mississauga, Mississauga, ON L5L 1C6, Canada
[4] Environmental and Life Sciences Graduate Program, Trent University, Peterborough, ON K9J 0G2, Canada
[5] Department of Chemistry, Trent University, Peterborough, ON K9J 0G2, Canada
[6] Trent Centre for Biomaterials Research, Trent University, Peterborough, ON K9J 0G2, Canada
[7] Departments of Physics & Astronomy and Chemistry, Trent University, Peterborough, ON K9J 0G2, Canada
* Correspondence: ainselylewis@alumni.trentu.ca or ainsely.lewis@utoronto.ca

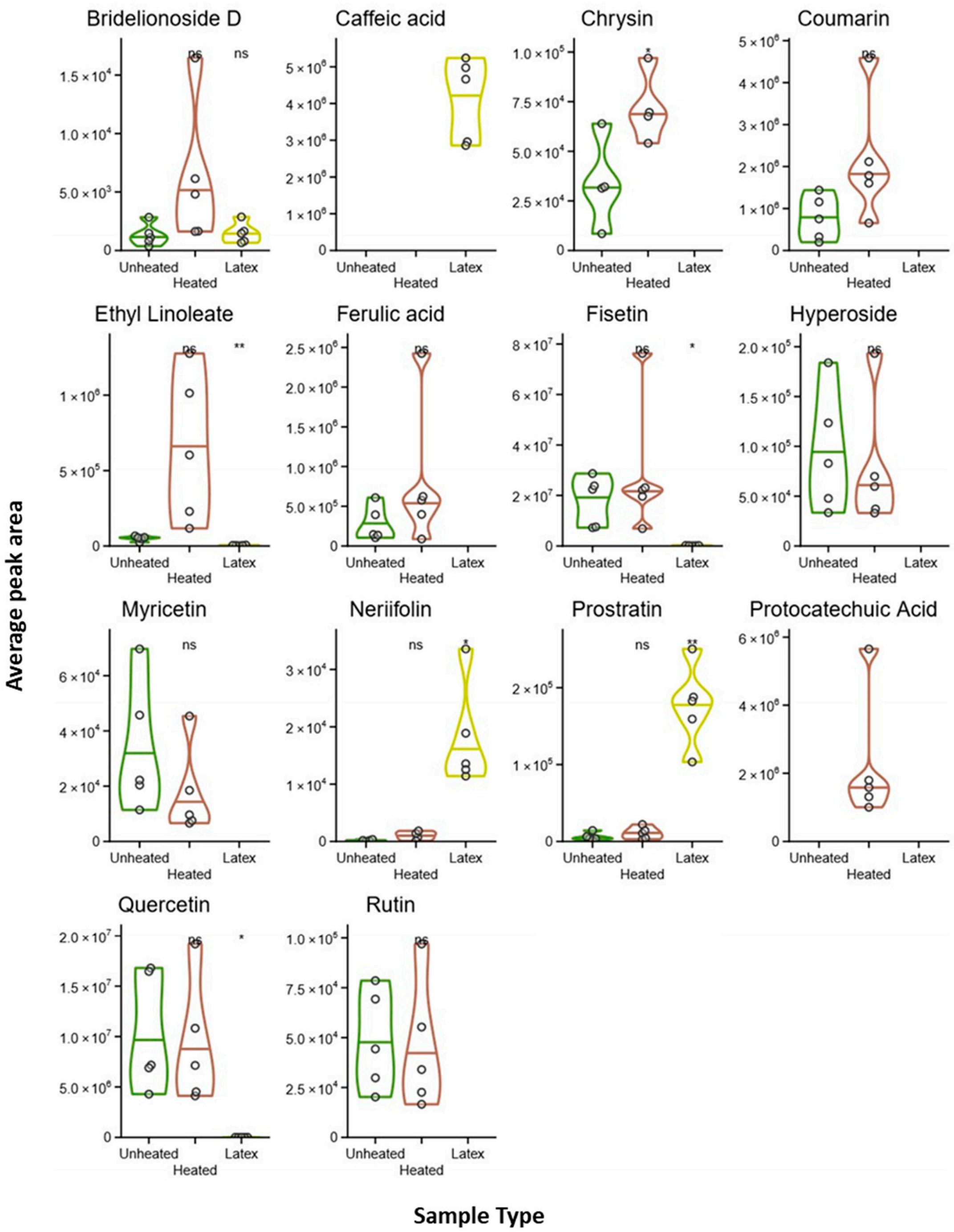

Figure S1. Violin plots of therapeutic metabolites showing peak areas according to sample type (n=5). The star symbol (*) signifies statistical difference between the referenced control (i.e., Unheated sample) and the treatment investigated (i.e., Heated or Latex samples). The term "ns" means not significant.

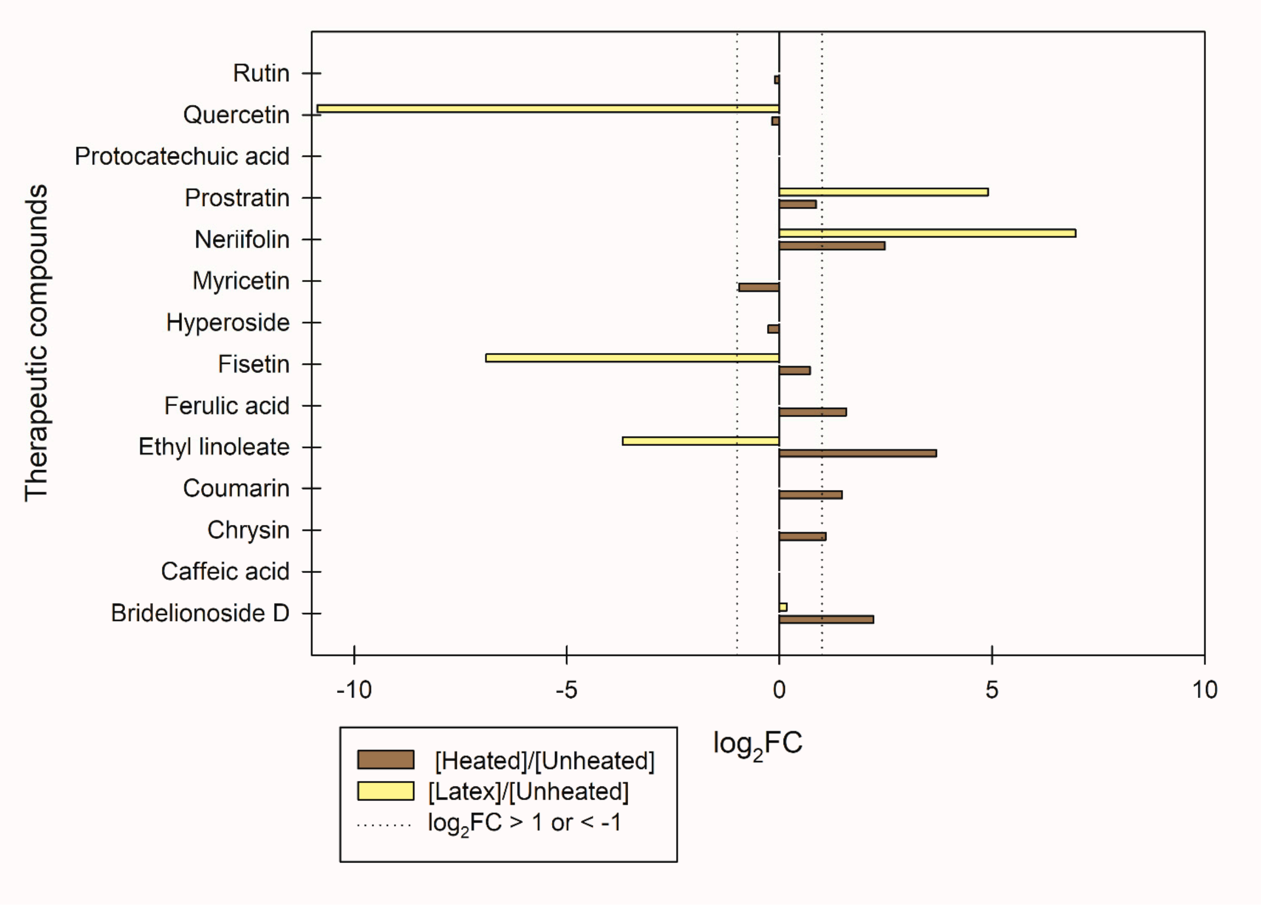

Figure S2. Fold change (FC) analysis of therapeutic metabolites detected in this study. Fold change was calculated using quantified peak areas with the treatment/control (n=5). Treatment/control corresponds to [Heated]/[Unheated] or [Latex]/[Unheated] comparisons of average intensities of each metabolite. Compounds were considered upregulated in a given treatment (i.e., treatment/control) when there is a $\log_2 FC > 1$, or downregulated with a $\log_2 FC < -1$. Compounds that were not detected were not shown in the table.

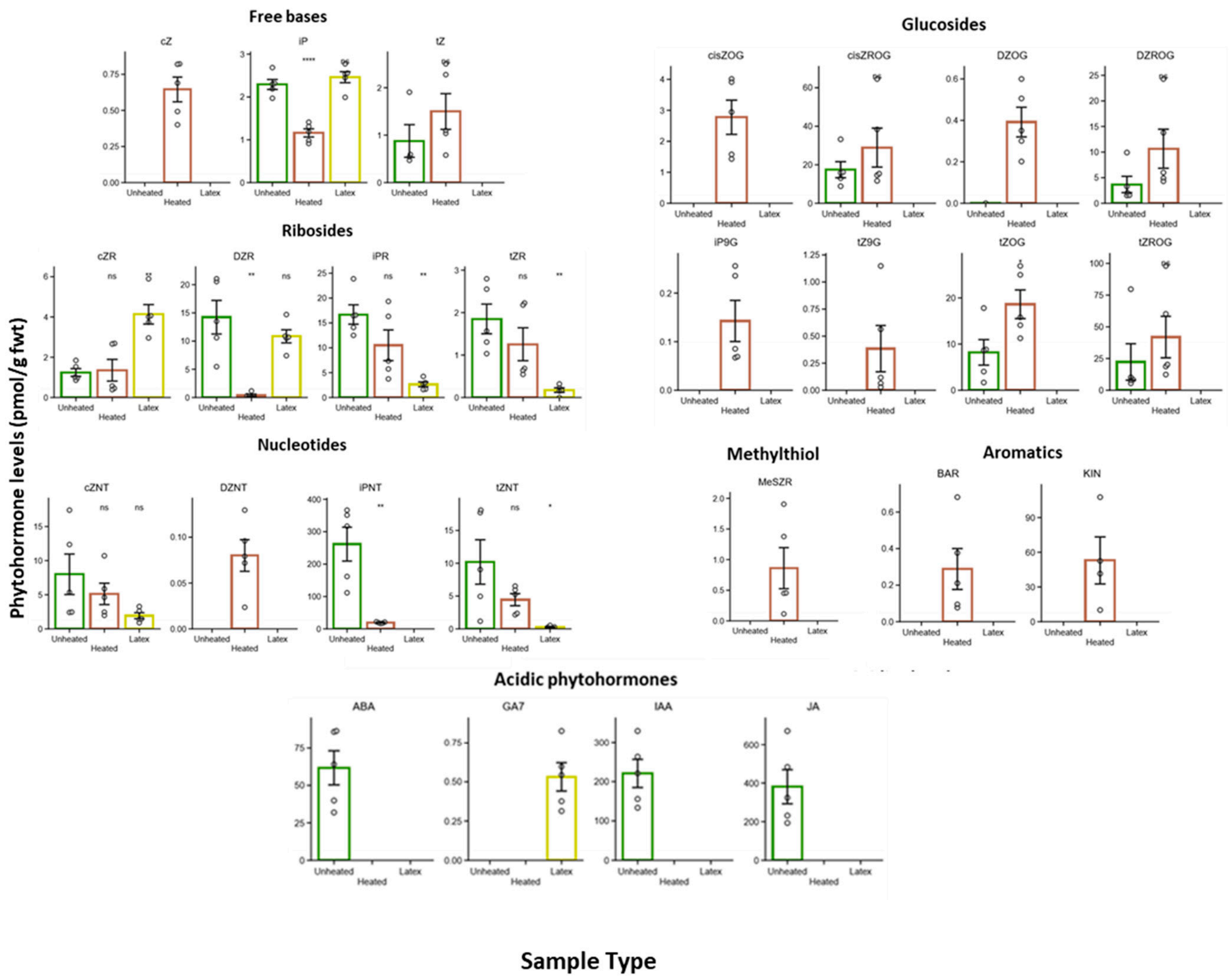

Figure S3. Bar plots of quantified phytohormones levels according to sample type (n=5). The star symbol (*) signifies statistical difference between the referenced control (i.e., Unheated samples) and the treatment investigated (i.e., Heated or Latex samples). Asterisks * for p-value < 0.05, ** for p-values < 0.01, **** for p-values < 0.001. The term "ns" means not significant. Bar plots were generated using Metabolite Autoplotter.

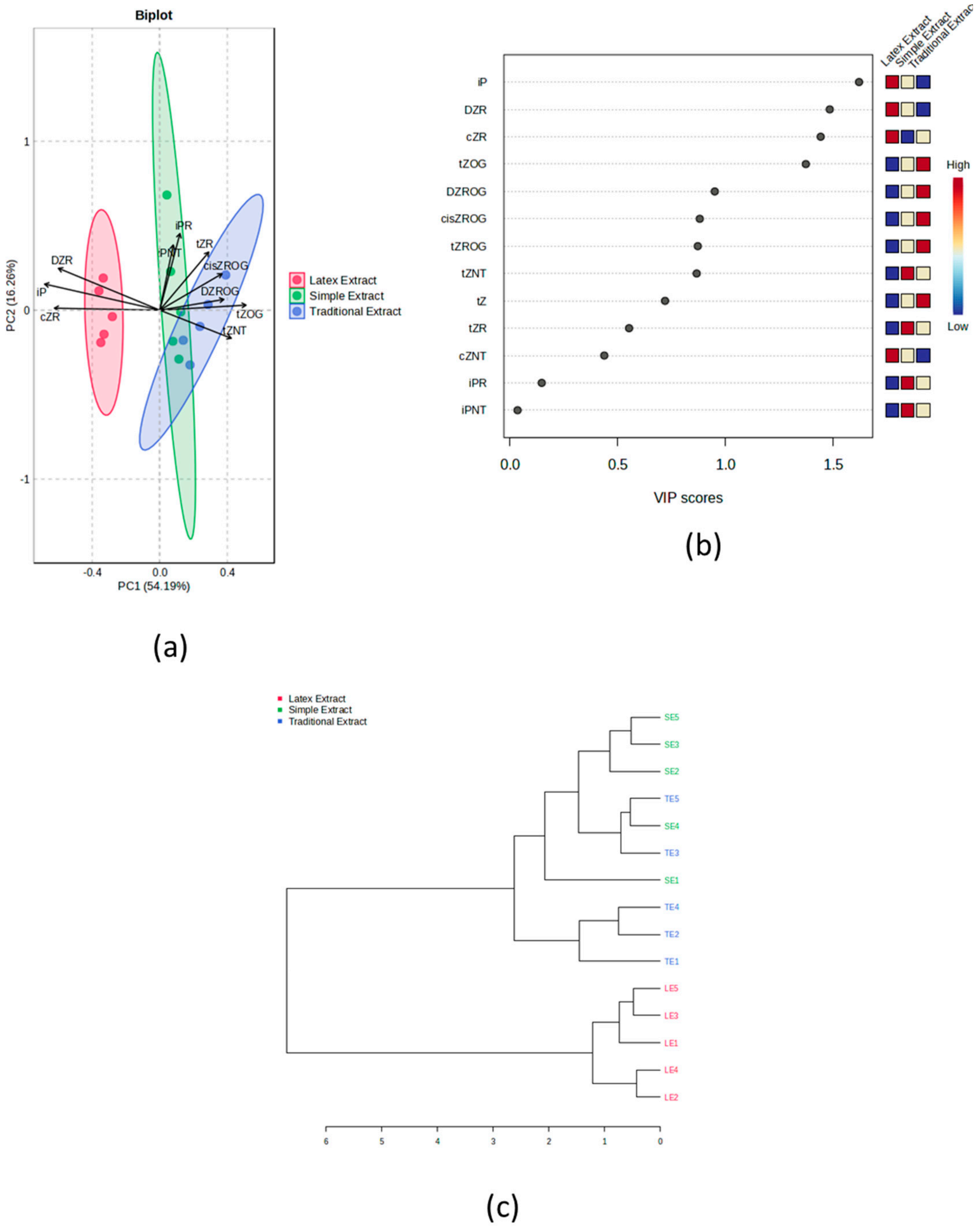

Figure S4. PCA biplot (a) Variable importance of projection (VIP) (b) and dendrogram (c) comparing phytohormone data in simple, traditional heated and latex extracts. Phytohormone data with 50% of values missing (i.e., not detected) were eliminated from the dataset via MetaboAnalyst 6.0.

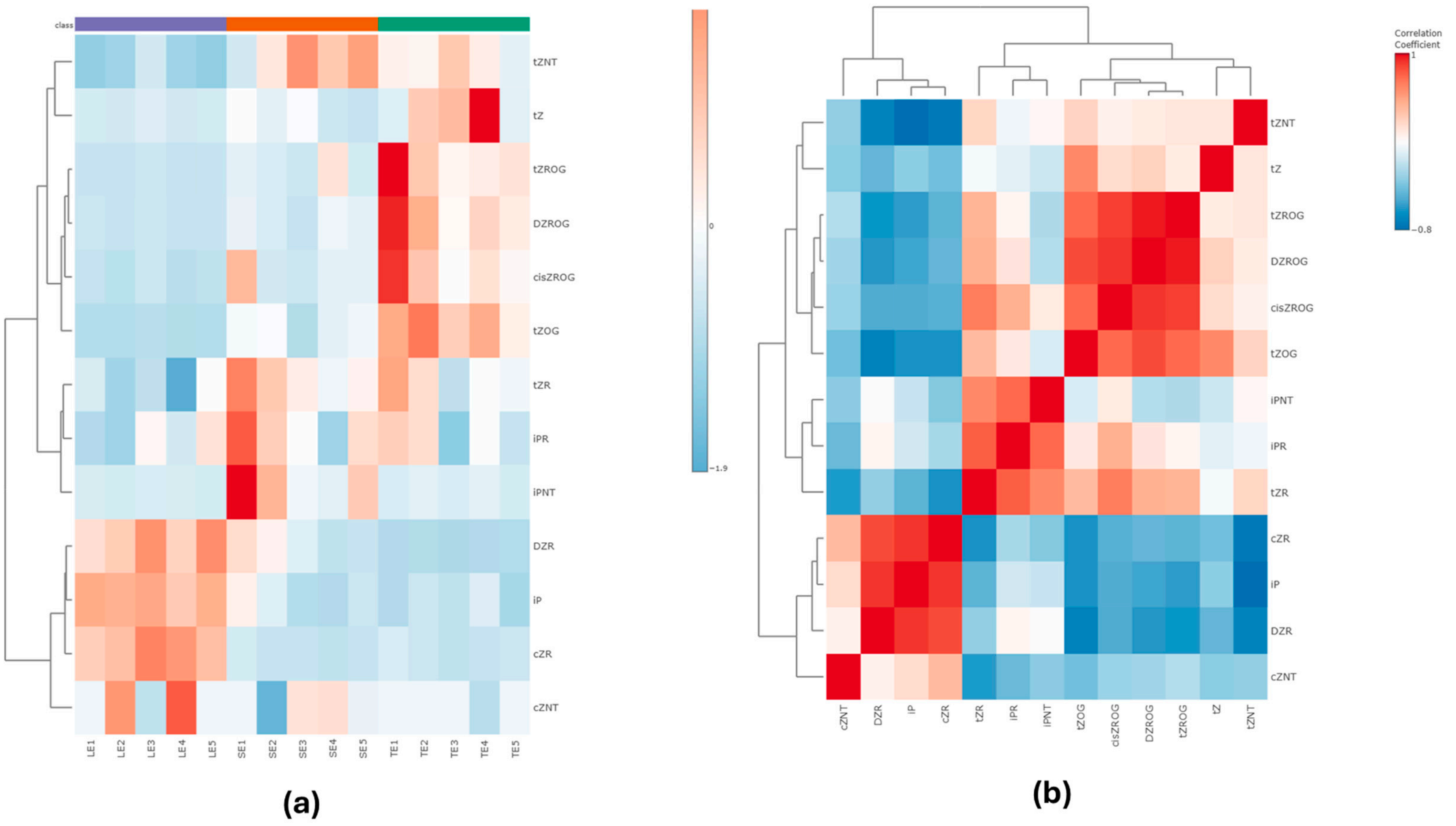

Figure S5. Correlation analyses of (a) phytohormones with sample types and (b) phytohormones with each other. Phytohormone data with 50% of values missing (i.e., not detected) were eliminated from the dataset via MetaboAnalyst 6.0.

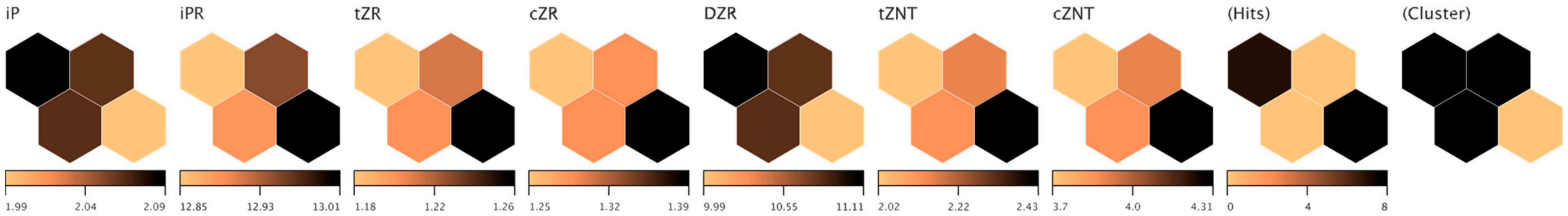

(a)

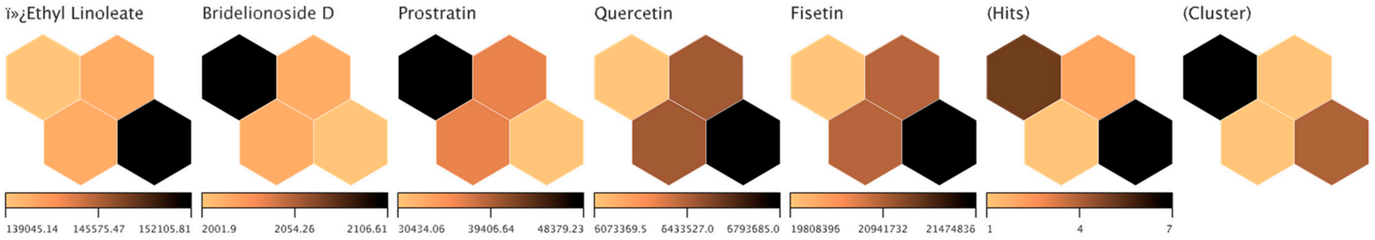

(b)

Figure S6. Self-organizing maps (SOM) of (a) phytohormones present in every sample type with respect to endogenous levels and (b) therapeutic metabolites belonging to every sample type with respect to intensities. LivingForSOM (v. 1.4.4) was used to generate the SOMs. Phytohormones or therapeutic metabolites with missing values were automatically removed using the software.

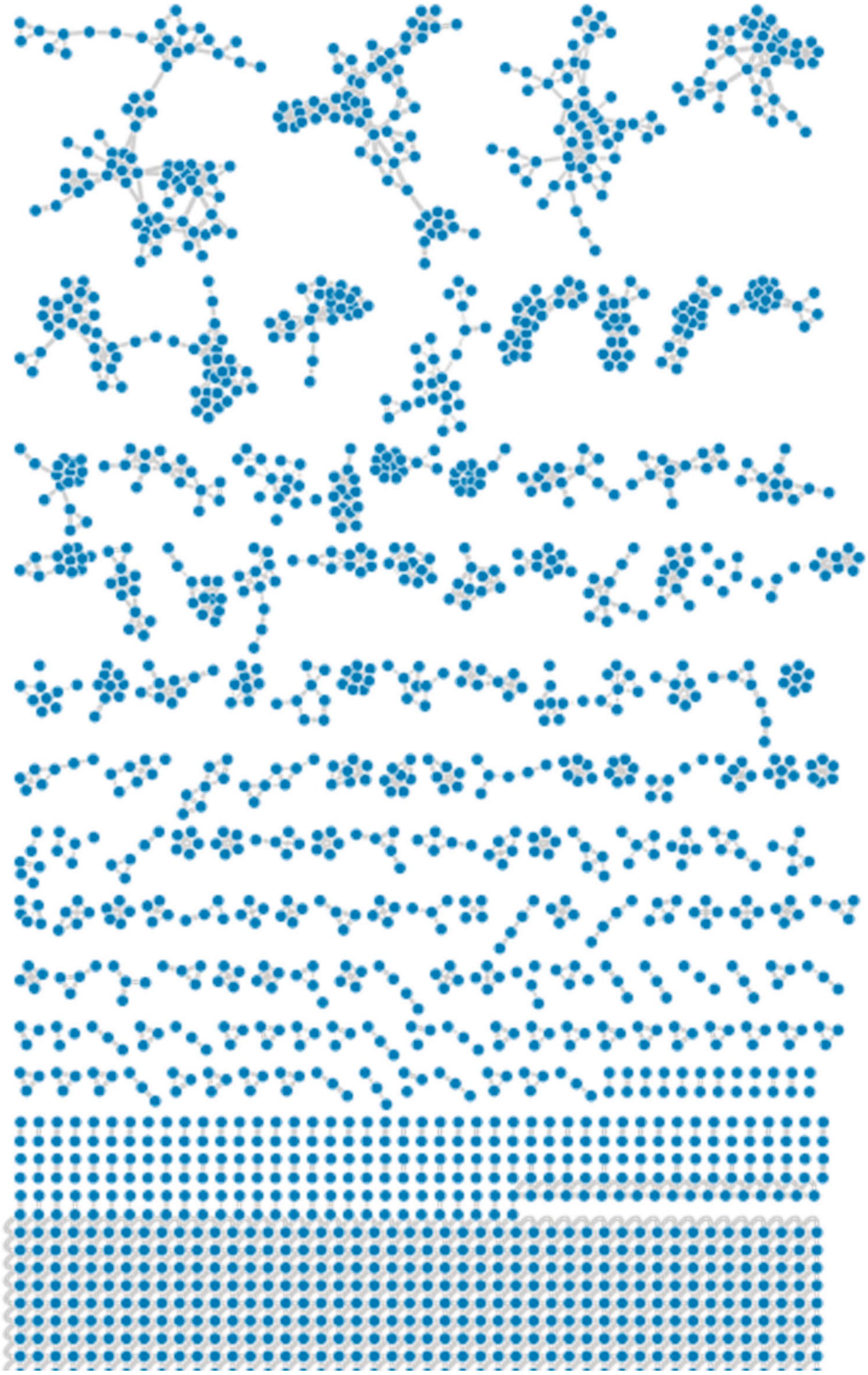

Figure S7. Overview of the molecular network of *E. neriifolia* via the FBMN work module in GNPS. Data was visualized using Cytoscape 3.10.3.

Table S1. Endogenous and isotope labeled phytohormones scanned for using the Q Exactive Orbitrap mass spectrometer and their classifications, abbreviations, and their associated deuterated internal standard via isotopic dilution technique.

Endogenous CK phytohormones	Abbreviation	^{2}H-labelled Internal Standards
**Free bases (FB)**		
N^6-isopentenyladenine	iP	$^2H_6$iP
trans-zeatin	*t*Z	$^2H_3$DZ
cis-zeatin	cZ	$^2H_3$DZ
Dihydrozeatin	DZ	$^2H_3$DZ
**Ribosides (RB)**		
N^6-isopentyadenosine	iPR	$^2H_6$[9R]iP
trans-zeatin riboside	*t*ZR	$^2H_5$[9R]tZ
cis-zeatin riboside	cZR	$^2H_5$[9R]tZ
Dihydrozeatin riboside	DZR	$^2H_3$[9R]DZ
**Nucleotides (NT)**		
N^6-isopentyadenosine-5'-monophosphate	iPRP	$^2H_6$[9RMP]iP
trans-zeatin riboside-5'-monophosphate	*t*ZRP	$^2H_6$[9RMP]DZ
cis-zeatin riboside -5'-monophosphate	cZRP	$^2H_6$[9RMP]DZ
Dihydrozeatin riboside-5'-monophosphate	DZRP	$^2H_6$[9RMP]DZ
**Glucosides (GLUC)**		
isopentenyladenine-7-glucoside	iP7G	$^2H_6$iP
isopentenyladenine-9-glucoside	iP9G	$^2H_6$iP
trans-zeatin-O-glucoside	*t*ZOG	$^2H_5$tZOG
cis-zeatin-O-glucoside	cZOG	$^2H_5$tZOG
Dihydrozeatin-O-glucoside	DZOG	$^2H_7$DZOG
trans-zeatin-O-glucoside riboside	*t*ZROG	$^2H_5$tZROG
cis-zeatin-O-glucoside riboside	cZROG	$^2H_5$tZROG
Dihydrozeatin-O-glucoside riboside	DZROG	$^2H_7$DZROG
trans-zeatin-9-glucoside	*t*Z9G	$^2H_5$tZ9G
cis-zeatin-9-glucoside	cZ9G	$^2H_5$tZ9G
Dihydrozeatin-9-glucoside	DZ9G	$^2H_3$DZ9G
**Methylthiols (2MeS)**		
2-Methylthio- N^6-isopentayladenine	2MeSiP	$^2H_6$2MeSiP
2-Methylthio-N^6-isopentenyladenosine	2MeSiPR	$^2H_6$2MeSiPR
2-Methylthio-zeatin	2MeSZ	$^2H_5$2MeStZ
2-Methylthio-zeatin riboside	2MeSZR	$^2H_5$2MeStZR
**Aromatic CKs**		
Kinetin	KIN	$^2H_7$BA
N^6-benzyladenine	BA	$^2H_7$BA
N^6-benzyladenosine	BAR	$^2H_7$[9R]BA
**Acidic Phytohormones**		
Abscisic acid	ABA	[$^2H_4$] ABA
Indole-3-Acetic Acid	IAA	[$^2H_5$] IAA
Jasmonic acid	JA	[$^2H_6$] JA (not added)
Salicylic acid	SA	[$^2H_4$]SA
Gibberellin 1	GA_1	[$^2H_4$]GA_1
Gibberellin 4	GA_4	[$^2H_2$]GA_4
Gibberellin 7	GA_7	[$^2H_2$]GA_7
Gibberellin 9	GA_9	[$^2H_2$]GA_9
Gibberellin 20	GA_{20}	[$^2H_2$]GA_{20}

Table S2: Results from Pathway Analysis. The table below shows the detailed results from the pathway analysis. Since many pathways are tested at the same time, the statistical p values from enrichment analysis are further adjusted for multiple testings. In particular, the Total is the total number of compounds in the pathway; the Hits is the actually matched number from the user uploaded data; the Raw p is the original p value calculated from the enrichment analysis; the Holm p is the p value adjusted by Holm-Bonferroni method; the FDR p is the p value adjusted using False Discovery Rate; the Impact is the pathway impact value calculated from pathway topology analysis.

	**Total**	**Expected**	**Hits**	**Raw p**	**-log10(p)**	**Holm adjust**	**FDR**	**Impact**
Phenylpropanoid biosynthesis	46	6.76	25	1.7543e-10	9.7559	1.6841e-08	1.6841e-08	0.64
Diterpenoid biosynthesis	28	4.12	18	1.8167e-09	8.7407	1.7259e-07	7.3816e-08	0.73
Flavonoid biosynthesis	47	6.91	24	2.3068e-09	8.637	2.1684e-07	7.3816e-08	0.50
Tyrosine metabolism	16	2.35	12	8.185e-08	7.087	7.612e-06	1.9644e-06	0.76
Flavone and flavonol biosynthesis	10	1.47	8	6.6999e-06	5.1739	0.000616	0.000129	0.85
Isoquinoline alkaloid biosynthesis	6	0.88	6	9.5094e-06	5.0218	0.000865	0.000152	1.00
Monoterpenoid biosynthesis	9	1.32	7	3.7622e-05	4.4246	0.003386	0.000516	0.38
Phenylalanine metabolism	11	1.62	6	0.0022866	2.6408	0.20351	0.025277	0.94
Phenylalanine, tyrosine and tryptophan biosynthesis	22	3.23	9	0.0023697	2.6253	0.20853	0.025277	0.25
alpha-Linolenic acid metabolism	28	4.12	10	0.0044364	2.353	0.38597	0.042589	0.38
Linoleic acid metabolism	4	0.59	3	0.011189	1.9512	0.96224	0.097648	1.00
Cyanoamino acid metabolism	29	4.26	9	0.018707	1.728	1	0.13435	0.27
Stilbenoid, diarylheptanoid and gingerol biosynthesis	8	1.18	4	0.019593	1.7079	1	0.13435	0.53
One carbon pool by folate	8	1.18	4	0.019593	1.7079	1	0.13435	0.76
Biosynthesis of unsaturated fatty acids	22	3.23	7	0.032303	1.4908	1	0.19382	0.00
Valine, leucine and isoleucine biosynthesis	22	3.23	7	0.032303	1.4908	1	0.19382	0.14
Betalain biosynthesis	3	0.44	2	0.058294	1.2344	1	0.32919	1.00
Sphingolipid metabolism	17	2.50	5	0.090952	1.0412	1	0.48508	0.44
Cutin, suberine and wax biosynthesis	18	2.65	5	0.11154	0.95258	1	0.56355	0.44
Porphyrin and chlorophyll metabolism	48	7.06	10	0.1551	0.80938	1	0.69396	0.31
Citrate cycle (TCA cycle)	20	2.94	5	0.15864	0.7996	1	0.69396	0.22
Limonene and pinene degradation	5	0.74	2	0.15903	0.79851	1	0.69396	0.00

Riboflavin metabolism	11	1.62	3	0.2116	0.67449	1	0.86511	0.34
Biosynthesis of secondary metabolites - other antibiotics	6	0.88	2	0.21628	0.66499	1	0.86511	0.00
Arachidonic acid metabolism	12	1.76	3	0.25346	0.59609	1	0.97329	0.00
Nicotinate and nicotinamide metabolism	13	1.91	3	0.29636	0.52819	1	1	0.02
Ubiquinone and other terpenoid-quinone biosynthesis	38	5.59	7	0.32049	0.49418	1	1	0.21
Tropane, piperidine and pyridine alkaloid biosynthesis	8	1.18	2	0.33334	0.47712	1	1	0.00
Aminoacyl-tRNA biosynthesis	46	6.76	8	0.36163	0.44173	1	1	0.17
Zeatin biosynthesis	21	3.09	4	0.37329	0.42795	1	1	0.15
Thiamine metabolism	22	3.23	4	0.40905	0.38822	1	1	0.12
Alanine, aspartate and glutamate metabolism	22	3.23	4	0.40905	0.38822	1	1	0.20
Pyruvate metabolism	22	3.23	4	0.40905	0.38822	1	1	0.22
Indole alkaloid biosynthesis	4	0.59	1	0.47095	0.32702	1	1	0.00
Anthocyanin biosynthesis	11	1.62	2	0.49689	0.30374	1	1	0.33
Arginine biosynthesis	18	2.65	3	0.50663	0.29531	1	1	0.00
Biosynthesis of secondary metabolites - unclassified	5	0.74	1	0.54893	0.26048	1	1	1.00
Folate biosynthesis	27	3.97	4	0.57733	0.23858	1	1	0.14
C5-Branched dibasic acid metabolism	6	0.88	1	0.61546	0.2108	1	1	0.50
Carbon fixation in photosynthetic organisms	21	3.09	3	0.61646	0.2101	1	1	0.11
Glucosinolate biosynthesis	65	9.56	9	0.63437	0.19766	1	1	0.03
Glyoxylate and dicarboxylate metabolism	29	4.26	4	0.63668	0.19608	1	1	0.14
Histidine metabolism	15	2.21	2	0.67124	0.17312	1	1	0.08
Phosphonate and phosphinate metabolism	7	1.03	1	0.67222	0.17249	1	1	0.00
Monobactam biosynthesis	8	1.18	1	0.72063	0.14229	1	1	0.00
Glycine, serine and threonine metabolism	33	4.85	4	0.73826	0.13179	1	1	0.03
Lysine biosynthesis	9	1.32	1	0.76192	0.11809	1	1	0.00
Lysine degradation	18	2.65	2	0.76733	0.11502	1	1	0.00
beta-Alanine metabolism	18	2.65	2	0.76733	0.11502	1	1	0.00
Biotin metabolism	18	2.65	2	0.76733	0.11502	1	1	0.17
Carotenoid biosynthesis	43	6.32	5	0.78149	0.10708	1	1	0.01
Pentose phosphate pathway	19	2.79	2	0.79348	0.10047	1	1	0.12
Tryptophan metabolism	28	4.12	3	0.8039	0.094796	1	1	0.12

Vitamin B6 metabolism	11	1.62	1	0.82716	0.082412	1	1	0.21
Terpenoid backbone biosynthesis	30	4.41	3	0.84086	0.075277	1	1	0.07
Pantothenate and CoA biosynthesis	23	3.38	2	0.87387	0.058553	1	1	0.27
Sesquiterpenoid and triterpenoid biosynthesis	24	3.53	2	0.88889	0.051151	1	1	0.20
Glutathione metabolism	26	3.82	2	0.9141	0.039007	1	1	0.01
Cysteine and methionine metabolism	46	6.76	4	0.925	0.033859	1	1	0.05
Valine, leucine and isoleucine degradation	37	5.44	3	0.92684	0.032997	1	1	0.01
Butanoate metabolism	17	2.50	1	0.93405	0.02963	1	1	0.00
Ascorbate and aldarate metabolism	18	2.65	1	0.94386	0.025094	1	1	0.00
Arginine and proline metabolism	34	5.00	2	0.97052	0.012998	1	1	0.04
Starch and sucrose metabolism	22	3.23	1	0.97055	0.012983	1	1	0.00
Fatty acid elongation	23	3.38	1	0.97494	0.011021	1	1	0.00
Fatty acid biosynthesis	56	8.23	4	0.9751	0.010953	1	1	0.01
Glycolysis / Gluconeogenesis	26	3.82	1	0.98458	0.0067488	1	1	0.11
Fatty acid degradation	37	5.44	1	0.99742	0.0011201	1	1	0.00
Pyrimidine metabolism	38	5.59	1	0.99781	0.00095103	1	1	0.03
Steroid biosynthesis	45	6.62	1	0.99931	0.0003017	1	1	0.03
Amino sugar and nucleotide sugar metabolism	50	7.35	1	0.9997	0.00013242	1	1	0.00
Purine metabolism	63	9.26	1	0.99996	1.5344e-05	1	1	0.00

Table S3. Therapeutic compounds at level 3 annotation level via querying MetaboQuest and CEU Mediator 3.0.

Category	Compound Name	Molecular Formula	KEGG ID	Bioactivities
Fatty Acids	Citronellic acid	$C_{10}H_{18}O_2$	C16462	Anti-inflammatory, Antimicrobial
	Farnesoic acid	$C_{15}H_{24}O_2$	C16502	Anti-inflammatory, Anticancer
	Tetradecanedioic acid	$C_{14}H_{26}O_4$	HMDB00872	Skin disorders treatment
Alcohols	Coniferyl alcohol	$C_{10}H_{12}O_3$	C00590	Antioxidant, Anti-inflammatory
	Capsidiol	$C_{15}H_{24}O_2$	C09627	Antimicrobial
	6-endo-Hydroxycineole	$C_{10}H_{18}O_2$	C03092	Anti-inflammatory
Esters	N-gamma-Nitro-L-arginine methyl ester	$C_7H_{15}N_5O_4$	C04337	Nitric oxide synthase inhibitor
	Methyl 2-diazoacetamidohexanoate	$C_9H_{16}N_3O_3$	C04130	Antibacterial
Amino Acid Derivatives	Hercynine	$C_9H_{16}N_3O_2$	C05575	Antioxidant
	N^4-Phosphoagmatine	$C_5H_{15}N_4O_3P$	C02726	Neuroprotective
Terpenes	Latia luciferin	$C_{15}H_{24}O_2$	C02293	Bioluminescent marker
	Limonene-1,2-diol	$C_{10}H_{18}O_2$	C07276	Antioxidant
Phenols	4-tert-Pentylphenol	$C_{11}H_{16}O$	C14226	Antioxidant
	Jasmone	$C_{11}H_{16}O$	C08490	Antimicrobial, Fragrance
Alkaloid	Nupharamine	$C_{15}H_{25}NO_2$	C17464	Potential therapeutic agent
	Indole	C_8H_7N	C00463	Antioxidant, Anti-inflammatory, Anti-tuberculosis
	2-Phenylethanol	$C_8H_{10}O$	C05853	Antioxidant, Anti-inflammatory, Anti-tuberculosis
Antimitotic Agent	Curacin A	$C_{23}H_{35}NOS$	C11689	Antimitotic activity
Antineoplastic Agent	Epothilone C	$C_{26}H_{39}NO_5S$	C15694	Anticancer activity
Antibiotic	Tiamulin	$C_{28}H_{47}NO_4S$	C12065	Antibacterial activity
Antibiotic Precursor	Demethyl-desacetyl-rifamycin S	$C_{34}H_{41}NO_{11}$	C14724	Precursor for antibiotic synthesis
Flavonoid	1-Methoxy-4-(2-propenyl)benzene	$C_{10}H_{12}O$	C10452	Antioxidant
Phenylpropanoids	Anethole	$C_{10}H_{12}O$	C10428	Antioxidant

Phenylpropanoids	Isovitexin	$C_{21}H_{20}O_{10}$	C01714	Antioxidant, Anti-inflammatory,Antimicrobial, Wound healing, Vasodilation
Phenylpropanoids	Vitexin	$C_{21}H_{20}O_{10}$	C01460	Antioxidant, anti diabetic, improvement of insulin resistance
Flavone and flavonols	Cosmosiin	$C_{21}H_{20}O_{10}$	C04608	Antioxidant
Flavone and flavonols	Afzelin	$C_{21}H_{20}O_{10}$	C16911	Antioxidant
Flavone and flavonols	Quercitrin	$C_{21}H_{20}O_{11}$	C01750	Antioxidant
Flavone and flavonols	Luteolin 7-glucoside	$C_{21}H_{20}O_{11}$	C03951	Antibacterial effects
Flavone and flavonols	Kaempferol-3-O-galactoside	$C_{21}H_{20}O_{11}$	C12626	Antioxidant
Flavone and flavonols	Luteolin 7-O-glucuronide	$C_{21}H_{18}O_{12}$	C03515	Antioxidant
Flavonoids	Aureusidin 6-O-glucoside	$C_{21}H_{20}O_{11}$	C16409	Antioxidant
Carotenoids	Isorenieratene/ (Leprotene)	$C_{40}H_{48}$	C15943	Antioxidant

Table S4. *In silico* Level 3 metabolite annotations as generated by SIRIUS 5.6.3 for sample specific metabolties. A zodiac score (over 95%) and a similarity score (over 75%) were chosen as cutoffs for this table.

**Compound**	**Sample Types Detected**	**Experimental Mass**	**Molecular Formula**	**Zodiac Score [%]**	**Similarity [% SIRIUS Score]**	**R. T**	**Fragments**	**Ontology: Compound classes/biochemical pathways**
Heriguard	SE, TE	355.1011	$C_{16}H_{18}O_9$	100	100	3.22	135.0441; 145.0285; 163.0382; 181.0486	Quinic acids and derivatives; cinnamic acids and derivatives; phenylpropanoids
3-O-Feruloylquinic acid	SE, TE	369.1172	$C_{17}H_{20}O_9$	100	98.13	3.94	137.0229; 145.0282; 177.0543	Quinic acids and derivatives; cinnamic acids and derivatives; phenylpropanoids
Roseoside	All	387.1998	$C_{19}H_{30}O_8$	100	96.09	4.99	95.0856 123.0793; 149.0954; 179.1439; 189.1274; 207.1372	Alkyl glycosides; Terpenoids
12-Deoxyphorbol 13-angelate	LE	431.2413	$C_{25}H_{34}O_6$	99.95	90.1	10.03	83.0490; 179.0848; 267.1719; 277.1596; 295.1677; 313.1790	Tetracyclic diterpenoids; terpenoids; tigliane and ingenane diterpenoids
Reinutrin	SE, TE	435.0907	$C_{20}H_{18}O_{11}$	97.085	99.69	6.33	61.0285; 73.0280; 115.0387; 303.0505	Flavonoid-3-O-glycosides; flavonoids
Trifolin (Kaempferol 3-O-rutinoside)	SE, TE	449.1057	$C_{21}H_{20}O_{11}$	93.467	99.69	6.41	85.0282; 127.0393; 145.0483; 287.0524	Flavonoid-3-O-glycosides; flavonoids
Hirsutrin	SE	465.1016	$C_{21}H_{20}O_{12}$	98.291	100	6.16	97.0283; 109.0283; 145.0499; 303.0504	Flavonoid-3-O-glycosides; flavonoids
Mikwelianin	SE	479.0809	$C_{21}H_{18}O_{13}$	99.44	98.3	6.28	113.0225; 131.0338; 141.0172;	Flavonoid-3-O-glucuuronides; flavonoids; shikimates and phenylproanoids

							159.0288; 303.0504	
methyl 2-[(2S)-3-oxo-2-[(Z)-pent-2-enyl]cyclopentyl]acetate	SE, TE	225.1477	$C_{13}H_{20}O_3$	100	96.13	11.32	81.0330; 147.1159; 175.1107; 193.1209	Jasmonic acids; cyclic ketones; octadecanoids
Coumarin	SE	147.0433	$C_9H_6O_2$	100	93.42	14.11	68.9966; 85.0281; 91.0538; 103.0535	Pyranones and derivatives; polyketides; 1-benzopyrans
Phyllanthusiin E	SE	293.0296	$C_{13}H_8O_8$	100	78.16	5.18	191.0334; 219.0281; 247.0223	Gallotannins; phenolic acids; shikimates and phenylpropanoids
Eriojaposide B	SE	517.2615	$C_{25}H_{40}O_{11}$	93.166	75.71	7.51	105.0180; 149.0956; 191.1416	Terpene glycosides; Terpenoids (Megastigmanes)

Table S5. *In silico* Level 3 metabolite annotations as generated by SIRIUS 5.6.3 for other metabolites.

Compound	Experimental Mass	Molecular Formula	Zodiac Score [%]	Similarity [% SIRIUS Score]	t_R	Fragments	Compound classes/biochemical pathways
Phlorone	137.0591	$C_8H_8O_2$	100.00	76.83	5.04	55.0181; 79.0539; 81.0695	Polyketides
D-Cavone	151.1113	$C_{10}H_{24}O$	100.00	72.57	14.93	95.0856; 109.0649; 123.0792; 133.0644	Monoterpenoids; Terpenoids
O-methylkojic acid	157.0487	$C_7H_8O_4$	100.00	82.58	4.48	68.0254; 85.0281; 142.0263;	Polyketides
Valerophenone	163.1112	$C_{11}H_{14}O$	100.00	74.38	8.58	105.0333; 107.0851; 135.0798; 145.1014	Phenylpropanoids
Coumarate	165.054	$C_9H_8O_3$	100.00	95.59	4.15	91.0536; 119.0489; 147.0434	Coumaric/cinnamic acids and derivatives; phenylpropanoids
Adermine	170.0798	$C_8H_{11}NO_3$	100.00	48.47	0.13	110.0600; 124.0746; 142.0859; 152.0693	Alkaloids
Lupinine	170.1534	$C_{10}H_{19}NO$	100.00	74.56	1.51	58.0651; 98.0957; 110.0600; 124.0747	Lysine alkaloids; Lupin alkaloids; alkaloids
alpha-Ionone	193.1573	$C_{13}H_{20}O$	100.00	78.46	6.13	135.1171; 151.1471; 165.1638; 175.1486;	Terpenoids
Ferulate	195.0644	$C_{10}H_{10}O_4$	100.00	96.13	4.93	125.0596; 145.0282; 163.0383; 177.0543	Coumaric/cinnamic acids and derivatives; phenylpropanoids
3-oxo-alpha-Ionone	207.137	C_{13}H18O2	100.00	84.06	11.39	133.1008; 147.1161;	Terpenoids

						149.0954;<br>189.1274	
Fratexin	209.0444	$C_{10}H_8O_5$	100.00	87.07	3.98	67.0542;<br>83.0492;<br>93.0695;<br>95.0487;	Coumarins; shikimates and phenylpropanoids
Jasmonate	211.1314	$C_{12}H_{18}O_3$	100.00	88.37	9.84	81.0330;<br>175.1109;<br>193.1213;	Jasmonic acids
Dehydrovomifoliol	223.1328	$C_{13}H_{18}O_3$	100.00	90.3	3.35	205.1219;<br>187.1111;<br>177.1277;<br>159.1162	Terpenoids
Vomifoliol	225.1474	$C_{13}H_{20}O_3$	100.00	64.8	2.85	149.0952;<br>121.1003;<br>207.1369;<br>119.0847	Terpenoids
Dinor-oxo-phytodienoic acid	265.1787	$C_{16}H_{24}O_3$	100	78.14	6.45	247.1694;<br>229.1575;<br>211.1466;<br>149.0955;<br>135.0798	Macrolides and analogues; phenylpropanoids and polyketides
Quertine/Quercetin	303.0503	$C_{15}H_{10}O_7$	96.23	93.19	6.41	285.0383;<br>257.0445;<br>229.0488;<br>137.0226	Flavonols; flavonoids; Shikimates and phenylpropanoids
Lobohedleolide	331.1877	$C_{20}H_{26}O_4$	100.00	73.28	7.79	285.1842;<br>255.1365;<br>227.1437;<br>175.1110	Diterpenoids; terpenoids
Bauhinin	344.1328	$C_{15}H_{21}NO_8$	98.79	68.11	2.65	165.0549;<br>147.0434;<br>85.0283;<br>97.0284	Cyanogenic glycosides
Ingenol	349.2031	$C_{20}H_{28}O_5$	100.00	73.97	5.55	313.1788;<br>295.1676;<br>285.1844;<br>161.0954	Diterpenoids; terpenoids
Roseoside	387.1998	$C_{19}H_{30}O_8$	100	96.09	4.99	95.0856;<br>123.0793;<br>149.0954;<br>179.1439;	Alkyl glycosides; Terpenoids

		189.1274;<br>207.1372	

Table S6. Level 2 metabolite annotations as generated by MS-DIAL 5.1 using various downloadable public databases in positive ionization mode.

Metabolite	Adduct	Chemical Formula	Precursor mass	MS/MS Fragments	Sample Type
Isatin	$[M+H]^+$	$C_8H_5NO_2$	148.03880	92.0490; 120.04366; 130.02864; 148.03856	SE, LE
Tributylamine	$[M+H]^+$	$C_{12}H_{27}N$	186.22069	57.0699; 130.1589	SE
Chlorogenic acid	$[M+H]^+$	$C_{16}H_{18}O_9$	355.10101	135.04376; 145.02789; 163.0384	SE
Luteolin	$[M+H]^+$	$C_{15}H_{10}O_6$	287.05371	90.9570; 287.0541	SE, TE
Eudesmin	$[M+Na]^+$	$C_{22}H_{26}O_6$	409.1608	289.1034; 409.1612	LE
Cuminyl Alcohol	$[M+H-H_2O]$	$C_{10}H_{14}O$	133.10075	79.0540; 105.0696; 133.1009	TE
N^6-threonylcarbanoyladenosine	$[M+H]^+$	$C_{15}H_{20}N_6O_8$	413.13889	120.0650; 136.0615; 162.0400	SE
Afzelin (Kaempferol-3-rhamnoside)	$[M+H]^+$	$C_{21}H_{20}O_{10}$	433.11108	71.0500; 85.0281; 129.05434; 287.0541	SE, TE
Kaempferol-3-O-rutinoside	$[M+H]^+$	$C_{27}H_{30}O_{15}$	595.16290	71.04903; 85.0281; 129.05457; 287.0541	SE
Rutin (Quercetin rutinoside; Rutoside)	$[M+H]^+$	$C_{27}H_{30}O_{16}$	611.15851	71.0500; 85.0281; 127.0387; 303.0490	SE

Table S7. Level 2 annotation of selected compounds derived from feature-based molecular networking via GNPS with fragmentation data grouped according to natural product classification. Other annotated metabolites not listed in this table can be viewed at: https://gnps.ucsd.edu/ProteoSAFe/status.jsp?task=685a12e4b3674ae0baebafcd1fd3f3de

Metabolite	Adduct	Chemical Formula	Precursor m/z	MS/MS Fragments	Natural Product Classifier Pathway
N-(-)-Jasmonoyl-(S)-leucine	$[M+H]^+$	$C_{14}H_{24}N_2O_3$	324.22	132.1; 147.12; 151.1	Amino acids and derivatives
N^6-Isopentenyladenosine	$[M+H]^+$	$C_{14}H_{24}N_5O_5$	336.17	138.06; 148.06; 204.1	Nucleosides, nucleotides and analogues
Salicylic acid	$[M+H]^+$	$C_7H_6O_3$	139.04	85.04; 93.07; 111.0	Shikimates and Phenylpropanoids
p-Coumaric acid	$[M+H-H_2O]^+$	$C_9H_8O_3$	147.04	91.05; 119.0; 123.96	Shikimates and Phenylpropanoids
4-hydroxy-3-methoxycinnamaldehyde	$[M+H]^+$	$C_{10}H_{10}O_3$	161.06	105.0; 115.05; 133.06	Shikimates and Phenylpropanoids
Caffeic acid	$[M+H-H_2O]^+$	$C_9H_8O_4$	163.04	89.04; 107.05; 117.03; 135.04; 145.03	Shikimates and Phenylpropanoids
3-hydroxycinnamic acid	$[M+H]^+$	$C_9H_8O_3$	163.06	93.07; 119.05	Shikimates and Phenylpropanoids
Ferulic acid	$[M+H-H_2O]^+$	$C_{10}H_{10}O_4$	177.05	117.03; 145.0; 177.05	Shikimates and Phenylpropanoids
Sinapyl alcohol	$[M+H-H_2O]^+$	$C_{10}H_{12}O_2$	193.09	105.07; 111.0; 133.06	Shikimates and Phenylpropanoids
Quercetin	$[M+H]^+$	$C_{18}H_{14}O_3$	303.05	137.02; 165.02; 229.05	Shikimates and Phenylpropanoids
Trans-*p*-coumaric acid-4-O-glucopyranoside	$[M+H]^+$	$C_{14}H_{18}O_9$	327.12	147.0; 165.06	Shikimates and Phenylpropanoids
Chlorogenic acid	$[M+H]^+$	$C_{16}H_{18}O_9$	355.10	135.04; 145.03; 163.0	Shikimates and Phenylpropanoids
Feruloyl quinic acid	$[M+H]^+$	$C_{24}H_{24}O_{13}$	369.12	117.03; 145.02; 177.0	Shikimates and Phenylpropanoids
Afzelin	$[M+H]^+$	$C_{24}H_{24}O_{12}$	433.11	71.05; 85.03; 287.0	Shikimates and Phenylpropanoids
Kaempferol-4-glucoside	$[M+H]^+$	$C_{24}H_{24}O_{12}$	449.11	86.03; 127.04; 145.05; 287.0	Shikimates and Phenylpropanoids
Kaempferol-3-gluconoride	$[M+H]^+$	$C_{24}H_{24}O_{13}$	463.08	86.03; 113.02; 287.0	Shikimates and Phenylpropanoids
Quercetin-3-O-gluconoride	$[M+H]^+$	$C_{24}H_{24}O_{13}$	479.08	86.03; 159.03; 303.0	Shikimates and Phenylpropanoids

(-)-Myrtenal	$[M+H]^+$	$C_{10}H_{14}O_2$	151.11	81.07; 93.07; 105.07; 109.06	Terpenoids
Loliolide	$[M+H]^+$	$C_{10}H_{14}O_2$	197.12	107.08; 135.12; 161.1; 179.1	Terpenoids

Table S8. Fold change (FC) analysis of therapeutic metabolites detected in this study. Fold change was calculated using quantified peak areas with the treatment/control (n=5). Treatment/control corresponds to [Heated]/[Unheated] or [Latex]/[Unheated] comparisons of average intensities of each metabolite. Compounds were considered upregulated in each treatment (i.e., treatment/control) when there is a $log_2FC > 1$, or downregulated with a $log_2FC < -1$. Compounds that were not detected were not shown in the table.

Compounds	Fold change (FC) [Heated]/[Unheated]	log2(FC)	Up/downregulation	Fold change (FC) [Latex]/[Unheated]	log2(FC)	Up/downregulation
**Bridelionoside D**	4.63	2.21	↑	1.12	0.17	↑
**Caffeic acid**	Absent	-	-	Absent	-	-
**Chrysin**	2.12	1.08	↑	Absent	-	-
**Coumarin**	2.77	1.47	↑	Absent	-	-
**Ethyl linoleate**	12.82	3.68	↑	0.08	-3.70	↓
**Ferulic acid**	2.96	1.57	↑	Absent	-	-
**Fisetin**	1.65	0.72	↑	0.01	-6.90	↓
**Hyperoside**	0.83	-0.26	↓	Absent	-	-
**Myricetin**	0.52	-0.95	↓	Absent	-	-
**Neriifolin**	5.58	2.48	↑	124.44	6.96	↑
**Prostratin**	1.82	0.86	↑	29.81	4.90	↑
**Protocatechuic acid**	Absent	-	-	Absent	-	-
**Quercetin**	0.89	-0.17	↓	Absent	-	-
**Rutin**	0.93	-0.10	↓	Absent	-	-

Table S9. Other semi-targeted metabolites via the KNapSAcK database specific to the Euphorbia species. A custom database made from queried metabolites in KNapSAcK was built to screen the XCMS outline output for putative metabolites within a 5-ppm error margin using HormonomicsDB. This annotation would be at level 3. Isomeric compounds are separated by "//". It should be noted that for some compounds, they may have more than 3 isomers that may not be listed on the table for brevity. Peak quality for these queried compounds were not checked. It should be noted that to date, compounds specific to Euphorbia species in KNapSAcK or in literature may not be present in other databases.

No	Compound Name	Chemical Formula	Adduct	Exact Mass	Measured m/z	t_R	Sample Type Found
1	Kaempferol	$C_{15}H_{10}O_6$	$[M+H]^+$	287.0547	287.0556	5.95	All
2	Quercetin	$C_{15}H_{10}O_7$	$[M+H]^+$	303.0490	303.0505	6.97	All
3	trisnor-Isoespinenoxide	$C_{27}H_{44}O$	$[M+H]^+$	385.3488	385.3471	8.43	All
4	Icariside B2 // Citroside A // (6S,9R)-Roseoside	$C_{19}H_{30}O_8$	$[M+H]^+$	387.2017	387.2019	0.98	All
5	Prostratin	$C_{22}H_{30}O_6$	$[M+H]^+$	391.2105	391.2121	9.07	All
6	1,2-Benzenedicarboxylic acid, diisooctyl ester	$C_{24}H_{38}O_4$	$[M+H]^+$	391.2840	391.2848	5.96	All
7	Campesterol	$C_{28}H_{48}O$	$[M+H]^+$	401.3803	401.3784	10.61	All
8	Ingenol mebutate	$C_{25}H_{34}O_6$	$[M+H]^+$	431.2413	431.2434	9.07	All
9	Quercetin 3'-xyloside	$C_{20}H_{18}O_{11}$	$[M+H]^+$	435.0907	435.0928	6.33	SE, TE
10	Astragalin // Orientin // Trifolin	$C_{21}H_{20}O_{11}$	$[M+H]^+$	449.1064	449.1084	4.55	All
11	Geranylgeranyl diphosphate	$C_{20}H_{36}O_7P_2$	$[M+H]^+$	451.2025	451.2015	4.17	All
12	Kaempferol 3-glucuronide	$C_{21}H_{18}O_{12}$	$[M+H]^+$	463.0854	463.0877	6.45	All
13	Jolkinol B	$C_{21}H_{20}O_{12}$	$[M+H]^+$	465.2632	465.2641	0.97	All
14	Taxifolin 3-galactoside	$C_{21}H_{22}O_{12}$	$[M+H]^+$	467.1201	467.1190	7.92	All
15	Diterpenoid EF-D	$C_{27}H_{38}O_7$	$[M+H]^+$	475.2674	475.2696	10.60	All
16	Euphorbia factor L10	$C_{28}H_{42}O_6$	$[M+H]^+$	475.3078	475.3060	9.47	All
17	Miquelianin	$C_{21}H_{18}O_{13}$	$[M+H]^+$	479.0837	479.0826	7.56	All
18	Isomyricitrin	$C_{21}H_{20}O_{13}$	$[M+H]^+$	481.1001	481.0982	7.93	All
19	Euphoscopin E	$C_{29}H_{36}O_7$	$[M+H]^+$	497.2516	497.2539	5.32	All
20	5-O-(2'E,4'E-Decadienoyl)ingenol // Euphohelioscopin A	$C_{30}H_{42}O_6$	$[M+H]^+$	499.3076	499.3060	8.45	All

21	Leeaoside	$C_{24}H_{40}O_{11}$	$[M+H]^+$	505.2627	505.2649	6.15	All
22	3,3',4-tri-O-Methylellagic acid 4'-O-beta-D-glucopyranoside	$C_{23}H_{22}O_{13}$	$[M+H]^+$	507.1115	507.1139	6.49	SE, TE
23	Euphorheliosin B	$C_{26}H_{38}O_{10}$	$[M+H]^+$	511.2556	511.2543	5.87	All
24	12-O-2Z,4E-Octadienoyl-4-deoxyphorbol 13-acetate	$C_{30}H_{40}O_7$	$[M+H]^+$	513.2870	513.2852	6.43	All
25	Lathyrol-3,15-diacetate-5-benzoate	$C_{31}H_{38}O_7$	$[M+H]^+$	523.2721	523.2696	4.90	All
26	Esulatin E	$C_{28}H_{36}O_{10}$	$[M+H]^+$	533.2361	533.2387	7.64	SE, TE
27	8-O-Tigloylingol	$C_{29}H_{40}O_9$	$[M+H]^+$	533.2724	533.2751	8.73	All
28	Euphoscopin F	$C_{31}H_{38}O_8$	$[M+H]^+$	539.2618	539.2645	7.10	All
29	3,12-Diacetyl-7-angeloyl-8-methoxyingol	$C_{30}H_{42}O_9$	$[M+H]^+$	547.2926	547.2907	5.60	All
30	Euphorbia factor N1	$C_{34}H_{46}O_6$	$[M+H]^+$	551.3352	551.3373	10.08	All
31	Ingenol 3,20-dibenzoate	$C_{34}H_{36}O_7$	$[M+H]^+$	557.2546	557.2539	6.56	All
32	Cheiradone A or B	$C_{31}H_{38}O_{10}$	$[M+H]^+$	571.2569	571.2543	5.37	All
33	Nicotiflorin	$C_{27}H_{30}O_{15}$	$[M+H]^+$	595.1643	595.1663	5.55	All
34	3,7,12-Triacetyl-8-benzoylingol	$C_{33}H_{40}O_{10}$	$[M+H]^+$	597.2715	597.2700	6.60	All
35	Ingenol-3-myristinate	$C_{37}H_{58}O_6$	$[M+H]^+$	599.4338	599.4312	9.98	All
36	Kaempferol 3-(2''-galloylglucoside)	$C_{28}H_{24}O_{15}$	$[M+H]^+$	601.1165	601.1194	7.00	All
37	Rutin	$C_{27}H_{30}O_{16}$	$[M+H]^+$	611.1590	611.1612	6.06	All
38	Decipidone	$C_{32}H_{44}O_{12}$	$[M+H]^+$	621.2915	621.2911	7.53	All
39	Tithymalin // Quercetin 3,5-digalactoside	$C_{27}H_{30}O_{17}$	$[M+H]^+$	627.1536	627.1561	5.50	All
40	Esulatin D	$C_{32}H_{44}O_{13}$	$[M+H]^+$	637.2846	637.2860	6.40	All
41	Quercetin 3-glucuronide-7-glucoside	$C_{27}H_{28}O_{18}$	$[M+H]^+$	641.1336	641.1354	5.89	SE, TE
42	Euphoscopin C	$C_{38}H_{44}O_9$	$[M+H]^+$	645.3075	645.3064	6.43	All
43	Euphoheliosnoid A or B	$C_{37}H_{43}NO_9$	$[M+H]^+$	646.3042	646.3016	6.55	All
44	Kansuinin G	$C_{34}H_{43}NO_{13}$	$[M+H]^+$	674.2837	674.2813	5.05	All

Table S10. Other semi-targeted metabolites via the KNapSAcK database specific to the Euphorbia species at annotation level 3 depicting up-or-downregulation of heated/traditional and latex extracts with respect to unheated/simple extracts. Compounds were considered upregulated in each treatment (i.e., [treatment]/[control]) when there is a $log_2FC > 1$, or downregulated with a $log_2FC < -1$.

Compound Name	Chemical Formula	Adduct	Exact Mass	Log_2FC-[Heated]/[Unheated]	Log_2FC-[Latex]/[Unheated]
Kaempferol	$C_{15}H_{10}O_6$	$[M+H]^+$	287.0547	-0.13	-5.01
Quercetin	$C_{15}H_{10}O_7$	$[M+H]^+$	303.0490	-1.11	-10.77
trisnor-Isoespinenoxide	$C_{27}H_{44}O$	$[M+H]^+$	385.3488	-4.45	-1.73
Icariside B2 // Citroside A // (6S,9R)-Roseoside	$C_{19}H_{30}O_8$	$[M+H]^+$	387.2017	-2.71	0.19
Prostratin	$C_{22}H_{30}O_6$	$[M+H]^+$	391.2105	0.06	3.03
1,2-Benzenedicarboxylic acid, diisooctyl ester	$C_{24}H_{38}O_4$	$[M+H]^+$	391.2840	0.51	-0.03
Campesterol	$C_{28}H_{48}O$	$[M+H]^+$	401.3803	-1.47	-1.61
Ingenol mebutate	$C_{25}H_{34}O_6$	$[M+H]^+$	431.2413	2.55	3.95
Quercetin 3'-xyloside	$C_{20}H_{18}O_{11}$	$[M+H]^+$	435.0907	-1.31	-
Astragalin // Orientin // Trifolin	$C_{21}H_{20}O_{11}$	$[M+H]^+$	449.1064	2.33	-9.25
Geranylgeranyl diphosphate	$C_{20}H_{36}O_7P_2$	$[M+H]^+$	451.2025	7.25	2.93
Kaempferol 3-glucuronide	$C_{21}H_{18}O_{12}$	$[M+H]^+$	463.0854	-0.73	-10.49
Jolkinol B	$C_{21}H_{20}O_{12}$	$[M+H]^+$	465.2632	6.79	7.81
Taxifolin 3-galactoside	$C_{21}H_{22}O_{12}$	$[M+H]^+$	467.1201	4.68	5.53
Diterpenoid EF-D	$C_{27}H_{38}O_7$	$[M+H]^+$	475.2674	-1.80	1.64
Euphorbia factor L10	$C_{28}H_{42}O_6$	$[M+H]^+$	475.3078	-0.34	-0.50
Miquelianin	$C_{21}H_{18}O_{13}$	$[M+H]^+$	479.0837	-1.07	-0.26
Isomyricitrin	$C_{21}H_{20}O_{13}$	$[M+H]^+$	481.1001	3.38	1.85
Euphoscopin E	$C_{29}H_{36}O_7$	$[M+H]^+$	497.2516	1.80	2.19
5-O-(2'E,4'E-Decadienoyl)ingenol // Euphohelioscopin A	$C_{30}H_{42}O_6$	$[M+H]^+$	499.3076	3.41	1.65
Leeaoside	$C_{24}H_{40}O_{11}$	$[M+H]^+$	505.2627	2.53	2.66
3,3',4-tri-O-Methylellagic acid 4'-O-beta-D-glucopyranoside	$C_{23}H_{22}O_{13}$	$[M+H]^+$	507.1115	-4.82	-0.06
Euphorheliosin B	$C_{26}H_{38}O_{10}$	$[M+H]^+$	511.2556	2.17	-2.60

12-O-2Z,4E-Octadienoyl-4-deoxyphorbol 13-acetate	$C_{30}H_{40}O_7$	$[M+H]^+$	513.2870	1.25	-0.18
Lathyrol-3,15-diacetate-5-benzoate	$C_{31}H_{38}O_7$	$[M+H]^+$	523.2721	-0.50	-0.04
Esulatin E	$C_{28}H_{36}O_{10}$	$[M+H]^+$	533.2361	3.62	-
8-O-Tigloylingol	$C_{29}H_{40}O_9$	$[M+H]^+$	533.2724	3.84	5.26
Euphoscopin F	$C_{31}H_{38}O_8$	$[M+H]^+$	539.2618	2.66	-1.65
3,12-Diacetyl-7-angeloyl-8-methoxyingol	$C_{30}H_{42}O_9$	$[M+H]^+$	547.2926	0.74	-2.53
Euphorbia factor N1	$C_{34}H_{46}O_6$	$[M+H]^+$	551.3352	5.31	1.70
Ingenol 3,20-dibenzoate	$C_{34}H_{36}O_7$	$[M+H]^+$	557.2546	2.92	3.82
Cheiradone A or B	$C_{31}H_{38}O_{10}$	$[M+H]^+$	571.2569	1.44	4.37
Nicotiflorin	$C_{27}H_{30}O_{15}$	$[M+H]^+$	595.1643	0.55	-3.09
3,7,12-Triacetyl-8-benzoylingol	$C_{33}H_{40}O_{10}$	$[M+H]^+$	597.2715	1.92	-4.24
Ingenol-3-myristinate	$C_{37}H_{58}O_6$	$[M+H]^+$	599.4338	6.19	-0.93
Kaempferol 3-(2''-galloylglucoside)	$C_{28}H_{24}O_{15}$	$[M+H]^+$	601.1165	-2.58	-5.72
Rutin	$C_{27}H_{30}O_{16}$	$[M+H]^+$	611.1590	-0.89	-9.06
Decipidone	$C_{32}H_{44}O_{12}$	$[M+H]^+$	621.2915	4.16	-3.77
Tithymalin // Quercetin 3,5-digalactoside	$C_{27}H_{30}O_{17}$	$[M+H]^+$	627.1536	1.63	2.01
Esulatin D	$C_{32}H_{44}O_{13}$	$[M+H]^+$	637.2846	0.41	-5.99
Quercetin 3-glucuronide-7-glucoside	$C_{27}H_{28}O_{18}$	$[M+H]^+$	641.1336	-1.87	-
Euphoscopin C	$C_{38}H_{44}O_9$	$[M+H]^+$	645.3075	2.84	-2.09
Euphoheliosnoid A or B	$C_{37}H_{43}NO_9$	$[M+H]^+$	646.3042	1.11	-3.94
Kansuinin G	$C_{34}H_{43}NO_{13}$	$[M+H]^+$	674.2837	2.82	2.56

Table S11. Level 3 annotation of putative metabolites via screening the top 15 masses of the VIP of heated (TE) vs unheated (SE) and latex (LE) vs unheated (SE) comparisons. CEU Mass Mediator (CMM 3.0) was used to query masses in positive ionization mode. Metabolites that were detected at level 2 confirmation in metabolite annotations via GNPS and MS-DIAL are indicated in the table.

Experimental mass	Adduct	m/z Error (ppm)	Molecular Weight	Name	Formula	VIP Comparison
135.0435	$[M+H]^+$	4	134.0368	LMSD{ FA 8:5 }	$C_8H_6O_2$	SE vs LE
	$[M+H-H_2O]^+$		152.0473	Orsellinaldehyde // Methoxytropolone // Menisdaurilide // Methyl-Hydroxyethylenepyran-One	$C_8H_8O_3$	
163.0384	$[M+H-H_2O]^+$	4	180.0423	Caffeate/Caffeic acid (level 2 confirmation)	$C_9H_8O_4$	SE vs LE
301.0333	$[M+H]^+$	3	300.027	Pseudopurpurin // Emodate	$C_{15}H_8O_7$	SE vs TE; SE vs LE
	$[M+H-H_2O]^+$		318.0376	Quercetagetin // Myricetin	$C_{15}H_{10}O_8$	
303.0488	$[M+H]^+$	4	302.0427	Quercetin (level 2 confirmation)	$C_{15}H_{10}O_7$	SE vs LE
391.1961	$[M+H]^+$	0	390.189	LMSD{ FA 18:3;O7 }	$C_{18}H_{30}O_9$	SE vs TE
433.1112	$[M+H]^+$	4	432.1057	Afzelin (level 2 confirmation)	$C_{21}H_{20}O_{10}$	SE vs LE
434.1146	$[M+H]^+$	1	433.1076	Oxoheptyl glucosinolate	$C_{14}H_{27}NO_{10}S_2$	SE vs LE
445.1243	$[M+H]^+$	0	444.1169	Hydroxyphenytoin glucuronide	$C_{21}H_{20}N_2O_9$	SE vs TE
449.1062	$[M+H]^+$	4	448.1006	Kaempferol-4-glucoside (level 2 confirmation)	$C_{21}H_{20}O_{11}$	SE vs LE
465.1009	$[M+H]^+$	4	464.0955	CCG-208508 / 3-[(2S,3R,4R)-5-[(1R)-1,2-dihydroxyethyl]-3,4-dihydroxyoxolan-2-yl]oxy-2-(3,4-dihydroxyphenyl)-5,7-dihydroxychromen-4-one (level 2 confirmation)	$C_{21}H_{20}O_{12}$	SE vs LE
479.0801	$[M+H]^+$	4	478.0747	Quercetin-3-O-glucuronide (level 2 confirmation)	$C_{21}H_{18}O_{13}$	SE vs LE
504.1265	$[M+H-H_2O]^+$	0	521.1295	Petunidin-(acetyl-galactoside) // Petunidin-(acetylglucoside)	$C_{24}H_{25}O_{13}$	SE vs LE

Table S12. *In silico* Level 3 annotation using MS2Compound showing candidates that are ranked 1 with the highest mS-scores. Annotation was done via the mzMine workflow.

FeatureID	Rank	Adduct	Charge	MS2 Compound ID	mS-score	Source ID	Source	Name	MonoMass	HMDB	PubChem
257	1	M+H	1	CID002197	2.66	BENZYL-ALCOHOL	BioCyc	benzyl alcohol	108.0575	HMDB03119	244
2724	1	M+H	1	PE_654	2.34	654	PhenolExplorer	Catechol	110.0368	-	289
89	1	M+H	1	CID000624	-1.82	DI-H-URACIL	BioCyc	5,6-dihydrouracil	114.0429	HMDB00076	649
377	1	M+H	1	PE_427	3.27	427	PhenolExplorer	Benzoic acid	122.0368	-	243
328	1	M+H	1	PE_707	3.34	707	PhenolExplorer	4-Ethylphenol	122.0732	-	31242
18	1	M+H	1	KG_C10164	-1.18	C10164	KEGG	Picolinic acid	123.0320	-	1018
161	1	M+H	1	KG_C11918	2.97	C11918	KEGG	Maltol	126.0317	-	8369
1143	1	M+H	1	KG_C10149	1.18	C10149	KEGG	Guvacine	127.0633	-	3532
2884	1	M+H	1	KG_C00408	1.02	C00408	KEGG	L-Pipecolate	129.0790	-	439227
17	1	M+H	1	KG_C00903	0.65	C00903	KEGG	Cinnamaldehyde	132.0575	-	637511
344	1	M+H	1	CID004929	3.20	CPD-9933	BioCyc	trans-anol	134.0732		5474441
1548	1	M+H	1	KG_C06575	3.72	C06575	KEGG	p-Cymene	134.1096	-	7463
2346	1	M+H	1	KG_C06224	3.15	C06224	KEGG	3,4-Dihydroxystyrene	136.0524	-	151398
544	1	M+H	1	PE_705	3.44	705	PhenolExplorer	4-Ethylcatechol	138.0681	-	70761
593	1	M+H	1	CID003650	0.79	CPD-10213	BioCyc	mesifurane	142.0630		25203051
1530	1	M+H	1	CID002927	2.46	NAPHTHOL	BioCyc	1-naphthol	144.0575	HMDB12138	7005
427	1	M+H	1	KG_C10160	0.28	C10160	KEGG	Myosmine	146.0844	-	442649
1168	1	M+H	1	KG_C06577	2.54	C06577	KEGG	4-Isopropylbenzaldehyde	148.0888	-	326
2100	1	M+H	1	KG_C09906	4.07	C09906	KEGG	Thujone	152.1201	-	261491
2169	1	M+H	1	PE_726	3.60	726	PhenolExplorer	Gallic aldehyde	154.0266	-	

1167	1	M+H	1	KG_C08283	-1.23	C08283	KEGG	Homostachydrine	157.1103	-	4479243
30	1	M+H	1	PE_642	3.68	642	PhenolExplorer	4-Hydroxycoumarin	162.0317	-	54682930
1690	1	M+H	1	CID003757	2.38	CPD-8636	BioCyc	4-hydroxybenzalacetone	162.0681		796857
1307	1	M+H	1	KG_C11357	2.23	C11357	KEGG	(-)-Anabasine	162.1157	-	205586
2563	1	M+H	1	KG_C09887	2.31	C09887	KEGG	Piquerol A	166.0994	-	161734
465	1	M+H	1	KG_C10284	-0.69	C10284	KEGG	Crotanecine	171.0895	-	394146
41	1	M+H	1	KG_C10656	1.26	C10656	KEGG	Deoxypeganine	172.1000	-	442894
295	1	M+H	1	KG_C09268	2.07	C09268	KEGG	Herniarin	176.0473	-	10748
2784	1	M+H	1	CID003314	1.39	CPD-12395	BioCyc	1-(methylsulfanyl)-6-aci-nitrohexane	177.0823		1.23E+08
2113	1	M+H	1	CID001429	1.34	CONIFERYL-ALDEHYDE	BioCyc	coniferaldehyde	178.0630		5280536
366	1	M+H	1	CID001807	1.74	CPD-6482	BioCyc	methyleugenol	178.0994	HMDB31864	7127
1317	1	M+H	1	PE_579	1.17	579	PhenolExplorer	Dihydrocaffeic acid	182.0579	-	348154
1043	1	M+H	1	KG_C06070	4.12	C06070	KEGG	Iridotrial	182.0943	-	53297344
1177	1	M+H	1	KG_C10356	0.21	C10356	KEGG	Otonecine	185.1052	-	5281740
584	1	M+H	1	CID001574	0.73	BIPHENYL-23-DIOL	BioCyc	biphenyl-2, 3-diol	186.0681		254
137	1	M+H	1	KG_C10733	1.50	C10733	KEGG	Peganine	188.0950	-	442929
582	1	M+H	1	KG_C10480	2.81	C10480	KEGG	Myristicin	192.0786	-	4276
2115	1	M+H	1	KG_C09909	2.57	C09909	KEGG	Thymyl acetate	192.1150	-	68252
25	1	M+H	1	CID003318	3.16	CPD-13371	BioCyc	pseudoionone	192.1514	HMDB32498	1757003
1200	1	M+H	1	KG_C17497	2.30	C17497	KEGG	Zingerone	194.0943	-	31211
166	1	M+H	1	PE_675	1.36	675	PhenolExplorer	3,4-DHPEA-AC	196.0736	-	
1142	1	M+H	1	KG_C20209	-0.81	C20209	KEGG	Meconic acid	199.9957	-	10347
222	1	M+H	1	KG_C10743	1.59	C10743	KEGG	Vasicinol	204.0899	-	442934
28	1	M+H	1	KG_C11350	3.78	C11350	KEGG	(+)-trans-alpha-Irone	206.1671	-	5281905
426	1	M+H	1	KG_C08491	4.61	C08491	KEGG	(-)-Jasmonic acid	210.1256	-	5281166
510	1	M+H	1	KG_C10269	2.59	C10269	KEGG	Lunularin	214.0994	-	181511
204	1	M+H	1	KG_C08309	0.84	C08309	KEGG	N,N-Dimethyl-5-methoxytryptamine	218.1419	-	1832

1296	1	M+H	1	KG_C09737	4.34	C09737	KEGG	Solavetivone	218.1671	-	442399
2128	1	M+H	1	CID004248	0.55	CPD-12404	BioCyc	1-(methylsulfanyl)-9-aci-nitrononane	219.1293		1.23E+08
1239	1	M+H	1	KG_C09673	4.78	C09673	KEGG	Glutinosone	220.1463	-	442371
458	1	M+H	1	KG_C09715	4.41	C09715	KEGG	Rishitin	222.1620	-	108064
1927	1	M+H	1	CID003129	3.14	CPD-7203	BioCyc	5,6-epoxy-3-hydroxy-9-apo-beta-caroten-9-one	224.1412		5371267
133	1	M+H	1	KG_C08310	2.20	C08310	KEGG	N-Methylmescaline	225.1365	-	138365
496	1	M+H	1	KG_C06424	3.02	C06424	KEGG	Tetradecanoic acid	228.2089	-	11005
477	1	M+H	1	KG_C09503	4.02	C09503	KEGG	Mexicanin E	232.1099	-	22226
950	1	M+H	1	KG_C09408	3.67	C09408	KEGG	Eremofrullanolide	232.1463	-	442209
782	1	M+H	1	KG_C09023	0.95	C09023	KEGG	Agroclavine	238.1470	-	73484
1127	1	M+H	1	CID002906	3.55	CPD-9319	BioCyc	geranylhydroquinone	246.1620		5281857
1152	1	M+H	1	KG_C09660	3.84	C09660	KEGG	Dehydrongaione	248.1412	-	442367
368	1	M+H	1	CID003124	3.93	CPD-7279	BioCyc	2-cis,4-trans-xanthoxin	250.1569		91820570
1229	1	M+H	1	KG_C16795	4.83	C16795	KEGG	Hydnocarpic acid	252.2089	-	164601
2324	1	M+H	1	CID001316	0.28	DIHYDRO-NEO-PTERIN	BioCyc	7,8-dihydroneopterin	255.0968	HMDB02275	65074
1568	1	M+H	1	KG_C00249	2.55	C00249	KEGG	Hexadecanoic acid	256.2402	-	985
216	1	M+H	1	KG_C08402	3.24	C08402	KEGG	Cicutoxin	258.1620	-	25265910
1172	1	M+H	1	KG_C09392	1.16	C09392	KEGG	8-Deoxylactucin	260.1049	-	442196
499	1	M+H	1	KG_C19994	3.50	C19994	KEGG	Virol B	262.1933	-	10753985
1144	1	M+H	1	KG_C09708	4.23	C09708	KEGG	Phomenone	264.1362	-	93306
1238	1	M+H	1	KG_C09702	4.20	C09702	KEGG	Mukaadial	266.1518	-	159088
1574	1	M+H	1	CID003473	2.81	CPD-8721	BioCyc	abietatriene	270.2348		6432211
1246	1	M+H	1	KG_C08328	1.57	C08328	KEGG	Cardiospermin	275.1005	-	179647
1970	1	M+H	1	KG_C10494	3.75	C10494	KEGG	[6]-Shogaol	276.1725	-	5281794
2927	1	M+H	1	KG_C10482	2.52	C10482	KEGG	6-Paradol	278.1882	-	94378
1164	1	M+H	1	KG_C09482	2.91	C09482	KEGG	Hymenoxon	282.1467	-	42295
79	1	M+H	1	KG_C08367	1.41	C08367	KEGG	(11E)-Octadecenoic acid	282.2559	-	5281127
1325	1	M+H	1	CID004396	4.00	CPD-8724	BioCyc	dehydroabietadienal	284.2140		25200548
2702	1	M+H	1	KG_C01530	5.06	C01530	KEGG	Octadecanoic acid	284.2715	-	5281

2	1	M+H	1	KG_C10041	1.96	C10041	KEGG	Fisetin	286.0477	-	5281614
2050	1	M+H	1	KG_C10610	2.95	C10610	KEGG	Pilosine	286.1317	-	442869
1247	1	M+H	1	CID003957	5.78	CPD-8714	BioCyc	palustradienol	288.2453		25200493
2670	1	M+H	1	KG_C09566	3.20	C09566	KEGG	epi-Tulipinolide	290.1518	-	5281505
2291	1	M+H	1	KG_C09094	4.10	C09094	KEGG	Geranylgeraniol	290.2610	-	5281365
1411	1	M+H	1	KG_C09709	2.93	C09709	KEGG	Phytuberin	294.1831	-	442387
1943	1	M+H	1	KG_C08366	4.19	C08366	KEGG	Sterculic acid	294.2559	-	12921
92	1	M+H	1	CID000903	2.45	5-METHYLTHIOADENOSINE	BioCyc	<i>S</i>-methyl-5'-thioadenosine	297.0896	HMDB01173	439176
2175	1	M+H	1	KG_C10136	2.95	C10136	KEGG	Cassine	297.2668	-	193405
94	1	M+H	1	KG_C10019	0.50	C10019	KEGG	Apigenin 7,4'-dimethyl ether	298.0841	-	5281601
1131	1	M+H	1	KG_C10461	-0.78	C10461	KEGG	4-Hydroxyhomopterocarpin	300.0998	-	442792
3	1	M+H	1	KG_C00389	1.73	C00389	KEGG	Quercetin	302.0427	-	5280343
1224	1	M+H	1	KG_C06087	4.36	C06087	KEGG	Abietate	302.2246	-	10569
1161	1	M+H	1	PE_775	-1.65	775	PhenolExplorer	4'-O-Methylepicatechin	304.0947	-	
240	1	M+H	1	KG_C09557	1.90	C09557	KEGG	Tenulin	306.1467	-	227830
53	1	M+H	1	KG_C09183	3.25	C09183	KEGG	Sclareol	308.2715	-	163263
122	1	M+H	1	KG_C20211	4.12	C20211	KEGG	Yakuchinone A	312.1725	-	133145
165	1	M+H	1	KG_C09191	2.34	C09191	KEGG	Fruticosonine	312.2202	-	442091
2301	1	M+H	1	KG_C11866	4.30	C11866	KEGG	2,3-Dehydro-gibberellin A9	314.1518	-	443459
2153	1	M+H	1	KG_C10806	0.23	C10806	KEGG	Funtumine	317.2719	-	101702
2015	1	M+H	1	PE_1023	1.90	1023	PhenolExplorer	4-Hydroxyenterodiol	318.1467	-	
1129	1	M+H	1	KG_C20212	3.64	C20212	KEGG	Steviol	318.2195	-	452967
2172	1	M+H	1	PE_773	1.72	773	PhenolExplorer	4'-O-Methylepigallocatechin	320.0896	-	176920
2193	1	M+H	1	KG_C05909	0.20	C05909	KEGG	Leucodelphinidin	322.0689	-	440835
2339	1	M+H	1	KG_C08588	4.77	C08588	KEGG	Crocetin	328.1675	-	5281232
2313	1	M+H	1	KG_C20218	3.95	C20218	KEGG	Cannabielsoin	330.2195	-	162113

2674	1	M+H	1	KG_C09067	0.08	C09067	KEGG	Callicarpone	332.1988	-	442007
2277	1	M+H	1	KG_C09175	3.30	C09175	KEGG	Portulal	336.2301	-	5281393
1982	1	M+H	1	KG_C08568	1.03	C08568	KEGG	Vulgaxanthin-I	339.1066	-	1.35E+08
1972	1	M+H	1	PE_486	0.05	486	PhenolExplorer	Caffeoyl glucose	342.0951	-	
14	1	M+H	1	KG_C10814	1.17	C10814	KEGG	Paravallarine	343.2511	-	442980
129	1	M+H	1	PE_624	4.15	624	PhenolExplorer	Anhydro-secoisolariciresinol	344.1624	-	
1373	1	M+H	1	CID004471	2.92	10-BETA-HYDROXYTAXA-42011-DIEN-5-ALPH	BioCyc	10beta-hydroxytaxa-4(20),11-dien-5alpha-yl acetate	346.2508		443488
1269	1	M+H	1	CID002840	1.44	CPD-4442	BioCyc	<i>cis</i>-zeatin riboside	351.1543		5354008
1520	1	M+H	1	CID002121	1.26	SCOPOLIN	BioCyc	scopolin	354.0951		346340
902	1	M+H	1	KG_C10207	-0.57	C10207	KEGG	Cyclokievitone	354.1103	-	156777
1925	1	M+H	1	KG_C10653	-1.69	C10653	KEGG	Buntanine	355.1420	-	5281838
1116	1	M+H	1	CID003287	0.26	CPD-15281	BioCyc	1-O-feruloyl-beta-D-glucose	357.1186		13962928
1642	1	M+H	1	KG_C09585	0.13	C09585	KEGG	Vernomygdin	364.1522	-	5281509
345	1	M+H	1	PE_478	1.42	478	PhenolExplorer	5-Feruloylquinic acid	368.1107	-	
604	1	M+H	1	KG_C11678	1.61	C11678	KEGG	Dialdehyde	368.1736	-	11953960
2272	1	M+H	1	KG_C09147	0.71	C09147	KEGG	Calanolide A	370.1780	-	64972
1319	1	M+H	1	KG_C20205	3.04	C20205	KEGG	Tanabalin	372.1937	-	10667003
1995	1	M+H	1	CID004726	-1.21	CPD-6562	BioCyc	DIMBOA-beta-D-glucoside	373.1009		90659202
315	1	M+H	1	KG_C09202	0.56	C09202	KEGG	Tripdiolide	376.1522	-	294491
1282	1	M+H	1	KG_C10546	0.86	C10546	KEGG	Austrobailignan 1	382.1053	-	325700
2167	1	M+H	1	KG_C10609	0.08	C10609	KEGG	Phyllospadine	383.1369	-	442868
1134	1	M+H	1	KG_C16709	1.00	C16709	KEGG	O-Methylandrocymbine	385.1889	-	15286666
43	1	M+H	1	KG_C09307	-0.07	C09307	KEGG	Pteryxin	386.1366	-	5281425
813	1	M+H	1	PE_1018	2.18	1018	PhenolExplorer	Gomisin M2	386.1729	-	16728078
1516	1	M+H	1	KG_C09760	2.25	C09760	KEGG	Kazinol A	394.2144	-	442414
330	1	M+H	1	KG_C09593	-1.00	C09593	KEGG	Narcotoline	399.1318	-	442330

2425	1	M+H	1	CID004805	0.71	CPD-17594	BioCyc	O4,O5--dimethylthujaplicatin	402.1679		641789
1237	1	M+H	1	KG_C09798	2.31	C09798	KEGG	Scandoside methyl ester	404.1319	-	442433
117	1	M+H	1	KG_C10341	0.51	C10341	KEGG	Lasiocarpine	411.2257	-	6325507
314	1	M+H	1	CID004169	2.47	CPD-9711	BioCyc	dalcochinin	412.1522		25203053
312	1	M+H	1	KG_C09103	-0.41	C09103	KEGG	Grayanotoxin I	412.2461	-	442034
2121	1	M+H	1	KG_C09435	1.70	C09435	KEGG	Euparotin acetate	418.1628	-	5281456
2075	1	M+H	1	KG_C08336	-0.70	C08336	KEGG	Neolinustatin	423.1741	-	119533
1459	1	M+H	1	KG_C09123	2.65	C09123	KEGG	Athamantin	430.1992	-	442051
20	1	M+H	1	KG_C01821	2.49	C01821	KEGG	Isoorientin	448.1006	-	114776
2635	1	M+H	1	KG_C10491	0.28	C10491	KEGG	Lonchocarpenin	448.1886	-	54699185
2297	1	M+H	1	CID004338	1.75	CPD-14732	BioCyc	linalyl 6-O-beta-D-xylopyranosyl-beta-D-glucopyranoside	448.2308		131802
2164	1	M+H	1	KG_C08890	-1.09	C08890	KEGG	Convallagenin A	448.3189	-	441880
1320	1	M+H	1	KG_C09697	2.16	C09697	KEGG	Laserpitin	450.2618	-	5281524
349	1	M+H	1	KG_C09405	2.90	C09405	KEGG	Enhydrin	464.1682	-	5281441
1400	1	M+H	1	KG_C09612	-0.51	C09612	KEGG	Psychotrine	464.2675	-	65380
2031	1	M+H	1	KG_C09765	1.55	C09765	KEGG	Agnuside	466.1475	-	442416
2132	1	M+H	1	KG_C09328	0.21	C09328	KEGG	Alangimarckine	475.2835	-	442159
1163	1	M+H	1	KG_C09327	0.22	C09327	KEGG	Alangicine	480.2624	-	442158
212	1	M+H	1	KG_C17507	2.01	C17507	KEGG	Palmatoside G	492.1995	-	184515
2187	1	M+H	1	KG_C12140	3.43	C12140	KEGG	Malvidin 3-O-glucoside	493.1346	-	443652
390	1	M+H	1	KG_C16724	0.88	C16724	KEGG	Strictosamide	498.2002	-	10345799
2112	1	M+H	1	KG_C10562	1.47	C10562	KEGG	Eudesobovatol A	504.3240	-	442837
1551	1	M+H	1	KG_C17814	1.72	C17814	KEGG	Gomisin B	514.2203	-	6438572
2352	1	M+H	1	KG_C08810	-0.41	C08810	KEGG	Ajugalactone	516.2723	-	181744
2125	1	M+H	1	PE_680	-0.75	680	PhenolExplorer	Ligstroside	524.1894	-	
1157	1	M+H	1	KG_C08767	1.24	C08767	KEGG	12alpha-Hydroxyamoorstatin	532.2308	-	5462416
2054	1	M+H	1	KG_C08869	1.86	C08869	KEGG	Helveticoside	534.2829	-	441860
2453	1	M+H	1	KG_C09296	0.13	C09296	KEGG	Archangelolide	548.2621	-	5281422

2067	1	M+H	1	KG_C10280	2.56	C10280	KEGG	Auriculine	559.3145	-	442714
1997	1	M+H	1	CID004340	0.10	CPD-9677	BioCyc	dalcochinin-8'-O-beta-glucoside	574.2050		90657709
2156	1	M+H	1	KG_C08793	1.29	C08793	KEGG	Cucurbitacin A	574.3142	-	5281315
179	1	M+H	1	KG_C09164	1.47	C09164	KEGG	Ergocristine	609.2951	-	31116
2472	1	M+H	1	CID003780	2.32	CPDQT-523	BioCyc	brassinolide-23-O-glucoside	641.3901		1.03E+08
2188	1	M+H	1	KG_C09096	1.31	C09096	KEGG	Gnididin	648.2934	-	5281367
170	1	M+H	1	PE_75	0.99	75	PhenolExplorer	Malvidin 3-O-(6''-caffeoyl-glucoside)	655.1663	-	
